# Supplementary figures and images for: How are age-related differences in sleep quality associated with health outcomes? An epidemiological investigation in a UK cohort of 2406 adults
Source: BMJ Open. 2017 Jul 31;7(7):e014920. doi: 10.1136/bmjopen-2016-014920 (PMC5642766; doi:10.1136/bmjopen-2016-014920)

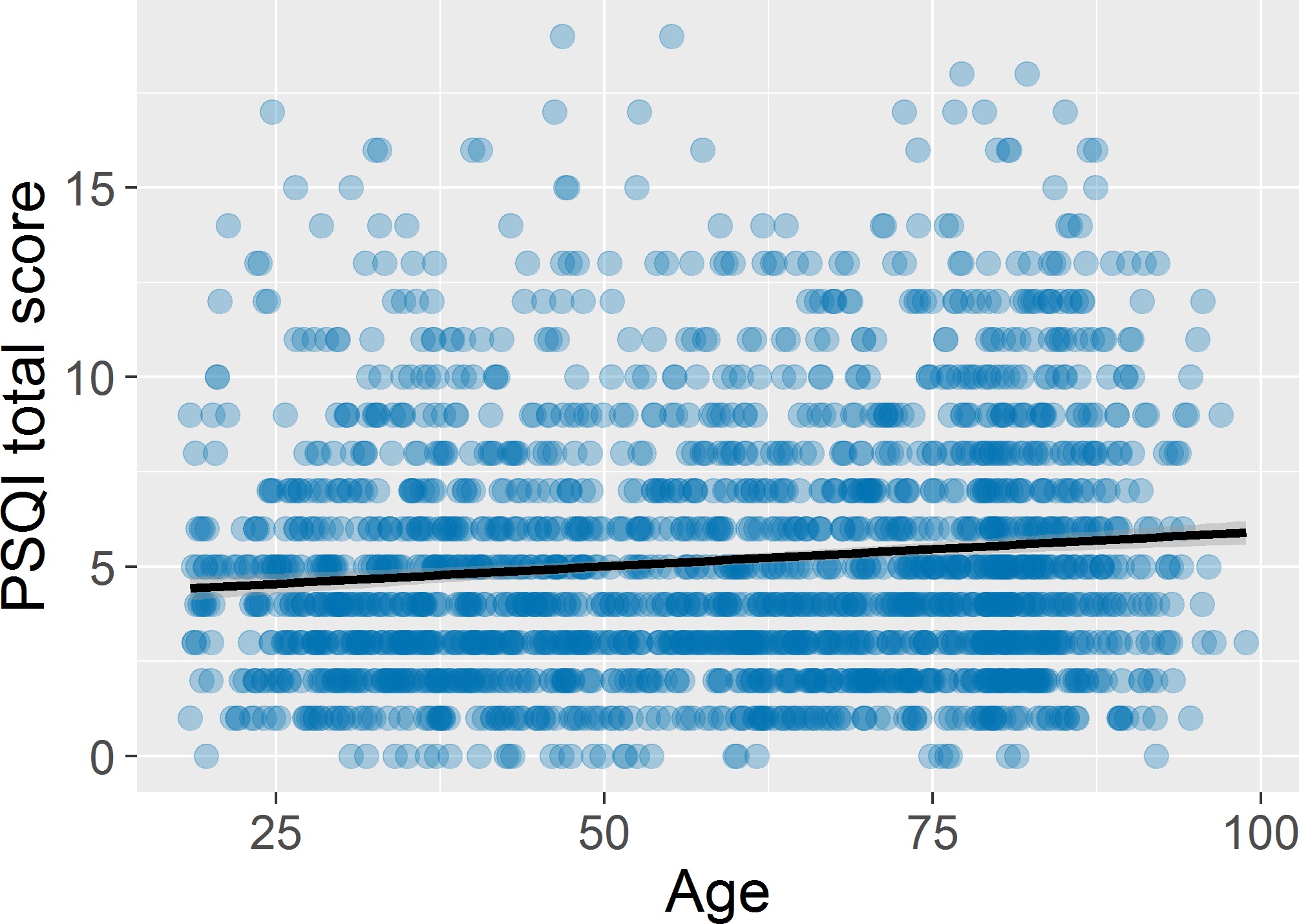

Supplement: Supplementary Figure 1 [file bmjopen-2016-014920supp001.jpg]

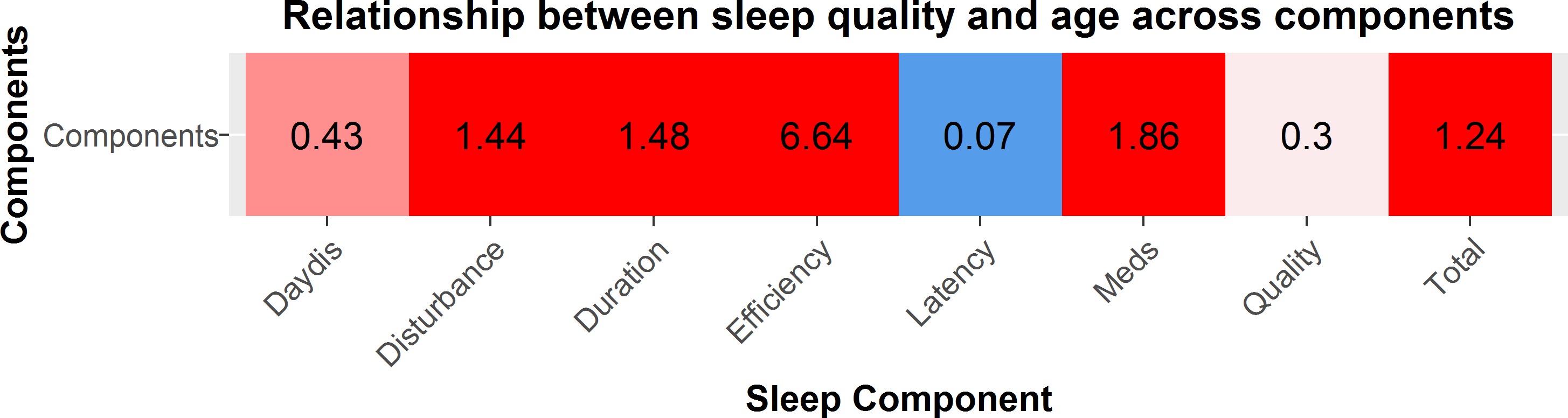

Supplement: Supplementary Figure 2 [file bmjopen-2016-014920supp002.jpg]

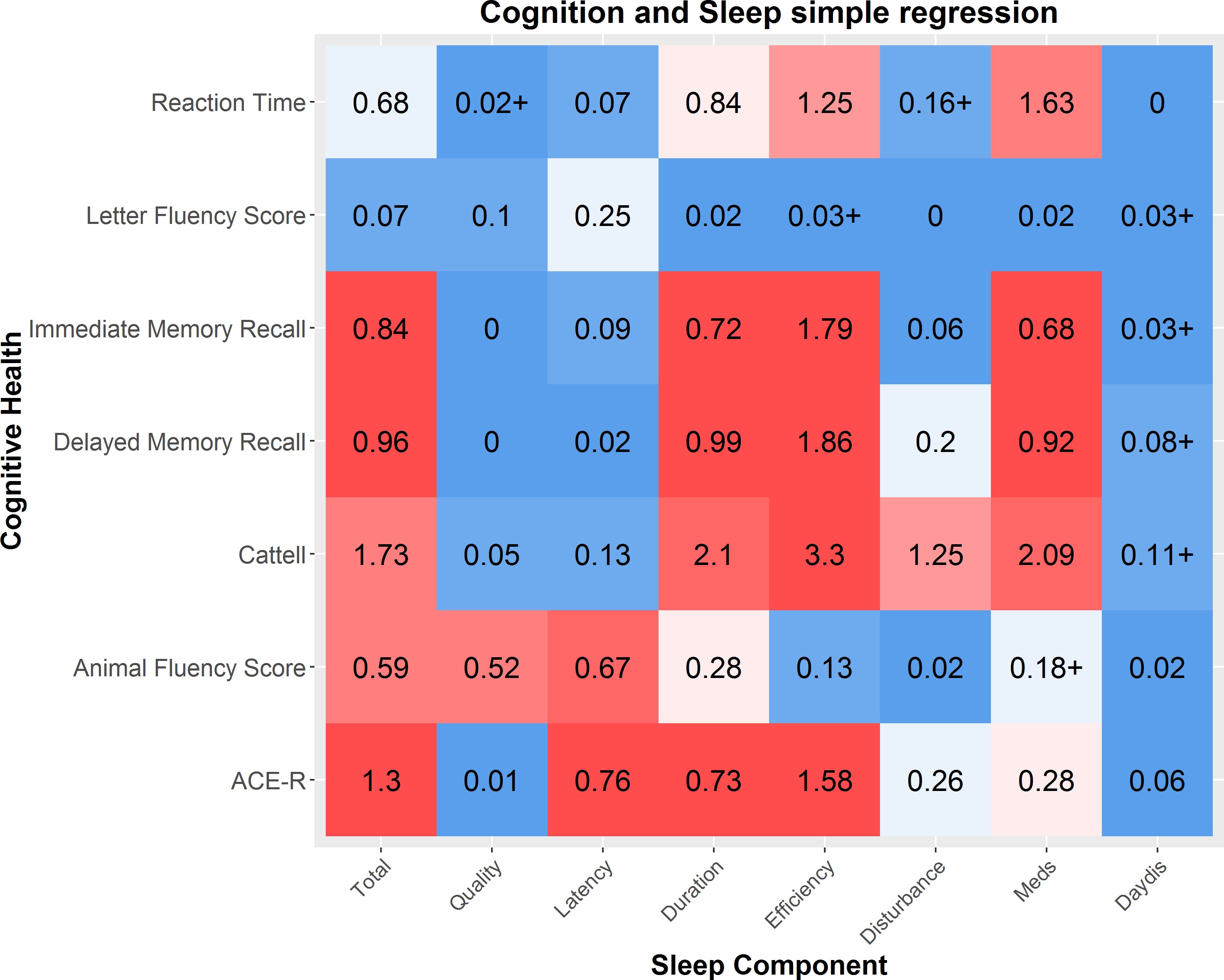

Supplement: Supplementary Figure 3 [file bmjopen-2016-014920supp003.jpg]

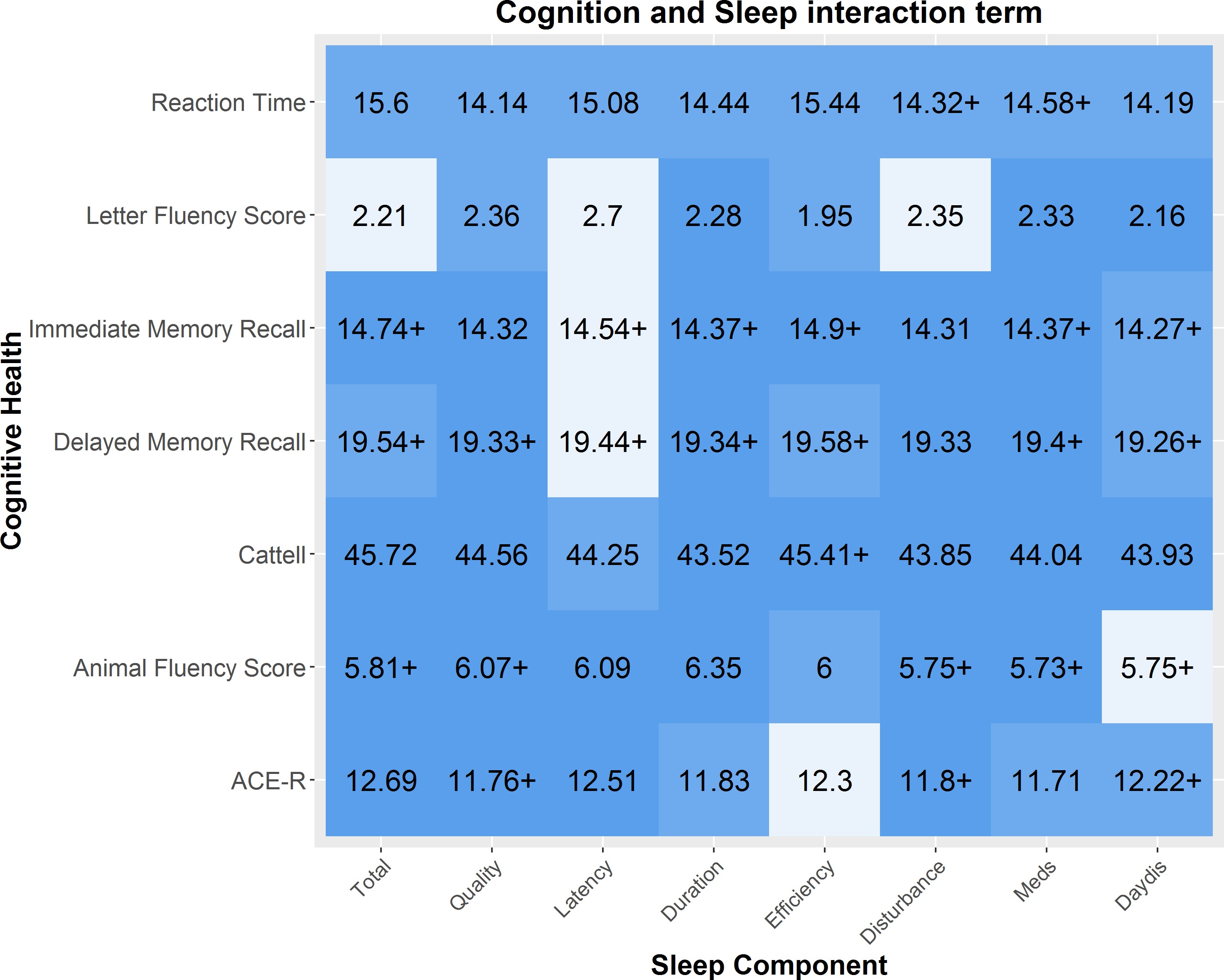

Supplement: Supplementary Figure 4 [file bmjopen-2016-014920supp004.jpg]

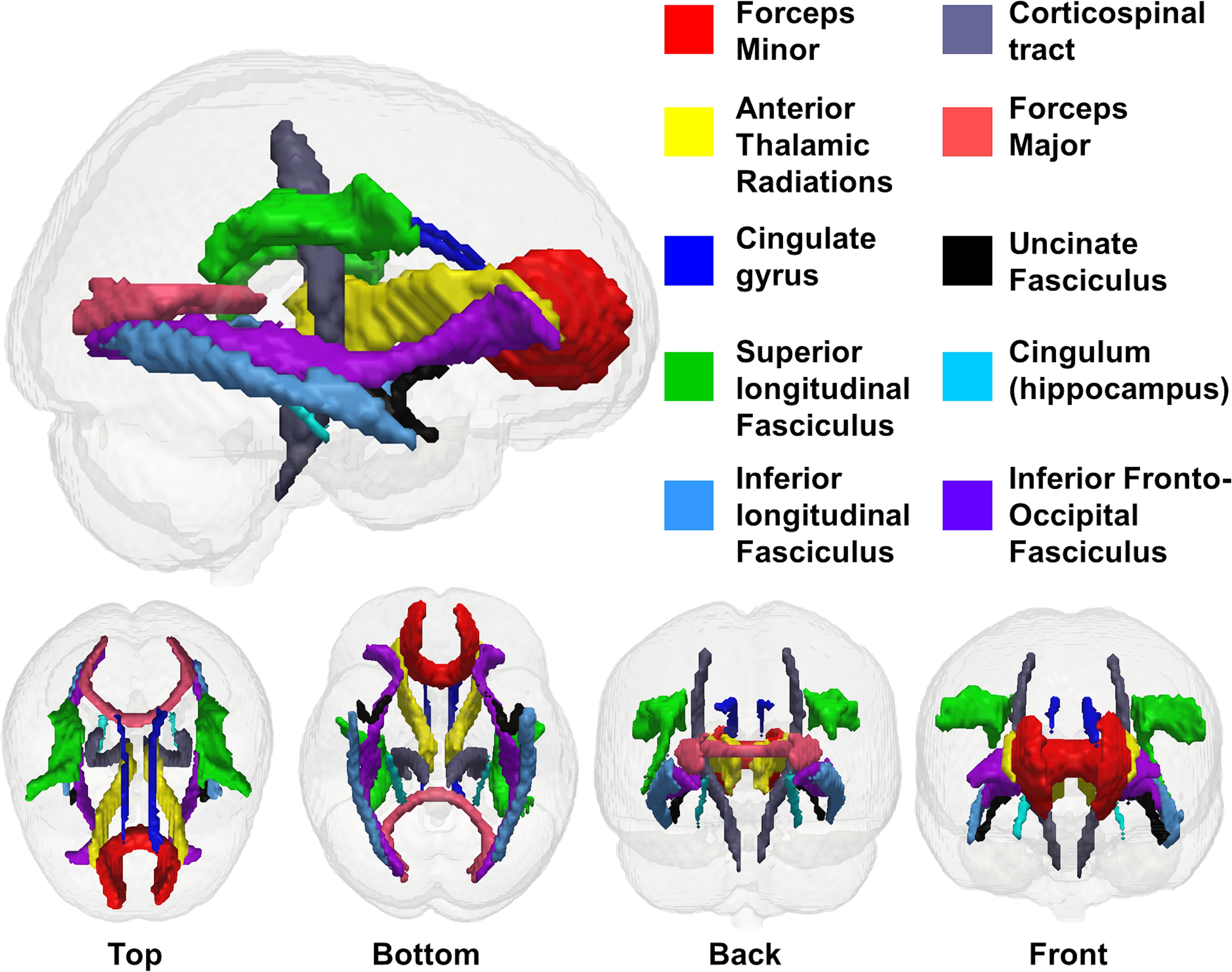

Supplement: Supplementary Figure 5 [file bmjopen-2016-014920supp005.jpg]

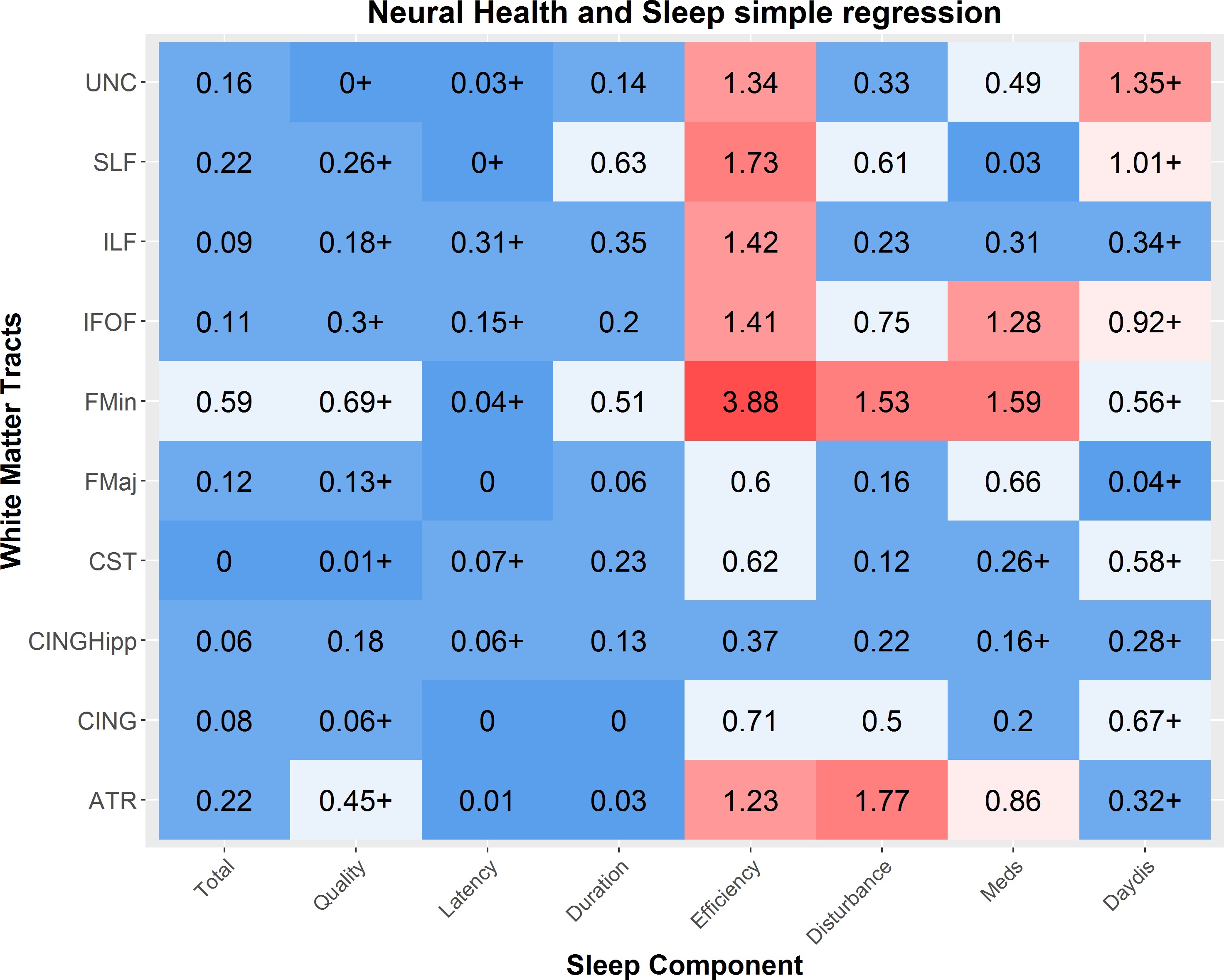

Supplement: Supplementary Figure 6 [file bmjopen-2016-014920supp006.jpg]

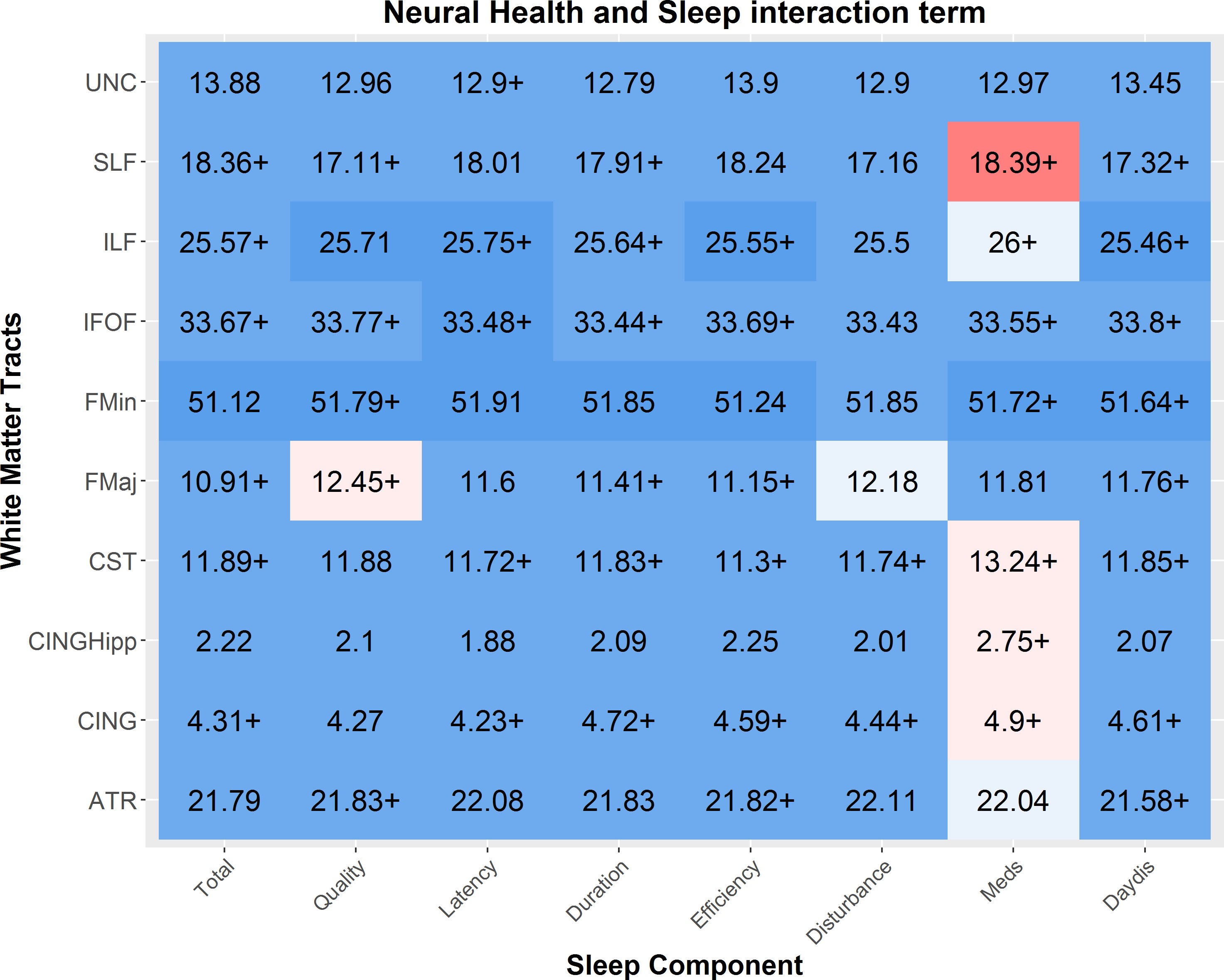

Supplement: Supplementary Figure 7 [file bmjopen-2016-014920supp007.jpg]

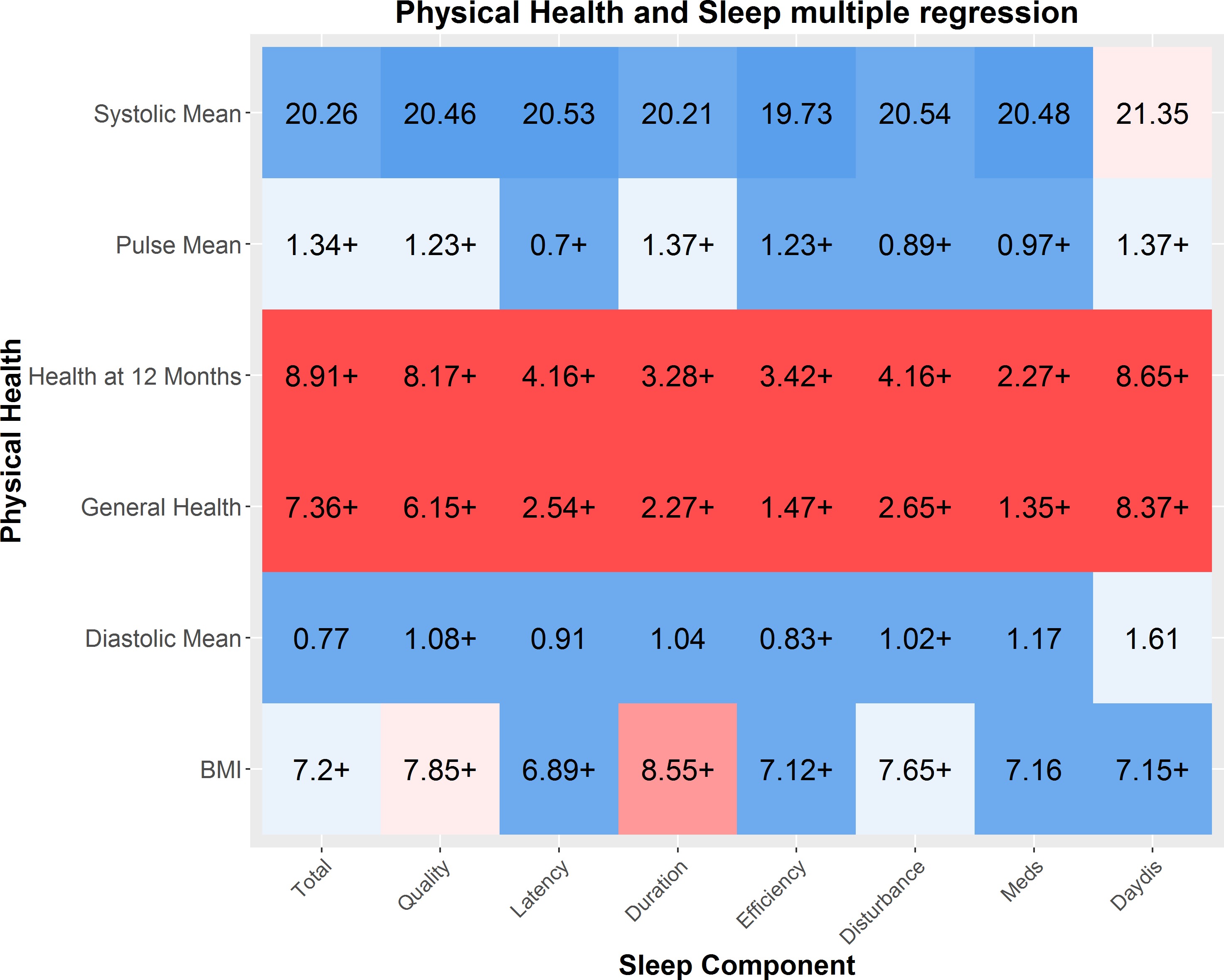

Supplement: Supplementary Figure 8 [file bmjopen-2016-014920supp008.jpg]

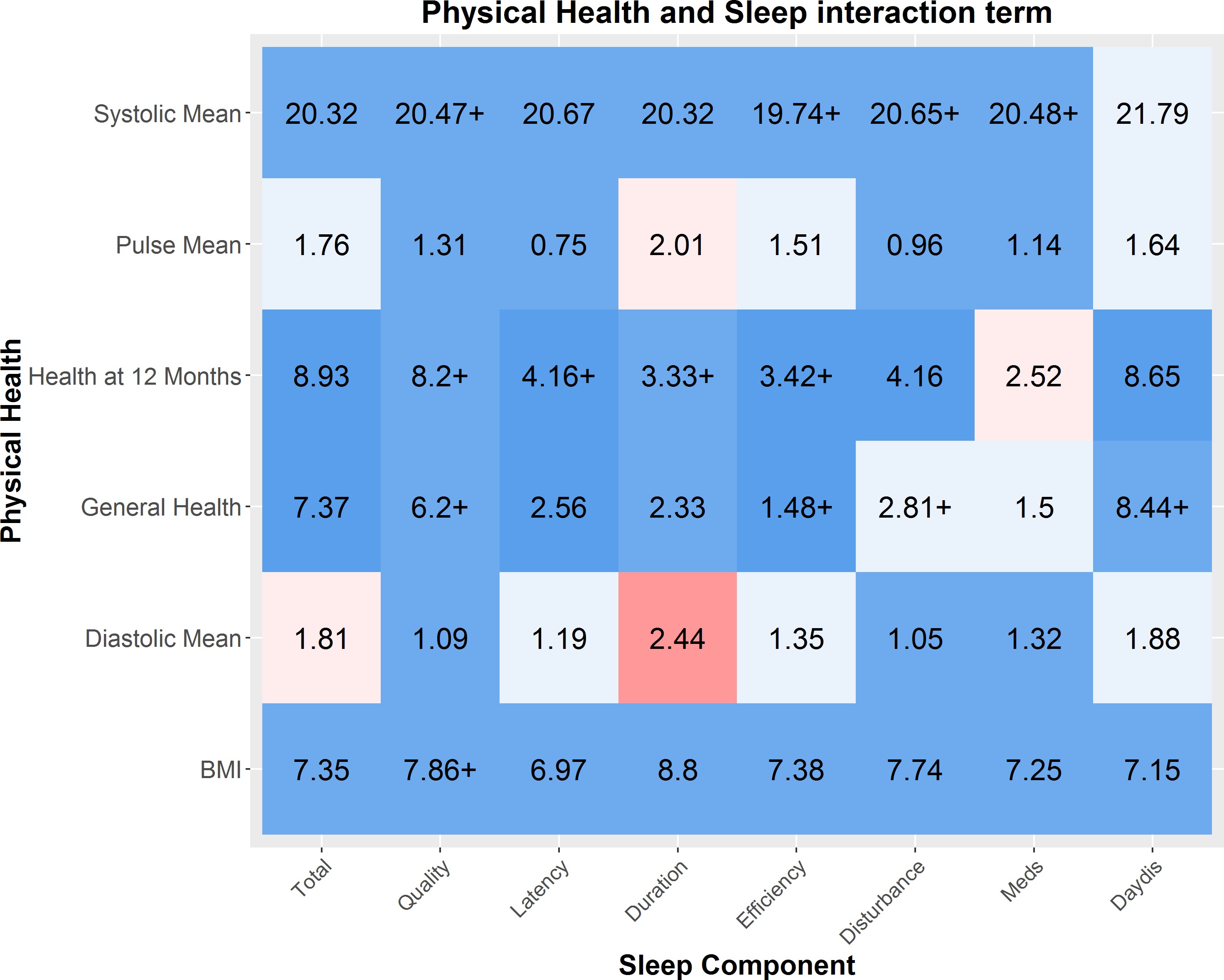

Supplement: Supplementary Figure 9 [file bmjopen-2016-014920supp009.jpg]

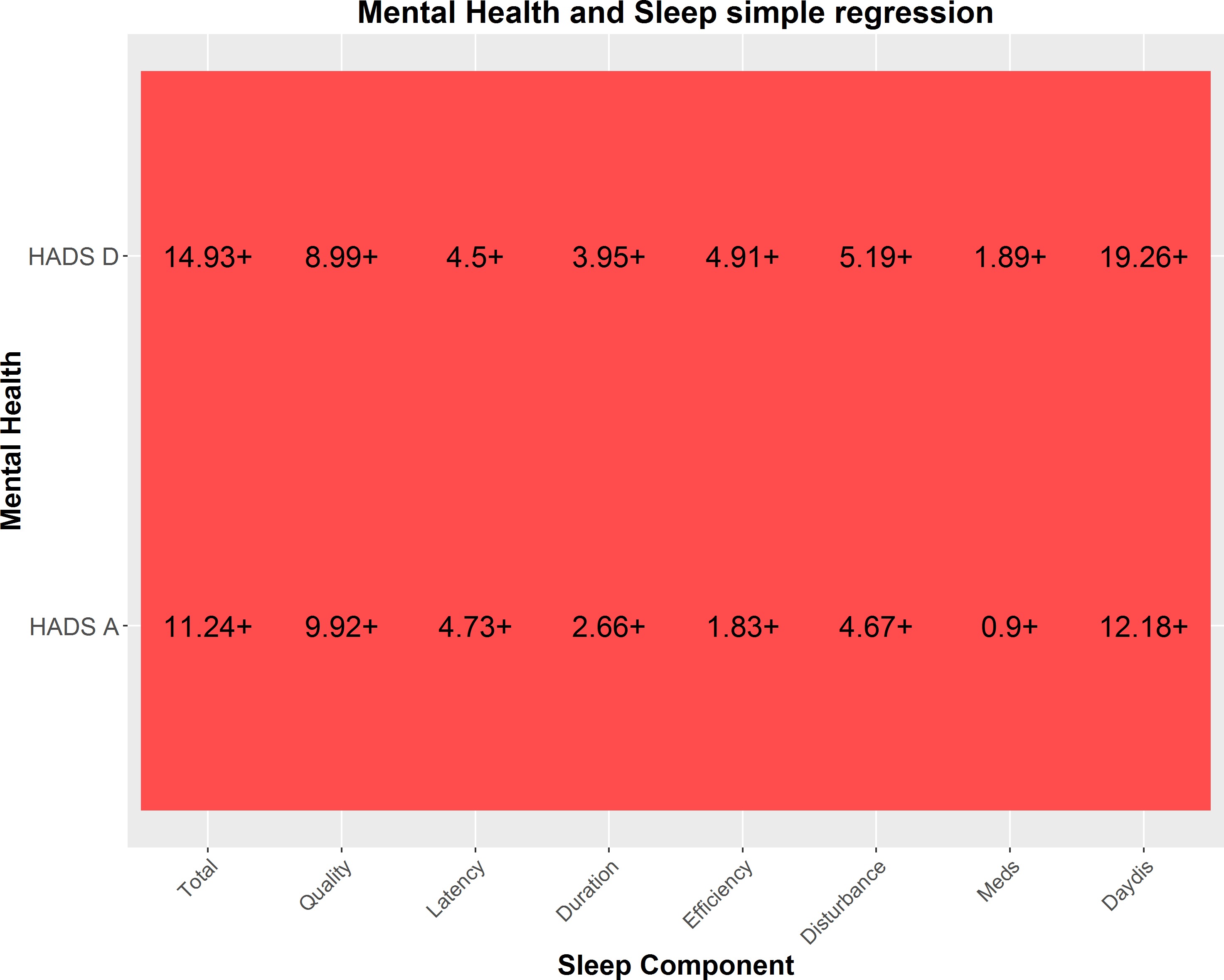

Supplement: Supplementary Figure 10 [file bmjopen-2016-014920supp010.jpg]

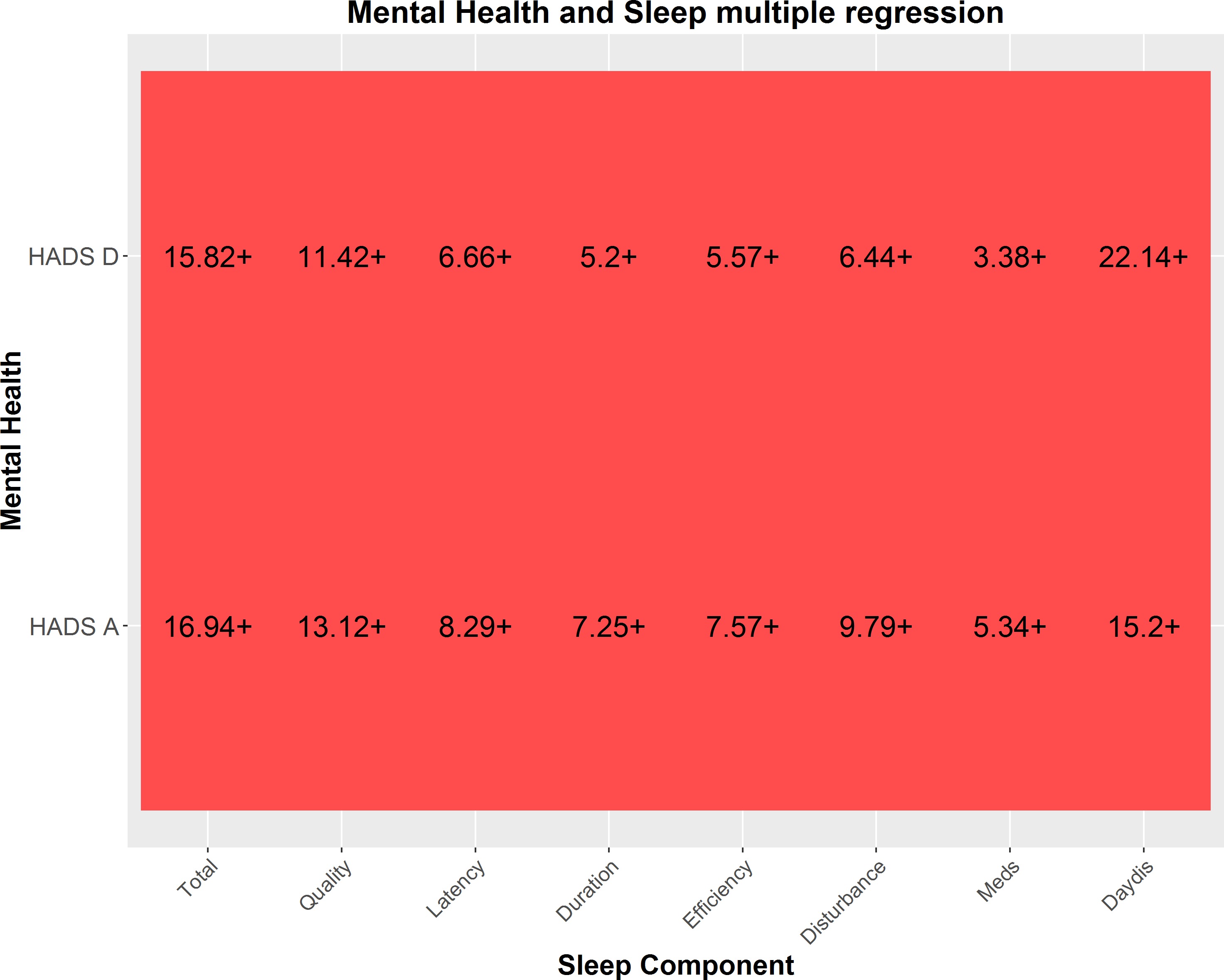

Supplement: Supplementary Figure 11 [file bmjopen-2016-014920supp011.jpg]

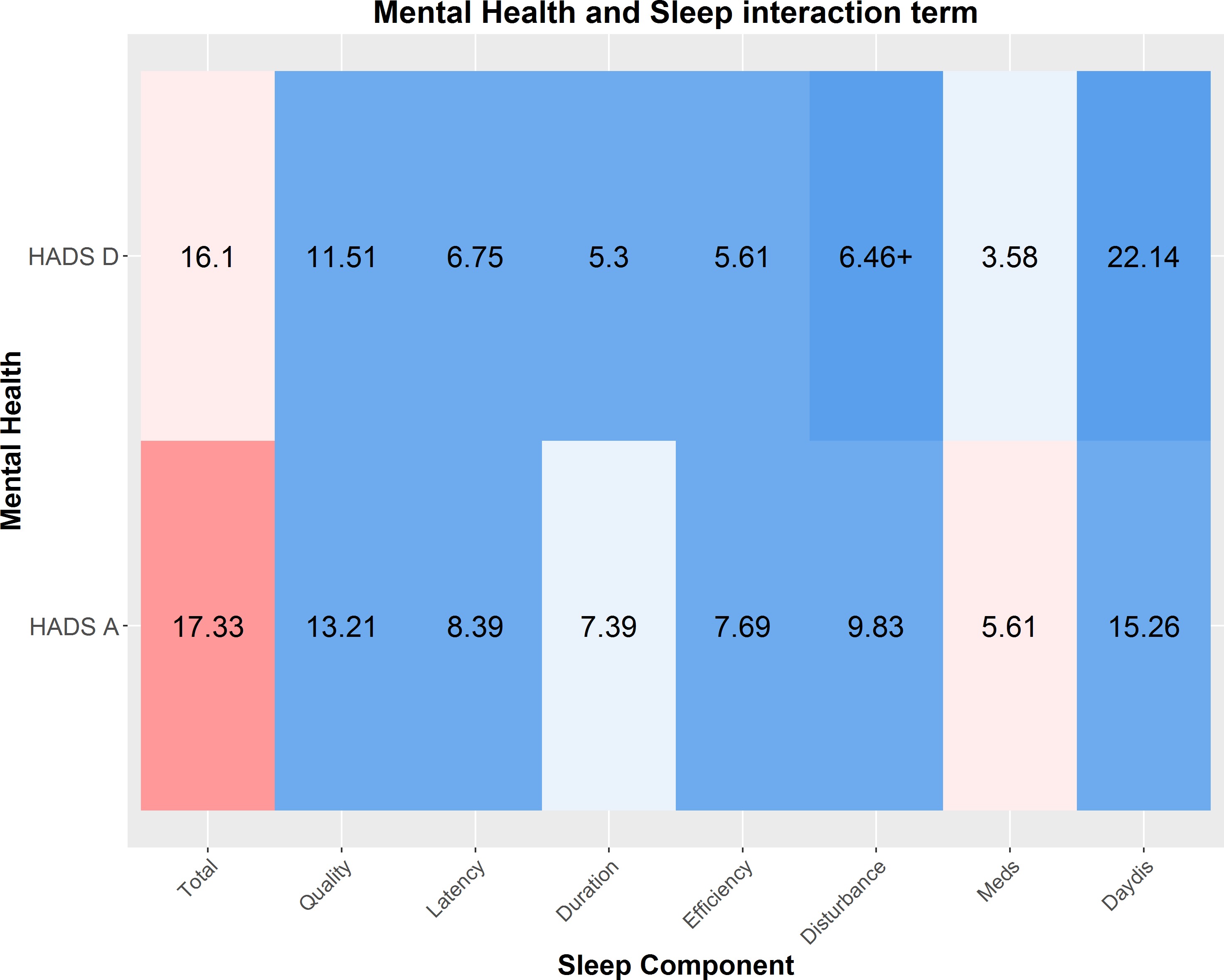

Supplement: Supplementary Figure 12 [file bmjopen-2016-014920supp012.jpg]
