# Supplementary material for: How are age-related differences in sleep quality associated with health outcomes? An epidemiological investigation in a UK cohort of 2406 adults
Source: BMJ Open. 2017 Jul 31;7(7):e014920. doi: 10.1136/bmjopen-2016-014920 (PMC5642766; doi:10.1136/bmjopen-2016-014920)
Supplement: Author's manuscript [file bmjopen-2016-014920.draft_revisions.pdf]

BMJ Open

Age-related differences in self-reported sleep quality predict healthy ageing across multiple domains: a multi-modal cohort of 2406 adults

Journal:	*BMJ Open*
Manuscript ID	bmjopen-2016-014920
Article Type:	Research
Date Submitted by the Author:	26-Oct-2016
Complete List of Authors:	Gadie, Andrew; MRC Cognition and Brain Sciences Unit<br>Shafto, Meredith; University of Cambridge, Center for Speech, Language and the Brain<br>Leng, Yue; University of Cambridge; University of California San Francisco, School of Medicine<br>Cam-CAN, _; University of Cambridge, Center for Sleep, language and the brain<br>Kievit, Rogier; MRC CBSU
**Primary Subject Heading**:	Geriatric medicine
Secondary Subject Heading:	Neurology, Mental health, Epidemiology, Public health
Keywords:	Ageing, SLEEP MEDICINE, cognition, MENTAL HEALTH, Neurobiology < BASIC SCIENCES

SCHOLARONE™
Manuscripts

For peer review only

Age-related differences in self-reported sleep quality predict healthy ageing across multiple domains: a multi-modal cohort of 2406 adults

Andrew Gadie[1]

Meredith Shafto[2]

Yue Leng[3]

Cam-CAN[4]

Rogier A. Kievit[1]*

For peer review only

*Corresponding author: rogier.kievit@mrc-cbu.cam.ac.uk

1 MRC Cognition and Brain Sciences Unit, 15 Chaucer Rd, Cambridge, CB2 7EF, United Kingdom

2 Department of Psychology, University of Cambridge, Downing Street, Cambridge, CB2 3EB, United Kingdom

3 University of California, San Francisco

4 Cambridge Centre for Ageing and Neuroscience (Cam-CAN), University of Cambridge and MRC Cognition and Brain Sciences Unit, Cambridge, UK, www.cam-can.com

For peer review only - http://bmjopen.bmj.com/site/about/guidelines.xhtml

Abstract

Objectives To examine lifespan changes in self-reported sleep quality and their associations with health outcomes across four domains: Physical Health, Cognitive Health, Mental Health and Neural Health.

Setting Cam-CAN is a cohort study in East Anglia/England, which collected self-reported health and lifestyle questions as well as a range of objective measures from healthy adults.

Participants 2406 healthy adults (age 18-98) answered questions about their sleep quality (Pittsburgh Sleep Quality Index) and measures of Physical, Cognitive, Mental, and Neural Health. A subset of 641 individuals provided measures of brain structure.

Main outcome measures Pittsburgh Sleep Quality Index scores (PSQI) of sleep, and scores across tests within the four domains of health. Latent Class Analysis (LCA) is used to identify sleep types across the lifespan. Bayesian regressions quantify the presence, and absence, of relationships between sleep quality and health measures.

Results LCA identified four sleep types: 'Good sleepers' (68.1%, most frequent in middle age), 'inefficient sleepers' (14.01%, most frequent in old age), 'Delayed sleepers' (9.28%, most frequent in young adults) and 'poor sleepers' (8.5%, most frequent in old age). Better sleep is generally associated with better health outcomes, strongly so for mental health, moderately for cognitive and physical health, but not for sleep quality and neural health. There is little evidence for interactions between sleep quality and age on health outcomes.

Conclusions Lifespan changes in sleep quality are multifaceted and not captured well by summary measures, but instead as partially independent symptoms that vary in prevalence across the lifespan. Better self-reported sleep is associated with better health outcomes, and the strength of these associations differs across health domains. Notably, observed absence of associations between sleep quality and white matter suggests that previous associations may depend on clinical samples with pathological sleep deficiencies and may not generalise to healthy cohorts.

For peer review only - http://bmjopen.bmj.com/site/about/guidelines.xhtml

Funding Biotechnology and Biological Sciences Research Council (grant number BB/H008217/1). RAK is supported by the Wellcome Trust (grant number 107392/Z/15/Z and the UK Medical Research Council (MC-A060-5PR61).

Keywords

Ageing, sleep quality, healthy ageing, cognition, mental health, cognition, white matter, physical health

Strengths and limitations of this study

- Broad phenotypic assessment of healthy ageing across multiple health domains
- Advanced analytic techniques (i.e. Latent Class Analysis regression) allows new insights
- Uniquely large neuroimaging sample combined with Bayesian inference allows for quantification of evidence for the null hypothesis
- Subjective sleep measures may have drawbacks in older samples
- Cross-sectional data precludes modelling of within subject changes

For peer review only - http://bmjopen.bmj.com/site/about/guidelines.xhtml

BACKGROUND

Sleep is a fundamental human behaviour, with humans spending almost a third of their lives asleep. Regular and sufficient sleep has been shown to benefit human physiology through a number of different routes, ranging from consolidation of memories (1) to removal of free radicals (2) and neurotoxic waste (3). Sleep patterns are known to change across the lifespan in various ways, including decreases in quantity and quality of sleep (4), changes in the alignment of homeostatic and circadian rhythms (5), decreases in sleep efficiency (6) the amount of slow-wave sleep, and an increase in daytime napping(7). Importantly, interruption and loss of sleep has been shown to have wide ranging adverse effects on health (8), leaving open the possibility that age-related changes in sleep patterns and quality may contribute to well-documented age-related declines in various health domains.

In the current study, we examine self-reported sleep habits in a large, population-based cohort Cambridge Centre for Ageing and Neuroscience (Cam-CAN, (9)). We relate sleep measures to measures of health across four health domains: cognitive, brain health, physical and mental health. Our goal is to quantify and compare the associations between typical age-related changes in sleep quality and a range of measures of health measures that commonly decline in later life. We assess sleep using a self-reported measure of sleep quality, the Pittsburgh Sleep Quality Index (PSQI) (10). The PSQI has good psychometric properties (11) and has been shown to correlate reliably with diseases of aging and mortality (12–14). Although actigraphy (measuring sleep quality in the lab) is commonly considered the gold standard of sleep quality measurement, it is often prohibitively challenging to employ in large samples. A recent direct comparison of sleep measures (15) suggests that although subjective sleep measures (such as PSQI) may have certain drawbacks in older samples, they also capture complementary aspects of sleep quality not fully captured by actigraphy. Moreover, collecting self-report sleep quality data in a large, deeply phenotyped cohort offers several additional benefits.

For peer review only - http://bmjopen.bmj.com/site/about/guidelines.xhtml

First, previous work on the effects of sleep has tended to focus on the pathological extremes of sleep problems (16), leaving open the question whether these findings generalise to how non-pathological differences in sleep quality affect health outcomes in non-clinical samples. Second, smaller studies often focus on specific health outcomes such as metabolism (17) or cognition (18). By instead studying a range of health outcomes in the same population, we can compare and contrast the associations between sleep quality and health domains in multiple domains.

We will focus on three questions within each health domain: First, is there a relationship between sleep quality and health? Second, does the strength and nature of this relationship change when age is included as a covariate? Third, does the strength and nature of the relationship change across the lifespan? We will examine these questions across each of the four health domains.

METHODS

Sample

Participants were recruited as part of the population-based Cambridge Centre for Ageing and Neuroscience (Cam-CAN) cohort (www.cam-can.com). For details of the project protocol see (19) and (20), and for further details of the Cam-CAN dataset visit http://www.mrc-cbu.cam.ac.uk/datasets/camcan/. A further subset participated in a neuroimaging session (20). Participants included were native English speakers, had normal or corrected to normal vision and hearing, and scored 25 or higher on the mini mental state exam (MMSE; Folstein, Folstein, & McHugh, 1975). Ethical approval for the study was obtained from the Cambridgeshire 2 (now East of England- Cambridge Central) Research Ethics Committee (reference: 10/H0308/50). Participants gave written informed consent. The raw data and analysis code are available upon signing a data sharing request form (see http://www.mrc-cbu.cam.ac.uk/datasets/camcan/ for more detail).

For peer review only

For peer review only - http://bmjopen.bmj.com/site/about/guidelines.xhtml

Variables

Sleep Measures

Sleep quality was assessed using the Pittsburgh Sleep Quality Index (PSQI), a well-validated self-report questionnaire (10,15) designed to assist in the diagnosis of sleep disorders. The questions concern sleep patterns, habits, and lifestyle questions, grouped into seven components, each yielding a score ranging from 0 (good sleep/no problems) to 3 (poor sleep/severe problems), that are commonly summed to a PSQI Total score ranging between 0 and 21, with higher scores reflecting poorer sleep quality.

Health Measures

Cognitive health. A number of studies have found associations between poor sleep and cognitive decline, including in elderly populations. Poor sleep affects cognitive abilities such as executive functions (e.g. 22) and learning and memory processes (23), whereas short term pharmaceutical interventions such as administration of melatonin improve both sleep quality and cognitive performance. Scullin & Bliwise (2015, p. 97) conclude that “maintaining good sleep quality, at least in young adulthood and middle age, promotes better cognitive functioning and serves to protect against age-related cognitive declines”. As sleep may affect various aspects of cognition differently (18), we include measures that cover a range of cognitive domains including memory, reasoning, response speed, and verbal fluency, as well as including a measure of general cognition (See Table 1 and (19) for more details).

Neural health. Previous research suggests that individuals with a severe disruption of sleep are significantly more likely to exhibit signs of poor neural health (25,26). Specifically, previous studies have observed decreased white matter health in clinical populations suffering from conditions such as chronic insomnia (16), obstructive sleep apnoea (27,28), excessively long sleep in patients with diabetes (29), and REM Sleep Behaviour Disorder (30). Many of these studies focus on white matter hyperintensities (WMH), a measure of the total volume or number of (regions) showing low-level neural pathology (although some study grey matter, e.g. Altena et al., 2010;

For peer review only - http://bmjopen.bmj.com/site/about/guidelines.xhtml

Macey et al., 2002). White matter hyperintensities are often used as a clinical marker, as longitudinal increases in WMHs are associated with increased risk of stroke, dementia and death (32) and are more prevalent in patients with pathological sleep problems (28,29). However, use of this metric in clinical cohorts largely leaves open the question of the impact of sleep quality on neural (white matter) health in non-clinical, healthy populations. To address this question, we use a more general indicator of white matter neural health; *Fractional Anisotropy* (FA). FA is associated with white matter integrity and myelination (see Mädler, Drabycz, Kolind, Whittall, & MacKay, 2008, for more discussion on the interpretation of FA). We use FA as recent evidence (34) suggests that WMHs represent the extremes (foci) of white matter damage, and that FA is able to capture the full continuum of white matter integrity. For more information regarding the precise white matter pipeline, see (35)

Physical health. Sleep quality is also an important marker for physical health, with poorer sleep being associated with conditions such as obesity, diabetes mellitus (17), overall health (8,36) and increased all-cause mortality (37,38). We focus on a set of variables that capture three types of health domains commonly associated with poor sleep: Cardiovascular health measured by pulse, systolic and diastolic blood pressure (39), self-reported health, both in general and for the past 12 months, (e.g. Strine & Chapman, 2005) and body-mass index (e.g. Taheri, Lin, Austin, Young, & Mignot, 2004).

Mental health. Previous work has found that disruptions of sleep quality are a central symptom of forms of psychopathology such as Major Depressive Disorder, including both hypersomnia and insomnia (36,42), and episodes of insomnia earlier greatly increased the risk of later episodes of major depression (43). Kaneita et al., (2006) found a U-shaped association between sleep and depression, such that individuals regularly sleeping less than 6, or more than 8, hours were more likely to be depressed. Both depression (e.g. Fried & Nesse, 2015) and anxiety (46,47) are commonly associated with sleep problems. To capture these dimensions we used both scales of the

For peer review only - http://bmjopen.bmj.com/site/about/guidelines.xhtml

Hospital Anxiety and Depression Scale (HADS) (48), a widely used and standardized questionnaire that captures self-reported frequency and intensity of anxiety and depression symptoms.

For peer review only

For peer review only - http://bmjopen.bmj.com/site/about/guidelines.xhtml

Table 1. Description of health variables across each of four domains (cognitive, neural, physical, mental). For each variable details are given including a description of the task it is derived from, relevant citations, a brief definition and descriptive statistics.

Health domain	Task and Description	Variable	Descriptives	Citation
Cognitive	Story Recall Immediate: Participants hear a short story and are asked to recall as accurately as possible.	Recall manually scored for similarity and precision (min=0, max=24)	M=13.14, SD=4.66, Range=(0-24)	(49)
Cognitive	Story Recall Delayed: Same as above but recall after 30 minute delay	Recall manually scored for similarity and precision (min=0, max=24)	M=11.47, SD=4.92, Range=(0-24)	(49)
Cognitive	Letter Fluency (phonemic fluency): Participants have one minute to generate as many words as possible beginning with the letter 'p'.	Total words generated (min=0,max=30)	M=25.38, SD=3.96, Range=(0-30)	(49)
Cognitive	Animal Fluency (semantic fluency): Participants have one minute to generate as many words as possible in the category 'animals'.	Total words generated (min=0,max=30)	M=25.85, SD=4.47, Range=(0-30)	(49)
Cognitive	Cattell Culture Fair: Test of fluid reasoning using four subtests (series completions, odd-one-out, matrices and topology)	Total correct summed across four subtests. Min=0, max=46	M=31.8, SD=6.79, Range=(11-44)	(50)
Cognitive	Simple reaction time: Speed in a simple reaction time task	1/response time in seconds	M=0.37, SD=0.08, Range=(0.24-0.93)	(9)
Cognitive	Addenbrookes Cognitive Examination, Revised: Screening test for dementia using seven subtests (orientation, attention and concentration, memory, fluency, language, visuospatial abilities, perceptual abilities)	Performance on multiple tests converted to min=0, max=100 range	M=89.25, SD=13.4, Range=(0-100)	(51)
Neural	White matter health: Measure of tract integrity using fractional anisotropy	Fractional Anisotropy (min=0, max=1, averaged across 10 tracts)	M=0.5, SD=0.03, Range=(0.3-0.56)	(52)
Physical	Self-reported Health, in general: Participants use a 4-point scale to respond to the prompt "Would you say for someone of your age, your own health in	Score from 1 = Excellent to 4= Poor	M=2.02, SD=0.79, Range=(1-3)	(53)

For peer review only - http://bmjopen.bmj.com/site/about/guidelines.xhtml

	general is..."			
Physical	Self-reported Health, last 12 months:<br>Participants use a 3-point scale to respond to the prompt "Over the last twelve months would you say your health has on the whole been..."	Score from 1 = Good to 3= Poor	M=1.46, SD=0.71, Range=(1-3)	(53)
Physical	Systolic blood pressure	Mean systolic blood pressure in mmHg, averaged across three consecutive measurements	M=120.11, SD=17, Range=(78.5-186)	
Physical	Diastolic blood pressure	Mean diastolic blood pressure in mmHg, averaged across three consecutive measurements	M=73.14, SD=10.48, Range=(49-115.5)	
Physical	Resting pulse	Mean pulse in beats per minute, averaged across three consecutive measurements	M=65.69, SD=10.5, Range=(40-110.5)	
Physical	Body Mass Index (BMI)	(weight in kg) / (height in m)^2	M=25.77, SD=4.59, Range=(16.75-48.32)	(54)
Mental health	Anxiety Subscale (Hospital Anxiety and Depression Scale (HADS)):<br>Participants response to seven questions about anxiety-related behaviours	Seven questions rated on 0 to 3 scale ('Often' to 'Very seldom'). Min=0, Max=21	M=5.17, SD=3.4, Range=(0-19)	(48)
Mental health	Depression Subscale (Hospital Anxiety and Depression Scale (HADS)):<br>Participants response to seven questions about depression-related behaviours	Seven questions rated on 0 to 3 scale ('Often' to 'Very seldom'). Min=0, Max=21	M=3.32, SD=2.91, Range=(0-14)	

For peer review only

For peer review only - http://bmjopen.bmj.com/site/about/guidelines.xhtml

STATISTICAL ANALYSES

We examine whether self-reported sleep patterns change across the lifespan, both for the PSQI sum score and for each of the seven PSQI components. We then examine the relationships between the sleep quality and the four health domains in three ways: First, simple regression of the health outcome on sleep variables, to determine evidence for association between poor sleep quality and poor health outcomes. Second, we include age as a covariate. Finally, we include a (standard normal rescaled) continuous interaction term to examine whether there is evidence for a changing relationship between sleep and outcomes across the lifespan.

For all regressions we will use a default Bayesian approach advocated by Liang, Paulo, Molina, Clyde, & Berger, (2008); Rouder & Morey, (2012); Wagenmakers, (2007); Wei et al., (2012); Wetzels et al., (2011), which avoids several well-documented issues with p-values (57), allows for quantification of null effects, and decreases the risk of multiple comparison problems (e.g. Gelman, Hill, & Yajima, 2012). Bayesian regressions allows us to symmetrically quantify evidence in favour of, or against, some substantive model as compared to a baseline (e.g. null) model. This evidentiary strength is expressed as a Bayes Factor (see Jeffreys (61), which can be interpreted as the relative likelihood of one model versus another given the data and a certain prior expectation. A Bayes Factor of, e.g., 7, in favour of a regression model suggests that the data are seven times *more likely* under that model than an intercept only model (for an empirical comparison of p-values and Bayes factors, see Wetzels et al., 2011). A heuristic summary of evidentiary interpretation can be seen in Figure 1.

[insert Figure 1 here]

We report log Bayes Factors for large effects and regular Bayes Factors for smaller effects. To compute Bayes Factors we will use Default Bayes Factor approach for model selection (55,56) in the package BayesFactor (62) using the open source software package R (63). As previous papers report associations between sleep and outcomes ranging from absent to considerable in size we utilize the default, symmetric Cauchy prior with width $\frac{\sqrt{2}}{2}$ which translates to a 50% confidence that

For peer review only - http://bmjopen.bmj.com/site/about/guidelines.xhtml

the true effect will lie between -.707 and .707. Prior to further analysis, scores on all outcomes were transformed to a standard normal distribution, and any scores exceeding a z-score of 4 or -4 were recoded as missing (aggregate percentage outliers across the four health domains: Cognitive, 0.41%, Mental, 0.16%, Neural, 0.37% Physical, 0.031%).

To better elucidate individual differences in sleep quality we next use *Latent Class Analysis* (64). This technique will allow us examine individual differences in sleep quality across the lifespan in more detail than afforded by simple linear regressions: Rather than examining continuous variation in sleep components, LCA classifies individuals into different *sleep types*, each associated with a distinct profile of 'sleep symptoms'. If there are specific constellations of sleep problems across individuals, we can quantify and visualize such sleep types. Moreover, by using Latent Class Regression, we can examine whether the likelihood of belonging to any sleep 'type' changes as a function of age. To analyse the data in this manner, we binarized the responses on each component into 'good' (0 or 1) or 'poor' (2 or 3).

RESULTS

Age-related differences in sleep quality

First, we examined sleep changes across the lifespan by examining age-related differences in the PSQI sum score (N= 2178, M=5.16, SD=3.35, Range=0-19). Regressing the PSQI global score on age, (see Supplementary Figure 1) showed evidence for a positive relationship across the lifespan ($logBF_{10}$= 10.45). This suggests that on the whole, sleep quality decreases across the lifespan (note that *higher* PSQI scores correspond to worse sleep). Although we observe strong statistical evidence for an age-related difference ('Extreme' according to Jeffreys, (1961)), age explained only 1.23 % of the variance in the PSQI Total score. Next, we examined each of the seven components on age in the same manner. In Supplementary Figure 2 we see that that age has varying and specific effects on different aspects of sleep quality, and did not worsen uniformly across the lifespan. For example, we observed moderate evidence that sleep latency did not change across the lifespan (Sleep Latency,

For peer review only - http://bmjopen.bmj.com/site/about/guidelines.xhtml

BF_{01}= 9.25, in favour of the null), Sleep Quality showed no evidence for either change or stasis (BF_{10}= 1.63) and one sleep component, Daytime Dysfunction, improved slightly across the lifespan (BF_{10}= 7.03). Medication). The strongest age-related decline is that of Efficiency, showing an R-squared of 6.6%.

Finally, we entered all seven components into a Bayesian multiple regression simultaneously, to examine to what extent they could, together, predict age. The best model included every component except Sleep Latency (logBF_{10}= 142.71). Interestingly, this model explained 13.41% of the variance in age, compared to 1.23% for the PSQI Total score, and 6.4% for the strongest single component. This shows that lifespan changes in self-reported sleep are heterogeneous and partially independent, and that specific patterns and components need to be taken into account simultaneously to fully understand age-related differences in sleep quality. These finding shows that neither the PSQI sum score nor the sleep components in isolation fully capture differences in sleep quality across the lifespan.

Next we examined evidence for distinct sleep types using Latent Class Analysis (64). We fit a set of possible models (varying from 2 to 6 sleep types) We found that the four class solution gives the best solution, according to the Bayesian Information Criterion (65) (BIC for 4 Classes = 11825.65, lowest BIC for other solutions= 11884.92 (5 classes) (with 50 repetitions per class, at 5000 maximum iterations). Next we inspected the nature of the sleep types, the prevalence of each 'sleep type' in the population, and whether the likelihood of belonging to a certain sleep type changes across the lifespan. See Figure 2 for the component profiles of the four sleep types identified.

[insert Figure 2 here]

Class 1, 'Good sleepers', make up 68.1% of participants. Their sleep profile is shown in Figure 2A, top left, and is characterised by a low probability of responding 'poor' to any of the sleep components. Class 2, 'inefficient sleepers', make up 14.01% of the participants, and are characterized by poor sleep Efficiency: Members of this group uniformly (100%) report poor sleep Efficiency, despite relatively low prevalence of other sleep problems, as seen in Figure 2A, top right.

For peer review only - http://bmjopen.bmj.com/site/about/guidelines.xhtml

Class 3, 'Delayed Sleepers' seen in the bottom left of Figure 2a, makes up 9.28% of the participants: characterized by modestly poor sleep across the board, but a relatively high probability of poor scores on Sleep Latency (59%), Sleep Quality (51%) and sleep Disturbance (31%). Finally, Class 4, 'Poor sleepers', make up 8.5% of the participants, shown bottom right in Figure 2A. Their responses to any of the seven sleep components are likely to be 'poor' or 'very poor', almost universally so for 'sleep quality' (94%) and 'Sleep Efficiency' (97.7%).

Next, we including age as a covariate (simultaneously including a covariate is known as *latent class regression* or concomitant-variable latent class models (66). This analysis, visualised in Figure 2b, shows that the probability of membership of each classes compared to the reference class (good sleepers) changes significantly across the lifespan for each of the classes (Class 2 versus class 1: beta/SE= 0.05/0.00681, t=7.611, Class 3 versus class 1: beta/SE= -0.01948/0.0055, t=-3.54), Class 4 versus class 1: beta/SE 0.01269/0.00478, t=2.655, for more details on generalized logit coefficients , see Linzer & Lewis, 2011, p. 21). The frequency of Class 1 (Good sleepers) peaks in middle to late adulthood, dropping increasingly quickly after age 50. Class 2 (Inefficient sleepers) are relatively rare in younger individuals, but the prevalence increases rapidly in individuals over age 50. On the other hand, Class 3 (Delayed sleepers) shows a steady decrease in the probability of an individual showing this profile across the lifespan, suggesting that this specific pattern of poor sleep is more commonly associated with younger adults. Finally, the proportion of Class 4 (poor sleepers) members increases only slightly across the lifespan. Together, the latent class analysis provides additional evidence that the PSQI sum score as an indicator of sleep quality does not fully capture the subtleties of age-related differences. Age-related changes in sleep patterns are characterized by specific, clustered patterns of sleep problems that cannot be adequately characterized by summation of the component scores. The above analyses show how both a summary measure and individual measures of sleep quality change across the lifespan. Next, we examined the relationships between sleep quality measures (seven components and the global PSQI score) and health variables (specific variables across four domains, as shown in Table 1).

For peer review only - http://bmjopen.bmj.com/site/about/guidelines.xhtml

For peer review only

Sleep, health domains and age

Cognitive health

First, we examined the relationships between sleep quality and seven measures of cognitive health (see Table 1 for details). As can be seen in Figure 3, several relationships exist between measures of cognitive health and measures of sleep quality. We visualise these results using a tile plot (68), as shown in Figure 3.

[Insert Figure 3 here]

Each cell shows the numeric effect size (R-squared, 0-100) of the bivariate association between a sleep component and a health outcome, colour coded by the statistical evidence for a relationship using the Bayes Factor. If the parameter estimate is positive, the r-squared value has the symbol '+' added (note the interpretation depends on the nature of the variable, cf. Table 1). The strongest associations were found for poorer Total Sleep, poorer sleep Efficiency and use of Sleep Medication, all associated with poorer performance on cognitive tests. The cognitive abilities most strongly associated with poor sleep are immediate and delayed memory, fluid reasoning and a measure of general cognitive health, ACE-R. Two patterns emerged: First, the strongest predictor across the simple and multiple regressions was for the PSQI Total score. Tentatively this suggests that a cumulative index of sleep problems, rather than any specific pattern of poor sleep, is the biggest risk factor for poorer cognitive performance. Secondly, after controlling for age, the most strongly affected cognitive measure is phonemic fluency, the ability to generate name as many different words as possible starting with a given letter within a minute. Verbal fluency is commonly used as a neuropsychological test (e.g. Miller, 1984). Previous work suggests it depends on both the ability to cluster (generating words within a semantic cluster) and to switch (switching between categories), and is especially vulnerable to frontal lobe damage Although modest in size, our findings suggests this task, dependent on multiple executive processes, is particularly affected by poor sleep quality (70). The second strongest association was with the ACE-R, a general cognitive test battery

For peer review only - http://bmjopen.bmj.com/site/about/guidelines.xhtml

similar in style and content to the MMSE. The associations with cognition were slightly attenuated when age was included as a covariate (Supplementary Figure 3) but the basic effects remained.

When an interaction term with age was included, no evidence for interactions with age were observed (mean $logBF_{10}$=-2.08, see Supplementary Figure 4), suggesting that the negative associations between sleep and cognitive performance are a constant feature across the lifespan, rather than specifically in elderly individuals. Together this suggests that poor sleep quality is modestly and consistently associated with poorer general cognitive performance across the lifespan, most strongly with semantic fluency.

Neural Health

Using Diffusion Tensor Imaging, we estimated a general index of white matter integrity in 10 tracts (52) (shown in Supplementary Figure 5), by taking the average Fractional Anisotropy in each white matter ROI (see (71) for more information). We use the data from a subsample of 641 individuals (age M=54.87, range 18.48-88.96) who were scanned in a 3T MRI scanner (for more details regarding the pipeline, sequence and processing steps, see (71)). Regressing neural WM ROI's on sleep quality, we find several small effects, with the strongest associations between sleep efficiency and neural health (see Supplementary Figure 6). All effects are such that poorer sleep is associated with poorer neural health, apart from a small effect in the opposite direction for Uncinate and Daytime Dysfunction (BF_{10}= 6.20). However, when age is included as a covariate, the negative associations between sleep quality and white matter health are attenuated virtually to zero (Figure 4, mean/median BF_{10}= 0.18/.10), with Bayes Factors providing strong evidence for the lack of associations between sleep quality and white matter integrity. One exception was observed: The use of Sleep Medication is associated with *better* neural health in the corticospinal tract, a region previously found to be affected by pathological sleep problems such as sleep apnoea (28). However, this effect is very small (BF_{10}=3.24) given the magnitude of the sample and the range of comparisons, so should be interpreted with caution.

For peer review only - http://bmjopen.bmj.com/site/about/guidelines.xhtml

[Insert Figure 4 here]

Finally, we tested for any interactions by including a mean-scaled interaction term (sleep*age, Supplementary Figure 7). This analysis found evidence for a significant interaction, between the Superior Longitudinal Fasciculus (SLF) and Sleep Medication (BF_{10}= 13.77), such that better neural health in the SLF was associated with the use of Sleep Medication more strongly in older adults. Together, these findings suggest that in general, once age is taken into account, self-reported sleep problems in a non-clinical sample are *not* associated with poorer neural health, although there is some evidence for a modest associations between better neural health in specific tracts and the use of sleep medication in the elderly.

Physical health

Next we examined whether sleep quality is associated with physical health. Figure 5 shows the simple regressions between sleep quality and physical health. Strong associations were found between poor overall sleep (PSQI sum score) and poor self-reported health, both in general ($logBF_{10}$=77.51) and even more strongly for health in the past 12 months ($logBF_{10}$=91.25). This may be because poorer sleep, across all components, directly affects general physical health (Briones et al., 1996; Spiegel et al., 2009) or because people subjectively experience sleep quality as a fundamental part of overall general health. A second association was between BMI and poor sleep quality, most strongly poor Duration ($logBF_{10}$=4.69).

[Insert Figure 5 here]

This not only replicates previous findings but is in line with an increasing body of evidence that suggests that shorted sleep duration causes metabolic changes, which in turn increases the risk of both diabetes mellitus and obesity (17,73,74). Next, we examined whether these effects were attenuated once age was included. We show that although the relationships are slightly weaker, the overall pattern remains (Supplementary Figure 8), suggesting these associations are not merely co-

For peer review only - http://bmjopen.bmj.com/site/about/guidelines.xhtml

occurences across the lifespan. Our findings suggest self-reported sleep quality, especially sleep Duration, is related to differences in physical health outcomes in a healthy sample.

Finally, there was evidence of a single interaction with age (Supplementary Figure 9): Although poor sleep Duration was associated with *higher* diastolic blood pressure in younger adults, it was associated with *lower* diastolic blood pressure in older individuals (BF_{10}= 8.53). This may reflect the fact that diastolic blood pressure is related to cardiovascular health in a different way across the lifespan, although given the small effect size it should be interpreted with caution.

Mental health

Finally, we examined the relationship between sleep quality and mental health, as measured by the Hospital Anxiety and Depression Scale (48). One benefit of the HADS in this context is that, unlike some other definitions (e.g. the DSM-V), sleep quality is not an integral (scored) symptom of these dimensions. As shown in Supplementary Figure 10, there are very strong relationships between all aspects of sleep quality and measures of both anxiety and depression. The strongest predictors of Depression are Daytime Dysfunction ($logBF_{10}$= 245.9, R^2=20.9%), followed by the overall sleep score ($logBF_{10}$= 170.5, R^2=14.6%) and sleep quality ($logBF_{10}$= 106.8, R^2=9.7%). The effects size for Anxiety was comparable but slightly smaller in magnitude. When age is included as a covariate the relationships remained virtually unchanged (Supplementary Figure 11), suggesting these relationships are present throughout across the lifespan. These findings replicate and extend previous work, suggesting that sleep quality is strongly associated with both anxiety and depression across the lifespan.

Finally we examined a model with an interaction term (Supplementary Figure 12). Most prominently we found interactions with age in the relationship between HADS depression and the PSQI Total, and in the relationship between HADS depression and Sleep Duration, such that for the relationship between anxiety and overall sleep quality is stronger in younger adults (BF_{10} =9.91, see

For peer review only - http://bmjopen.bmj.com/site/about/guidelines.xhtml

Figure 6). Together our findings show that poor sleep quality is consistently, strongly and stably associated with poorer mental health across the adult lifespan.

[Insert Figure 6 here]

DISCUSSION

In this study, we report on the associations between age-related differences in sleep quality and health outcomes in a large, age-heterogeneous sample of community dwelling adults of the Cambridge Neuroscience and Aging (Cam-CAN) cohort. We find that sleep quality generally decreases across the lifespan, most strongly for sleep Efficiency. However age-related changes in sleep patterns are complex and multifaceted, so we used Latent Class Analysis to identify 'sleep types' associated with specific sleep quality profiles. We found that Younger adults are more likely than older adults to display a pattern of sleep problems characterised by poor sleep quality and longer sleep latency, whereas older adults are more likely to display inefficient sleeping, characterised by long periods spent in bed whilst not asleep. Moreover, the probability of being a 'good' sleeper, unaffected by any adverse sleep symptoms, decreases considerably after age fifty.

Our broad phenotypic assessment allows for direct comparison of the different measures of sleep quality and four key health domains We find strongest associations between sleep quality and mental health, moderate relations between sleep quality and physical health and cognitive health and sleep, virtually all such that poorer sleep is associated with poorer health outcomes. We did not find evidence for associations between self-reported sleep and neural health. Notably, the relationships we observe are mostly stable across the lifespan, affecting younger and older individuals alike. A notable exception to these effects is the absence of any strong relation (after controlling for age) between sleep quality and neural health as indexed by tract-based average fractional anisotropy. Using a Bayesian framework we observed evidence in favour of the null hypothesis, suggesting that the adverse effects of poor sleep on brain structure found in more extreme clinical samples (e.g. insomnia, sleep apnoea) do not necessarily generalize to a non-clinical

For peer review only - http://bmjopen.bmj.com/site/about/guidelines.xhtml

population for self-reported sleep. Notably, as we found strong relationships in the same sample between sleep and other outcomes (e.g. mental health, Figure 10) and there is previous evidence from this cohort linking white matter health and cognition, the absence of the relationship between poor sleep and neural health cannot be (fully) explained away by the possible noisiness of self-report measures or white matter measures. For this reason, our study provides a potentially reassuring message that for typically-ageing, healthy individuals, poorer self-reported sleep quality is not associated with poorer brain health.

While there are limitations of self-report measures including in older cohorts (15), including the fact that they likely reflect different aspects of sleep health than actigraphy (sleep in the lab), our results suggest there are considerable advantages in using self-reported sleep measures: first, obtaining sleep quality data in a large and broadly phenotyped sample is feasible; and second, our results demonstrated clear and consistent associations across multiple domains for both subjective (e.g. self-reported health) and objective measures (e.g. memory tests, BMI), which both replicate and extend previous lab-based sleep findings. Future work should ideally simultaneously measure actigraphy and self-report in large scale cohorts to fully capture the range of overlapping and complementary relations between different aspects of sleep quality and health outcomes (15).

For both self-report and objective measures of sleep quality an open question is that of causality: Does poor sleep affect health outcomes, do health problems affect sleep, are they both markers of some third problem, or do causal influences go both ways? Most likely, all these patterns occur to varying degrees. Previous studies have shown that sleep quality causally affects health outcomes such as diabetes (17) and memory consolidation (1) while other evidence suggests that depression directly affect sleep quality (Lustberg & Reynolds, 2000; Sbarra & Allen, 2009) and that damage to neural structures may affect sleep regulation (77). Although our findings are in keeping with previous findings, our cross-sectional sample cannot tease apart the causal direction of the observed associations, more work remains to be done to disentangle these complex causal pathways.

For peer review only - http://bmjopen.bmj.com/site/about/guidelines.xhtml

In our paper we focus on a healthy, age-heterogeneous community dwelling sample. This allows us to study the associations between healthy aging and self-reported sleep quality, but comes with two key limitations of the interpretations of our findings. First and foremost, our findings are cross-sectional, not longitudinal. This means we can make inferences about age-related *differences*, but not necessarily age-related *changes* (Raz & Lindenberger, 2011; Schaie, 1994). One reason why cross-sectional and longitudinal estimates may diverge is that older adults can be thought of as cohorts that differ from the younger adults in more ways than age alone. For example, our age range includes individuals born in the twenties and thirties of the 20th century. Compared to someone born in the 21st century, these individuals will likely have experience various differences during early life development (e.g. less broadly accessible education, lower quality of healthcare, poorer nutrition and similar patterns). For some of our measures, these are inherent limitations –*truly* longitudinal study of neural aging is inherently impossible as scanner technology has not been around sufficiently long. This means our findings likely reflect a combination of effects attributable to age-related changes as well as baseline differences between subpopulations that may affect both mean differences as well as developmental trajectories.

Second, our sample reflects an atypical population in the sense that they are willing and able to visit the laboratory on multiple occasions for testing sessions. This subsample is likely a more healthy subset of the full population, which will mean the range of (poor) sleep quality as well as (poorer) health outcomes will likely be less extreme that in the full population. However, this challenge is not specific to our sample. In fact, as the Cam-CAN cohort was developed using stratified sampling based on primary healthcare providers, our sample is likely as population-representative as is feasible for a cohort of this magnitude and phenotypic breadth (see Shafto et al., 2014b) for further details). Nonetheless, a healthier subsample may lead to restriction of range (80), i.e. an attenuation of the strength of the associations observed between sleep quality and health outcomes. Practically, this means that our results likely generalise to comparable, healthy

For peer review only - http://bmjopen.bmj.com/site/about/guidelines.xhtml

community dwelling adults, but not necessarily to populations that include those affected by either clinical sleep deprivation or other serious health conditions.

Conclusions

Taken together, our study allows several conclusions. First, although we replicate the age-related deterioration in some aspects of sleep quality, other aspects remain stable or even improve. Second, we show that the profile of sleep quality changes across the lifespan. This is important methodologically, as it suggests that PSQI sum scores do not capture the full picture, especially in age-heterogeneous samples. Moreover, it is important from a psychological standpoint: We show that 'sleep quality' is a multidimensional construct and should be treated as such if we wish to understand the complex effects and consequences of sleep quality across the lifespan. Third, moderate to strong relations exist between sleep quality and cognitive, physical and mental health, and these relations largely remain stable across the lifespan. In contrast, we show evidence that in non-clinical populations, poorer self-reported sleep is not reliably associated with poorer neural health. Together with previous experimental and longitudinal evidence, our findings suggest that at least some age-related decreases in health outcomes may be due to poorer sleep quality. We show that self-reported sleep quality can be an important indicator of other aspects of healthy functioning throughout the lifespan, especially for mental and general physical health. Our findings suggest accurate understanding of sleep quality is essential in understanding and supporting healthy aging across the lifespan.

For peer review only - http://bmjopen.bmj.com/site/about/guidelines.xhtml

Author contributions

AG, MS and MS designed the study. AG and RAK performed the analyses. CC organized and conducted the data collection. AG, MS and RAK wrote the manuscript. YL provided considerable expertise on sleep and poor sleep outcomes. All authors approved the final manuscript.

Acknowledgements

The Cambridge Centre for Ageing and Neuroscience (Cam-CAN) research was supported by the Biotechnology and Biological Sciences Research Council (grant number BB/H008217/1). RAK is supported by the Sir Henry Wellcome Trust (grant number 107392/Z/15/Z) and the by UK Medical Research Council Programme (MC-A060-5PR61). We would like to thank Richard Morey and Eric-Jan Wagenmakers for valuable suggestions regarding the use of the BayesFactor package. We are grateful to the Cam-CAN respondents and their primary care teams in Cambridge for their participation in this study. We also thank colleagues at the MRC Cognition and Brain Sciences Unit MEG and MRI facilities for their assistance. The Cam-CAN corporate author consists of the project principal personnel: Lorraine K Tyler, Carol Brayne, Edward T Bullmore, Andrew C Calder, Rhodri Cusack, Tim Dalgleish, John Duncan, Richard N Henson, Fiona E Matthews, William D Marslen-Wilson, James B Rowe, Research Associates: Karen Campbell, Teresa Cheung, Simon Davis, Linda Geerligs, Anna McCarrey, Abdur Mustafa, Darren Price, David Samu, Jason R Taylor, Matthias Treder, Kamen Tsvetanov, Janna van Belle, Nitin Williams; Research Assistants: Lauren Bates, Tina Emery, Sharon Erzinçlioglu, Sofia Gerbase, Stanimira Georgieva, Claire Hanley, Beth Parkin, David Troy; Affiliated Personnel: Tibor Auer, Marta Correia, Lu Gao, Emma Green, Rafael Henriques; Research Interviewers: Jodie Allen, Gillian Amery, Liana Amunts, Anne Barcroft, Amanda Castle, Cheryl Dias, Jonathan Dowrick, Melissa Fair, Hayley Fisher, Anna Goulding, Adarsh Grewal, Geoff Hale, Andrew Hilton, Frances Johnson, Patricia Johnston, Thea Kavanagh-Williamson, Magdalena Kwasniewska, Alison McMinn, Kim Norman, Jessica Penrose, Fiona Roby, Diane Rowland, John Sargeant, Maggie

For peer review only - http://bmjopen.bmj.com/site/about/guidelines.xhtml

Squire, Beth Stevens, Aldabra Stoddart, Cheryl Stone, Tracy Thompson, Ozlem Yazlik; and administrative staff: Dan Barnes, Marie Dixon, Jaya Hillman, Joanne Mitchell, Laura Villis.

For peer review only

For peer review only - http://bmjopen.bmj.com/site/about/guidelines.xhtml

References

1. Stickgold R. Sleep-dependent memory consolidation. Nature [Internet]. 2005 Oct 27 [cited 2014 Jul 10];437(7063):1272–8. Available from: http://dx.doi.org/10.1038/nature04286

2. Inoué S, Honda K, Komoda Y. Sleep as neuronal detoxification and restitution. Behav Brain Res. 1995 Jul;69(1–2):91–6.

3. Xie L, Kang H, Xu Q, Chen MJ, Liao Y, Thiyagarajan M, et al. Sleep drives metabolite clearance from the adult brain. Science [Internet]. NIH Public Access; 2013 Oct 18 [cited 2014 Jul 11];342(6156):373–7. Available from: http://europepmc.org/articles/PMC3880190/?report=abstract

4. D'Ambrosio C, Redline S. Impact of Sleep and Sleep Disturbances on Obesity and Cancer. Redline S, Berger NA, editors. New York, NY: Springer New York; 2014.

5. Schmidt C, Peigneux P, Cajochen C. Age-related changes in sleep and circadian rhythms: impact on cognitive performance and underlying neuroanatomical networks. Front Neurol [Internet]. 2012 Jan [cited 2014 Jun 4];3:118. Available from: http://www.pubmedcentral.nih.gov/articlerender.fcgi?artid=3405459&tool=pmcentrez&rendertype=abstract

6. Leng Y, Wainwright NWJ, Cappuccio FP, Surtees PG, Luben R, Wareham N, et al. Self-reported sleep patterns in a British population cohort. Sleep Med [Internet]. 2014 Mar [cited 2016 Jan 28];15(3):295–302. Available from: http://www.sciencedirect.com/science/article/pii/S1389945714000185

7. Stanley N. The physiology of sleep and the impact of ageing. Eur Urol Suppl [Internet]. 2005 Jan [cited 2014 Sep 23];3(6):17–23. Available from: http://www.sciencedirect.com/science/article/pii/S156990560580003X

8. Briones B, Adams N, Strauss M, Rosenberg C, et al. Relationship between sleepiness and general health status. Sleep. 1996;19(7):583–8.

9. Shafto MA, Tyler LK, Dixon M, Taylor JR, Rowe JB, Cusack R, et al. The Cambridge Centre for Ageing and Neuroscience (Cam-CAN) study protocol: a cross-sectional, lifespan, multidisciplinary examination of healthy cognitive ageing. BMC Neurol [Internet]. BioMed Central; 2014 Jan 14 [cited 2015 May 20];14(1):204. Available from: http://bmcneurol.biomedcentral.com/articles/10.1186/s12883-014-0204-1

10. Buysse D, Reynolds C, Monk T, Berman S, Kupfer D. The Pittsburgh Sleep Quality Index: A new instrument for Psychiatric Practise and Research .pdf. 1988. p. 193–213.

11. Carpenter JS, Andrykowski MA. Psychometric evaluation of the pittsburgh sleep quality index. J Psychosom Res [Internet]. 1998 Jul [cited 2015 Dec 10];45(1):5–13. Available from: http://www.sciencedirect.com/science/article/pii/S0022399997002985

12. Kang S-H, Yoon I-Y, Lee SD, Kim J-W. The impact of sleep apnoea syndrome on nocturia according to age in men. BJU Int [Internet]. 2012 Dec [cited 2015 Nov 25];110(11 Pt C):E851-6. Available from: http://www.ncbi.nlm.nih.gov/pubmed/22958406

13. Lou P, Qin Y, Zhang P, Chen P, Zhang L, Chang G, et al. Association of sleep quality and quality of life in type 2 diabetes mellitus: a cross-sectional study in China. Diabetes Res Clin Pract [Internet]. 2015 Jan [cited 2015 Nov 25];107(1):69–76. Available from: http://www.sciencedirect.com/science/article/pii/S0168822714004604

14. Mellor A, Waters F, Olaithe M, McGowan H, Bucks RS. Sleep and aging: examining the effect of psychological symptoms and risk of sleep-disordered breathing. Behav Sleep Med

For peer review only - http://bmjopen.bmj.com/site/about/guidelines.xhtml

[Internet]. Routledge; 2014 Jan 28 [cited 2015 Nov 25];12(3):222–34. Available from: http://www.tandfonline.com/doi/abs/10.1080/15402002.2013.801343#.VlVu9HYrKHs

15. Landry GJ, Best JR, Liu-Ambrose T. Measuring sleep quality in older adults: a comparison using subjective and objective methods. Front Aging Neurosci [Internet]. Frontiers; 2015 Sep 7 [cited 2015 Sep 7];7. Available from: http://journal.frontiersin.org/article/10.3389/fnagi.2015.00166/abstract

16. Spiegelhalder K, Regen W, Prem M, Baglioni C, Nissen C, Feige B, et al. Reduced anterior internal capsule white matter integrity in primary insomnia. Hum Brain Mapp [Internet]. 2014 Jul 13 [cited 2015 Aug 4];35(7):3431–8. Available from: http://doi.wiley.com/10.1002/hbm.22412

17. Spiegel K, Tasali E, Leproult R, Van Cauter E. Effects of poor and short sleep on glucose metabolism and obesity risk. Nat Rev Endocrinol [Internet]. Nature Publishing Group; 2009 May [cited 2015 Aug 4];5(5):253–61. Available from: http://dx.doi.org/10.1038/nrendo.2009.23

18. Nebes RD, Buysse DJ, Halligan EM, Houck PR, Monk TH. Self-reported sleep quality predicts poor cognitive performance in healthy older adults. J Gerontol B Psychol Sci Soc Sci [Internet]. 2009 Mar [cited 2014 Sep 8];64(2):180–7. Available from: http://www.pubmedcentral.nih.gov/articlerender.fcgi?artid=2655169&tool=pmcentrez&rendertype=abstract

19. Shafto MA, Tyler LK, Dixon M, Taylor JR, Rowe JB, Cusack R, et al. The Cambridge Centre for Ageing and Neuroscience (Cam-CAN) study protocol: a cross-sectional, lifespan, multidisciplinary examination of healthy cognitive ageing. Neuroimage [Internet]. 2014 Oct 14 [cited 2014 Nov 14];14(204):1–25. Available from: http://www.sciencedirect.com/science/article/pii/S1053811915008150

20. Taylor JR, Williams N, Cusack R, Auer T, Shafto MA, Dixon M, et al. The Cambridge Centre for Ageing and Neuroscience (Cam-CAN) data repository: Structural and functional MRI, MEG, and cognitive data from a cross-sectional adult lifespan sample. Neuroimage [Internet]. 2015 Sep 12 [cited 2015 Sep 21]; Available from: http://www.sciencedirect.com/science/article/pii/S1053811915008150

21. Folstein MF, Folstein SE, McHugh PR. “Mini-mental state” a practical method for grading the cognitive state of patients for the clinician. J Psychiatr Res. 1975;12:189–98.

22. Regestein QR, Friebely J, Shifren JL, Scharf MB, Wiita B, Carver J, et al. Self-reported sleep in postmenopausal women. Menopause [Internet]. 2004 [cited 2015 Feb 17];11(2):198–207. Available from: http://journals.lww.com/menopausejournal/Abstract/2004/11020/Self_reported_sleep_in_postmenopausal_women.12.aspx

23. Curcio G, Ferrara M, De Gennaro L. Sleep loss, learning capacity and academic performance. Sleep Med Rev [Internet]. 2006 Oct [cited 2015 Sep 15];10(5):323–37. Available from: http://www.sciencedirect.com/science/article/pii/S1087079205001231

24. Scullin MK, Bliwise DL. Sleep, Cognition, and Normal Aging: Integrating a Half Century of Multidisciplinary Research. Perspect Psychol Sci [Internet]. 2015 Jan 14 [cited 2015 Jan 15];10(1):97–137. Available from: http://pps.sagepub.com/content/10/1/97.abstract

25. Altena E, Vrenken H, Van der Werf YD, Heuvel OA van den H, Someren EJW van, van den Heuvel OA, et al. Reduced Orbitofrontal and Parietal Gray Matter in Chronic Insomnia: A Voxel-Based Morphometric Study [Internet]. Vol. 67, BIOL PSYCHIATRY. 2010 [cited 2014 Jul 3]. p. 182–185. Available from:

For peer review only - http://bmjopen.bmj.com/site/about/guidelines.xhtml

http://www.sciencedirect.com/science/article/pii/S0006322309009548

26. Kamba M, Inoue Y, Higami S, Suto Y, Ogawa T, Chen W. Cerebral metabolic impairment in patients with obstructive sleep apnoea: an independent association of obstructive sleep apnoea with white matter change. J Neurol Neurosurg Psychiatry [Internet]. 2001 Sep;71(3):334–9. Available from: http://www.pubmedcentral.nih.gov/articlerender.fcgi?artid=1737534&tool=pmcentrez&rendertype=abstract

27. Harbison J, Gibson GJ, Birchall D, Zammit-Maempel I, Ford GA. White matter disease and sleep-disordered breathing after acute stroke. Neurology [Internet]. 2003 Oct 13 [cited 2016 Jan 7];61(7):959–63. Available from: http://www.neurology.org/content/61/7/959.short

28. Macey PM, Kumar R, Woo M a, Valladares EM, Yan-Go FL, Harper RM. Brain structural changes in obstructive sleep apnea. Sleep [Internet]. 2008 Jul;31(7):967–77. Available from: http://www.ncbi.nlm.nih.gov/pubmed/21300501

29. Ramos AR, Dong C, Rundek T, Elkind MS V, Boden-Albala B, Sacco RL, et al. Sleep duration is associated with white matter hyperintensity volume in older adults: the Northern Manhattan Study. J Sleep Res [Internet]. 2014 Jul 7 [cited 2014 Sep 8];i. Available from: http://www.ncbi.nlm.nih.gov/pubmed/25040435

30. Unger MM, Belke M, Menzler K, Heverhagen JT, Keil B, Stiasny-Kolster K, et al. Diffusion tensor imaging in idiopathic REM sleep behavior disorder reveals microstructural changes in the brainstem, substantia nigra, olfactory region, and other brain regions. Sleep [Internet]. American Academy of Sleep Medicine; 2010 Jun 1 [cited 2015 Dec 28];33(6):767–73. Available from: /pmc/articles/PMC2881532/?report=abstract

31. Macey PM, Henderson LA, Macey KE, Alger JR, Frysinger RC, Woo MA, et al. Brain morphology associated with obstructive sleep apnea. Am J Respir Crit Care Med [Internet]. American Thoracic Society; 2002 Nov 15 [cited 2014 Nov 16];166(10):1382–7. Available from: http://www.atsjournals.org/doi/abs/10.1164/rccm.200201-050OC#.VGkRkfmsXIk

32. Debette S, Markus HS. The clinical importance of white matter hyperintensities on brain magnetic resonance imaging: systematic review and meta-analysis. BMJ [Internet]. 2010 Jul 26 [cited 2016 Jan 12];341(jul26 1):c3666–c3666. Available from: http://www.bmj.com/content/341/bmj.c3666

33. Mädler B, Drabycz SA, Kolind SH, Whittall KP, MacKay AL. Is diffusion anisotropy an accurate monitor of myelination? Correlation of multicomponent T2 relaxation and diffusion tensor anisotropy in human brain. Magn Reson Imaging [Internet]. 2008 Sep [cited 2016 Jan 6];26(7):874–88. Available from: http://www.ncbi.nlm.nih.gov/pubmed/18524521

34. Maillard P, Fletcher E, Harvey D, Carmichael O, Reed B, Mungas D, et al. White matter hyperintensity penumbra. Stroke [Internet]. 2011 Jul 1 [cited 2016 Jan 6];42(7):1917–22. Available from: http://stroke.ahajournals.org/content/42/7/1917.short

35. Kievit RA, Davis SW, Griffiths J, Correia MM, Cam-CAN, Henson RN. A watershed model of individual differences in fluid intelligence. Neuropsychologia. 2016;91:186–98.

36. Grandner MA, Jackson NJ, Izci-Balserak B, Gallagher RA, Murray-Bachmann R, Williams NJ, et al. Social and Behavioral Determinants of Perceived Insufficient Sleep. Front Neurol [Internet]. Frontiers; 2015 Jan 5 [cited 2015 Aug 3];6:112. Available from: http://journal.frontiersin.org/article/10.3389/fneur.2015.00112/abstract

37. Leng Y, Wainwright NWJ, Cappuccio FP, Surtees PG, Hayat S, Luben R, et al. Daytime napping and the risk of all-cause and cause-specific mortality: a 13-year follow-up of a British

For peer review only

For peer review only - http://bmjopen.bmj.com/site/about/guidelines.xhtml

population. Am J Epidemiol [Internet]. 2014 May 1 [cited 2014 Aug 27];179(9):1115–24. Available from: http://www.pubmedcentral.nih.gov/articlerender.fcgi?artid=3992821&tool=pmcentrez&rendertype=abstract

38. Leng Y, Cappuccio FP, Wainwright NWJ, Surtees PG, Luben R, Brayne C, et al. Sleep duration and risk of fatal and nonfatal stroke: a prospective study and meta-analysis. Neurology [Internet]. 2015 Mar 17 [cited 2016 Jan 28];84(11):1072–9. Available from: http://www.neurology.org/content/early/2015/02/25/WNL.0000000000001371.abstract

39. Hoevenaar-Blom MP, Spijkerman AMW, Kromhout D, van den Berg JF, Verschuren WMM. Sleep duration and sleep quality in relation to 12-year cardiovascular disease incidence: the MORGEN study. Sleep [Internet]. 2011 Nov [cited 2016 Jan 6];34(11):1487–92. Available from: http://www.pubmedcentral.nih.gov/articlerender.fcgi?artid=3198203&tool=pmcentrez&rendertype=abstract

40. Strine TW, Chapman DP. Associations of frequent sleep insufficiency with health-related quality of life and health behaviors. Sleep Med [Internet]. 2005 Jan [cited 2015 Oct 19];6(1):23–7. Available from: http://www.sciencedirect.com/science/article/pii/S1389945704001078

41. Taheri S, Lin L, Austin D, Young T, Mignot E. Short sleep duration is associated with reduced leptin, elevated ghrelin, and increased body mass index. PLoS Med [Internet]. Public Library of Science; 2004 Dec 7 [cited 2015 Nov 1];1(3):e62. Available from: http://journals.plos.org/plosmedicine/article?id=10.1371/journal.pmed.0010062

42. Roberts RE, Shema SJ, Kaplan GA, Strawbridge WJ. Sleep Complaints and Depression in an Aging Cohort: A Prospective Perspective. Am J Psychiatry [Internet]. American Psychiatric Publishing; 2000 Jan 1 [cited 2015 Jun 16];157(1):81–8. Available from: http://ajp.psychiatryonline.org/doi/10.1176/ajp.157.1.81

43. Breslau N, Roth T, Rosenthal L, Andreski P. Sleep disturbance and psychiatric disorders: A longitudinal epidemiological study of young Adults. Biol Psychiatry [Internet]. 1996 Mar [cited 2015 Apr 8];39(6):411–8. Available from: http://www.sciencedirect.com/science/article/pii/0006322395001883

44. Kaneita Y, Ohida T, Uchiyama M, Takemura S, Kawahara K, Yokoyama E, et al. The Relationship Between Depression and Sleep Disturbances: A Japanese Nationwide General Population Survey. J Clin Psychiatry [Internet]. 2006 Feb [cited 2015 Jun 16];67(2):196–203. Available from: http://www.ncbi.nlm.nih.gov/pubmed/16566613

45. Fried EI, Nesse RM. Depression sum-scores don't add up: why analyzing specific depression symptoms is essential. BMC Med [Internet]. 2015 Apr 6 [cited 2015 Apr 9];13(1):72. Available from: http://www.biomedcentral.com/1741-7015/13/72

46. Novati A, Hulshof HJ, Koolhaas JM, Lucassen PJ, Meerlo P. Chronic sleep restriction causes a decrease in hippocampal volume in adolescent rats, which is not explained by changes in glucocorticoid levels or neurogenesis. Neuroscience [Internet]. 2011 Sep 8 [cited 2015 Jan 20];190:145–55. Available from: http://www.sciencedirect.com/science/article/pii/S0306452211007111

47. Ramsawh HJ, Stein MB, Belik S-L, Jacobi F, Sareen J. Relationship of anxiety disorders, sleep quality, and functional impairment in a community sample. J Psychiatr Res [Internet]. 2009 Jul [cited 2015 Dec 7];43(10):926–33. Available from: http://www.sciencedirect.com/science/article/pii/S0022395609000211

For peer review only - http://bmjopen.bmj.com/site/about/guidelines.xhtml

48. Zigmond AS, Snaith RP. The hospital anxiety and depression scale. Acta Psychiatr Scand [Internet]. 1983 Jun [cited 2014 Jul 11];67(6):361–70. Available from: http://www.ncbi.nlm.nih.gov/pubmed/6880820

49. Wechsler CJ. Wechsler Memory Scale. 3d UK. London: Harcourt; 1999.

50. Cattell RB. Abilities: their structure, growth, and action. Boston: Houghton-Mifflin; 1971.

51. Mioshi E, Dawson K, Mitchell J, Arnold R, Hodges JR. The Addenbrooke's Cognitive Examination Revised (ACE-R): a brief cognitive test battery for dementia screening. Int J Geriatr Psychiatry [Internet]. 2006 Nov [cited 2015 Sep 29];21(11):1078–85. Available from: http://www.ncbi.nlm.nih.gov/pubmed/16977673

52. Hua K, Zhang J, Wakana S, Jiang H, Li X, Reich DS, et al. Tract probability maps in stereotaxic spaces: analyses of white matter anatomy and tract-specific quantification. Neuroimage. 2008 Jan;39(1):336–47.

53. McGee DL, Liao Y, Cao G, Cooper RS. Self-reported Health Status and Mortality in a Multiethnic US Cohort. Am J Epidemiol [Internet]. 1999 Jan 1 [cited 2015 Nov 25];149(1):41–6. Available from: http://aje.oxfordjournals.org/content/149/1/41.short

54. Deurenberg P, Weststrate JA, Seidell JC. Body mass index as a measure of body fatness: age- and sex-specific prediction formulas. Br J Nutr [Internet]. Cambridge University Press; 2007 Mar 9 [cited 2015 Oct 7];65(2):105. Available from: http://journals.cambridge.org/abstract_S0007114591000193

55. Liang F, Paulo R, Molina G, Clyde MA, Berger JO. Mixtures of g Priors for Bayesian Variable Selection. J Am Stat Assoc [Internet]. Taylor & Francis; 2008 Mar 1 [cited 2015 Oct 7];103(481):410–23. Available from: http://amstat.tandfonline.com/doi/abs/10.1198/016214507000001337#.VhTxTPl3lpg

56. Rouder JN, Morey RD. Default Bayes Factors for Model Selection in Regression. Multivariate Behav Res [Internet]. Taylor & Francis Group; 2012 Nov 17 [cited 2015 Jun 16];47(6):877–903. Available from: http://www.tandfonline.com/doi/abs/10.1080/00273171.2012.734737

57. Wagenmakers E-J. A practical solution to the pervasive problems ofp values. Psychon Bull Rev [Internet]. 2007 Oct [cited 2015 Jun 16];14(5):779–804. Available from: http://www.springerlink.com/index/10.3758/BF03194105

58. Wei T, Liang X, He Y, Zang Y, Han Z, Caramazza A, et al. Predicting conceptual processing capacity from spontaneous neuronal activity of the left middle temporal gyrus. J Neurosci. 2012 Jan;32(2):481–9.

59. Wetzels R, Matzke D, Lee MD, Rouder JN, Iverson GJ, Wagenmakers E-J. Statistical Evidence in Experimental Psychology: An Empirical Comparison Using 855 t Tests. Perspect Psychol Sci [Internet]. 2011 May 18 [cited 2015 May 12];6(3):291–8. Available from: http://pps.sagepub.com/content/6/3/291.short

60. Gelman A, Hill J, Yajima M. Why We (Usually) Don't Have to Worry About Multiple Comparisons. J Res Educ Eff [Internet]. Taylor & Francis Group; 2012 Apr 3 [cited 2014 Jul 15];5(2):189–211. Available from: http://www.tandfonline.com/doi/abs/10.1080/19345747.2011.618213

61. Jeffreys H. Theory of Probability. Oxford: Oxford University Press; 1961.

62. Morey RD, Rouder JN. BayesFactor. CRAN; 2015.

63. Team. R: a language and environment for statistical computing. Vienna; 2013.

For peer review only - http://bmjopen.bmj.com/site/about/guidelines.xhtml

64. Linzer DA, Lewis JB. poLCA: An R Package for Polytomous Variable Latent Class Analysis [Internet]. Journal of Statistical Software. 2011 [cited 2014 Sep 8]. p. 42: 10. Available from: http://www.jstatsoft.org/v42/i10/paper

65. Schwarz G. Estimating the Dimension of a Model. Ann Stat [Internet]. Institute of Mathematical Statistics; 1978 Mar 1 [cited 2015 Jun 16];6(2):461–4. Available from: http://projecteuclid.org/euclid.aos/1176344136

66. Dayton CM, Macready GB. Concomitant-Variable Latent-Class Models. J Am Stat Assoc [Internet]. Taylor & Francis; 1988 Mar [cited 2014 Sep 8];83(401):173–8. Available from: http://www.tandfonline.com/doi/abs/10.1080/01621459.1988.10478584

67. Linzer DA, Lewis J. poLCA : Polytomous Variable Latent Class Analysis Version 1 . 4. J Stat Softw. 2011;42(10):1–29.

68. Wickham H. ggplot2: Elegant Graphics for Data Analysis [Internet]. Springer Science & Business Media; 2009 [cited 2015 Aug 4]. 221 p. Available from: https://books.google.com/books?hl=en&lr=&id=bes-AAAAQBAJ&pgis=1

69. Miller E. Verbal fluency as a function of a measure of verbal intelligence and in relation to different types of cerebral pathology. Br J Clin Psychol [Internet]. 1984 Feb 12 [cited 2016 Jan 7];23(1):53–7. Available from: http://doi.wiley.com/10.1111/j.2044-8260.1984.tb00626.x

70. Troyer AK, Moscovitch M, Winocur G. Clustering and switching as two components of verbal fluency: Evidence from younger and older healthy adults. Neuropsychology [Internet]. 1997 [cited 2016 Jan 7];11(1). Available from: http://psycnet.apa.orgjournals/neu/11/1/138

71. Kievit RA, Davis SW, Griffiths JD, Correia MM, Henson RNA. A watershed model of individual differences in fluid intelligence. bioRxiv [Internet]. Cold Spring Harbor Labs Journals; 2016 Feb 26 [cited 2016 Mar 29];41368. Available from: http://www.biorxiv.org/content/early/2016/02/26/041368.abstract

72. Briones B, Adams N, Strauss M, Rosenberg C, Whalen C, Carskadon M, et al. Relationship between sleepiness and general health status. Sleep [Internet]. 1996 Sep [cited 2015 Aug 4];19(7):583–8. Available from: http://www.ncbi.nlm.nih.gov/pubmed/8899938

73. Cizza G, Skarulis M, Mignot E. A link between short sleep and obesity : Building the evidence for causation. Sleep [Internet]. American Academy of Sleep Medicine; 2005 [cited 2016 Jan 12];28(10):1217–20. Available from: http://cat.inist.fr/?aModele=afficheN&cpsidt=17179376

74. Gangwisch JE, Malaspina D, Boden-Albala B, Heymsfield SB. Inadequate sleep as a risk factor for obesity: analyses of the NHANES I. Sleep [Internet]. 2005 Oct [cited 2015 Sep 3];28(10):1289–96. Available from: http://europepmc.org/abstract/med/16295214

75. Lustberg L, Reynolds CF. Depression and insomnia: questions of cause and effect. Sleep Med Rev [Internet]. 2000 Jun [cited 2015 Dec 28];4(3):253–62. Available from: http://www.sciencedirect.com/science/article/pii/S1087079299900758

76. Sbarra DA, Allen JJB. Decomposing depression: On the prospective and reciprocal dynamics of mood and sleep disturbances.

77. Lim ASP, Ellison BA, Wang JL, Yu L, Schneider JA, Buchman AS, et al. Sleep is related to neuron numbers in the ventrolateral preoptic/intermediate nucleus in older adults with and without Alzheimer's disease. Brain [Internet]. 2014 Oct 20 [cited 2015 Dec 16];137(Pt 10):2847–61. Available from: http://brain.oxfordjournals.org/content/early/2014/08/11/brain.awu222

78. Schaie KW. The course of adult intellectual development.

79. Raz N, Lindenberger U. Only time will tell: Cross-sectional studies offer no solution to the

For peer review only - http://bmjopen.bmj.com/site/about/guidelines.xhtml

age–brain–cognition triangle: Comment on Salthouse (2011).

80. Wiberg M, Sundstrom A. A Comparison of Two Approaches to Correction of Restriction of Range in Correlation Analysis. Pract Assessment, Res Eval [Internet]. Dr. Lawrence M. Rudner. e-mail: editor@pareonline.net; Web site: http://pareonline.net; 2009 Feb 28 [cited 2016 Feb 19];14(5). Available from: http://eric.ed.gov/?id=EJ933658

For peer review only

For peer review only - http://bmjopen.bmj.com/site/about/guidelines.xhtml

Legends

Figure 1. Descriptive interpretation of Bayes Factors

Figure 2. Latent Class Analysis. Panel A shows the sleep quality profiles for each of the four classes. Panel B shows the conditional probability of belonging to each class across the lifespan.

Figure 3. Simple regressions between sleep components and Cognitive Health.
The strength of the effect is colour-coded by Bayes Factor, and the effect size is shown as r-squared (as a percentage out of 100). Sample varies across components and measures due to varying missingness. Cattell and Reaction Time were measured only in the imaging cohort: mean N = 648, N=11.11. Sample sizes for 5 other domains are similar: mean N= 2300.25, SD= 65.57)

Figure 4. Multiple regressions between sleep components and Neural Health. Each cell represents the relationship between a sleep component and the mean neural health in a given tract as index by Fractional Anisotropy. Numbers represent R-squared, the sample size is show in the last column. Strong associations are observed between measures of Sleep Efficiency and multiple tracts, along with sporadic associations between other components and tracts. White matter tracts abbreviations: Uncinate fasciculus (UNC), superior longitudinal fasciculus (SLF), inferior longitudinal fasciculus (ILF), inferior Fronto-occipital fasciculus (IFOF), forceps minor (FMin), forceps major (FMaj), cerebrospinal tract (CST), the ventral cingulate gyrus (CINGHipp), the dorsal cingulate gyrus (CING), and the anterior thalamic radiations (ATR). N varies slightly across components due to varying missingness (N mean = 631.325, SD = 10.32).

Figure 5 Physical health and sleep quality. Numbers represent Rsquared, the sample size is show in the last column. Strong associations between general indices of health and sleep quality are found, and several more modest relationships with BMI and sleep quality. Self-reported health (12 month and General) were measured in the full cohort (Mean = 2315.37, SD=66.29), the other indicators were measured in the imaging cohort only (Mean = 569.87, SD= 11.16).

Figure 6. Interaction between sleep quality and anxiety. (N=724,age 18.48 to 46.2) compared to the oldest third of participants (N=725, age 71.79 to 98.88).

For peer review only - http://bmjopen.bmj.com/site/about/guidelines.xhtml

Bayes Factor BF10	Log BF10	Tileplot colour	Description (Jeffreys, 1961)
>100	>4.6		Extreme evidence for H1
30 – 100	3.4 – 4-6		Very strong evidence for H1
10 – 30	2.3 – 3.4		Strong evidence for H1
3 – 10	1.098 – 2.3		Substantial evidence for H1
1 – 3	1 – 1.098		Anecdotal evidence for H1
1	0		No evidence either way
1/3 – 1	-1.098 – -1		Anecdotal evidence for H0
1/3 - 1/10	-2.3 – -1.098		Substantial evidence for H0
1/10 - 1/30	-3.4 – -2.3		Strong evidence for H0
1/30 - 1/100	-4.6 – - 3.4		Very strong evidence for H0
<1/100	< -4.6		Extreme evidence for H0

Descriptive interpretation of Bayes Factors
insert Figure 1 here
338x190mm (96 x 96 DPI)

review only

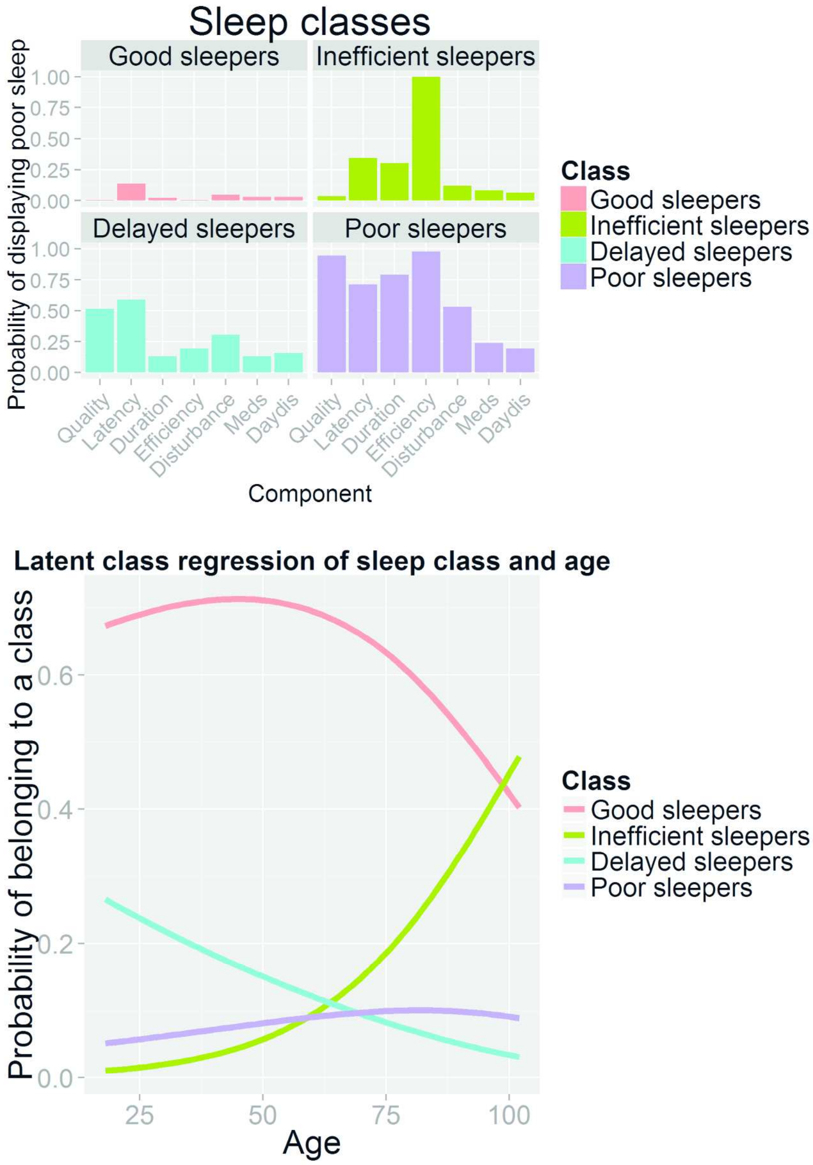

Latent Class Analysis. Panel A shows the sleep quality profiles for each of the four classes. Panel B shows the conditional probability of belonging to each class across the lifespan.
insert Figure 2 here
214x282mm (300 x 300 DPI)

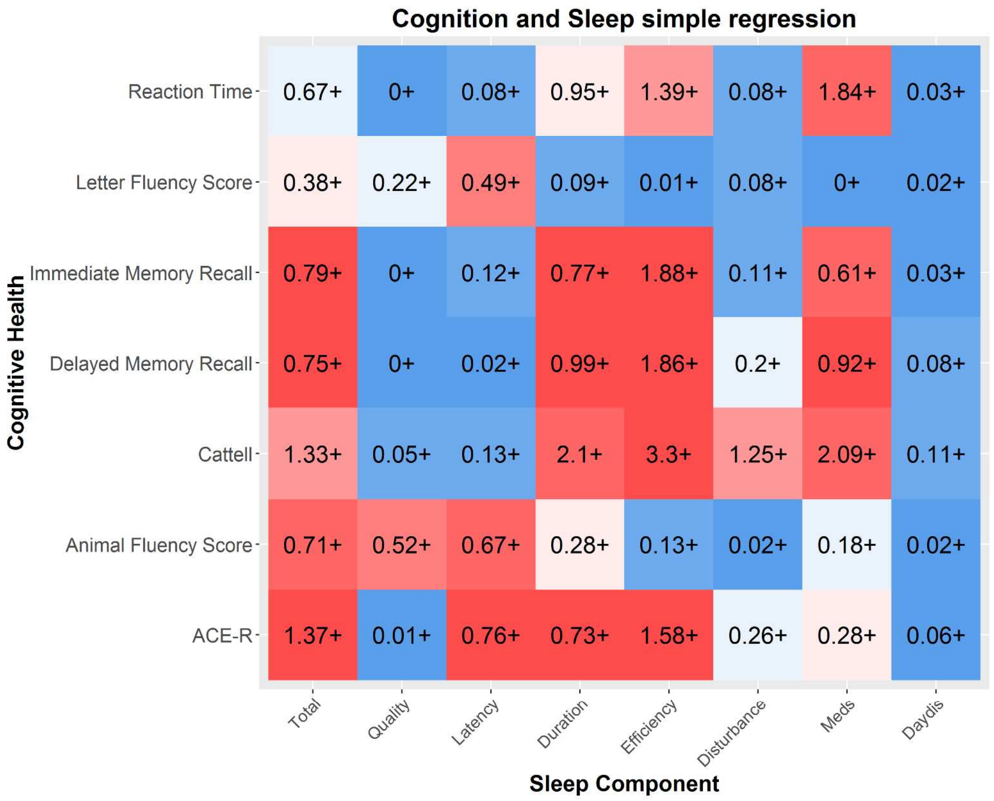

Simple regressions between sleep components and Cognitive Health.
The strength of the effect is colour-coded by Bayes Factor, and the effect size is shown as r-squared (as a percentage out of 100). Sample varies across components and measures due to varying missingness. Cattell and Reaction Time were measured only in the imaging cohort: mean N = 648, N=11.11. Sample sizes for 5 other domains are similar: mean N= 2300.25, SD= 65.57)

insert Figure 3 here
254x203mm (300 x 300 DPI)

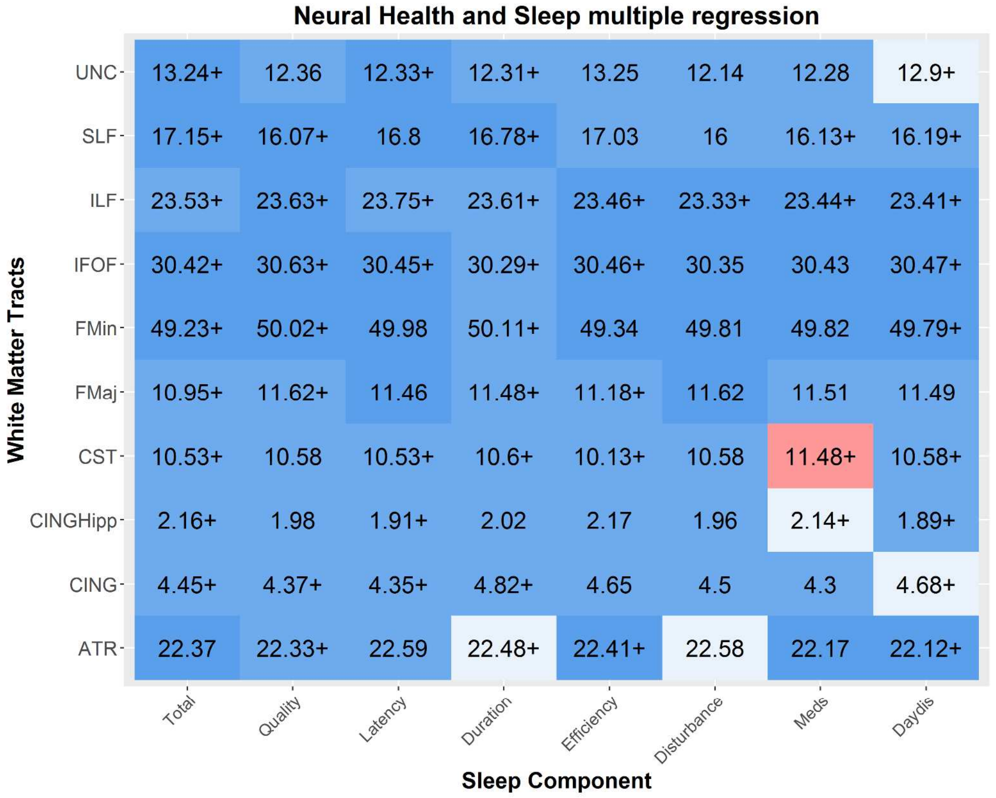

Multiple regressions between sleep components and Neural Health. Each cell represents the relationship between a sleep component and the mean neural health in a given tract as index by Fractional Anisotropy. Numbers represent R-squared, the sample size is show in the last column. Strong associations are observed between measures of Sleep Efficiency and multiple tracts, along with sporadic associations between other components and tracts. White matter tracts abbreviations: Uncinate fasciculus (UNC), superior longitudinal fasciculus (SLF), inferior longitudinal fasciculus (ILF), inferior Fronto-occipital fasciculus (IFOF), forceps minor (FMin), forceps major (FMaj), cerebrospinal tract (CST), the ventral cingulate gyrus (CINGHipp), the dorsal cingulate gyrus (CING), and the anterior thalamic radiations (ATR). N varies slightly across components due to varying missingness (N mean = 631.325, SD = 10.32).
insert Figure 4 here
254x203mm (300 x 300 DPI)

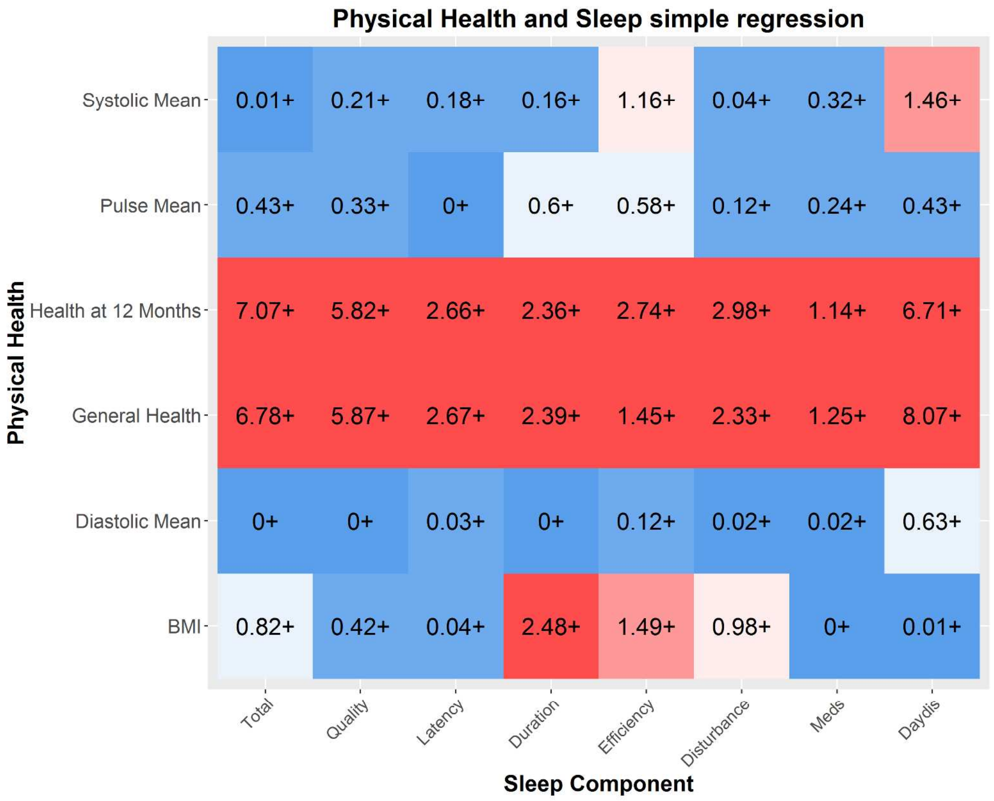

Physical health and sleep quality. Numbers represent Rsquared, the sample size is show in the last column. Strong associations between general indices of health and sleep quality are found, and several more modest relationships with BMI and sleep quality. Self-reported health (12 month and General) were measured in the full cohort (Mean = 2315.37, SD=66.29), the other indicators were measured in the imaging cohort only (Mean = 569.87, SD= 11.16).
insert Figure 5
254x203mm (300 x 300 DPI)

only

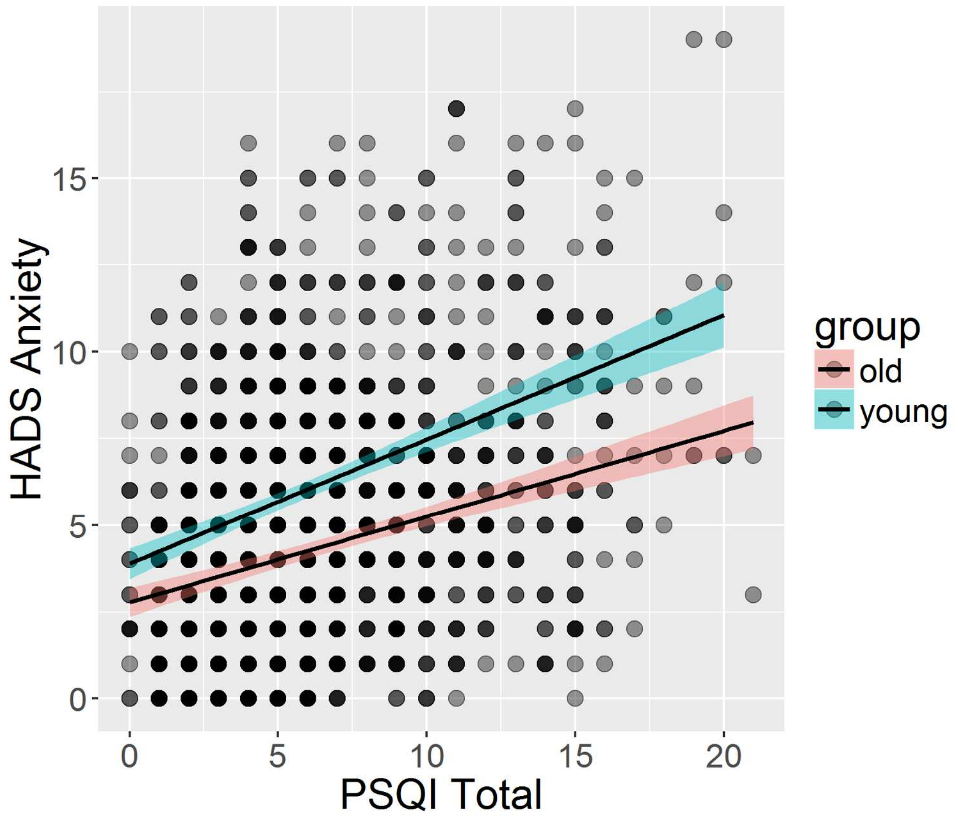

Interaction between sleep quality and anxiety. (N=724,age 18.48 to 46.2) compared to the oldest third of participants (N=725, age 71.79 to
insert Figure 6
152x127mm (300 x 300 DPI)

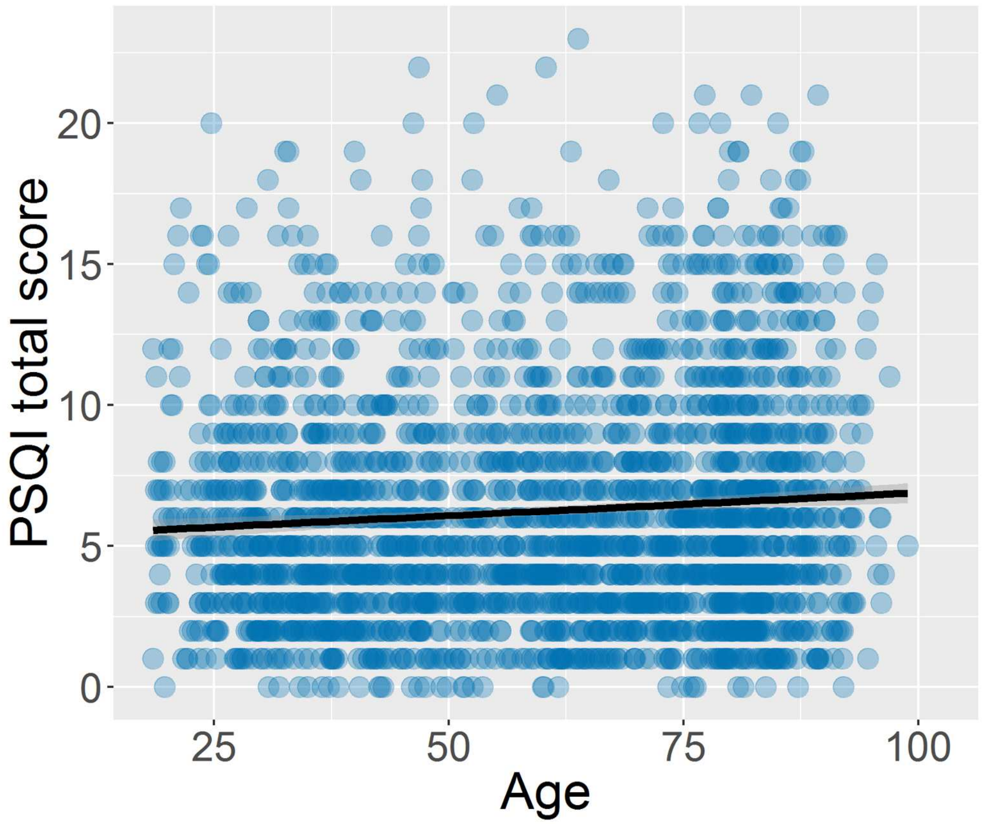

152x127mm (300 x 300 DPI)

For peer review only

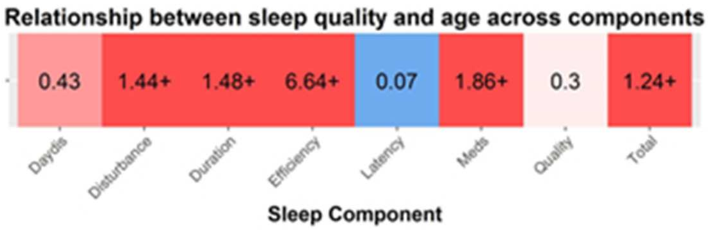

96x32mm (96 x 96 DPI)

For peer review only

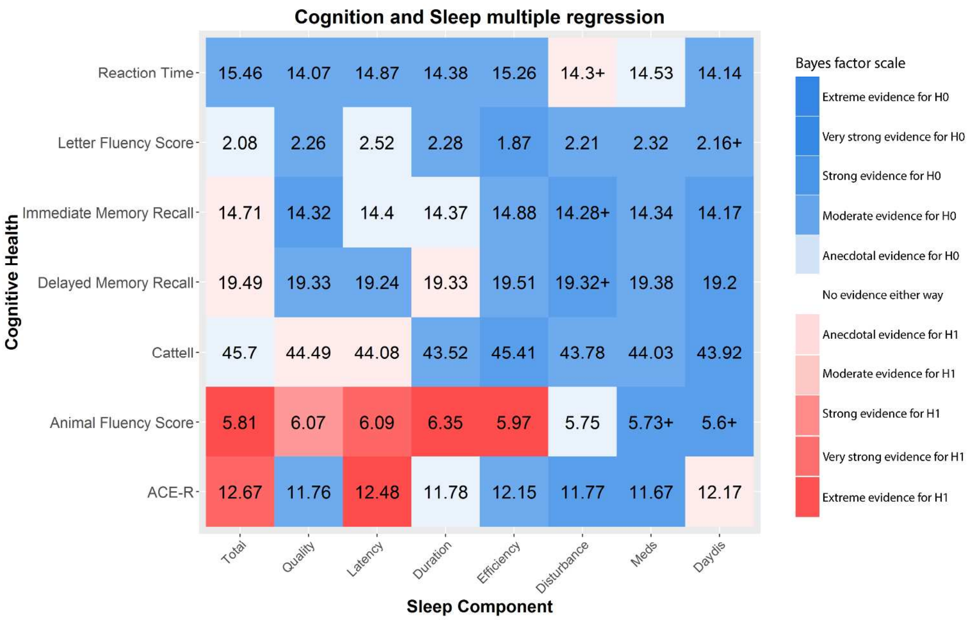

329x214mm (300 x 300 DPI)

For peer review only

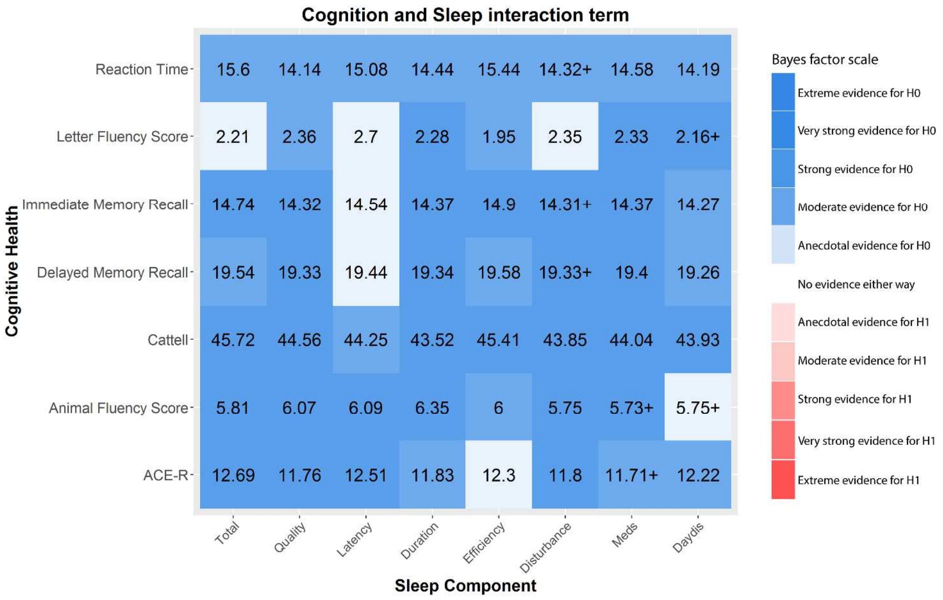

340x213mm (300 x 300 DPI)

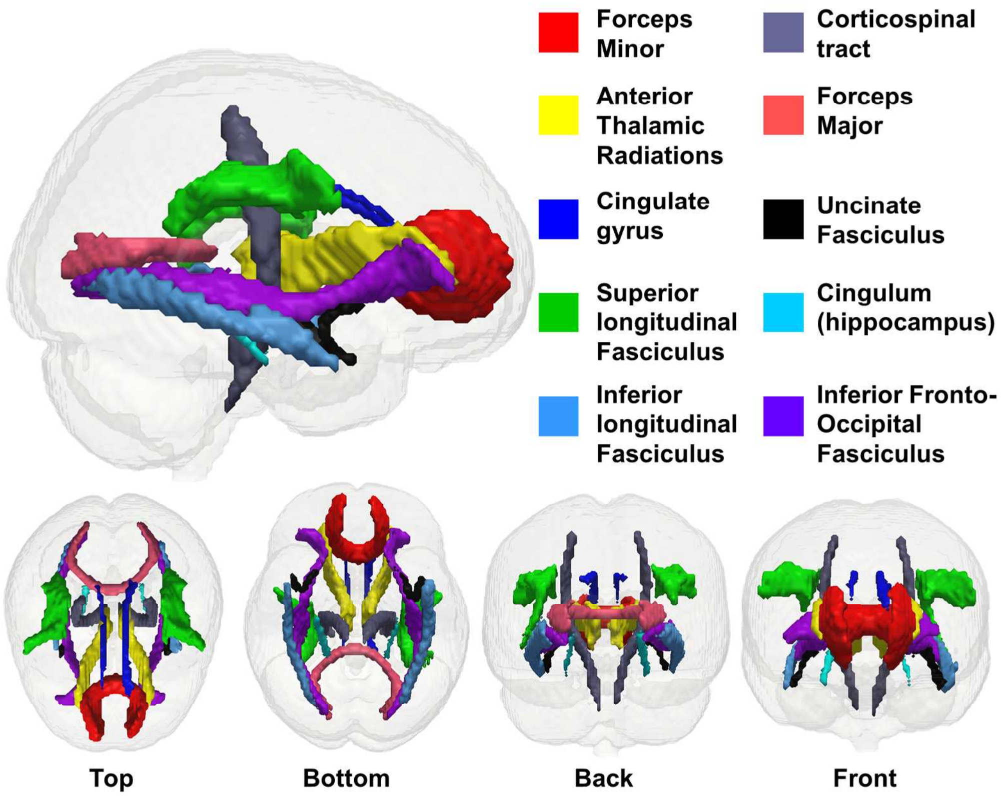

500x400mm (96 x 96 DPI)

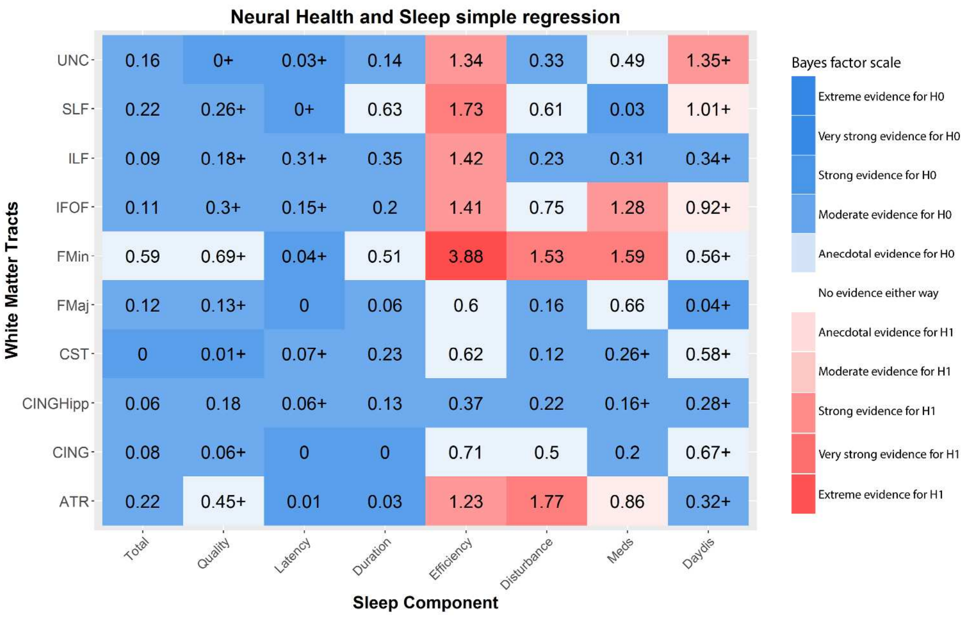

331x213mm (300 x 300 DPI)

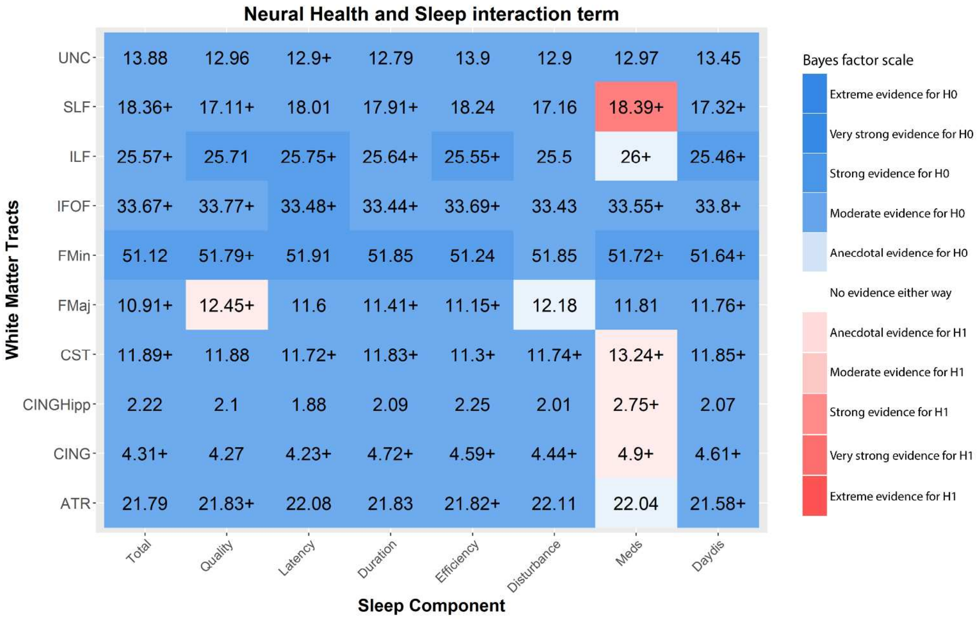

327x215mm (300 x 300 DPI)

view only

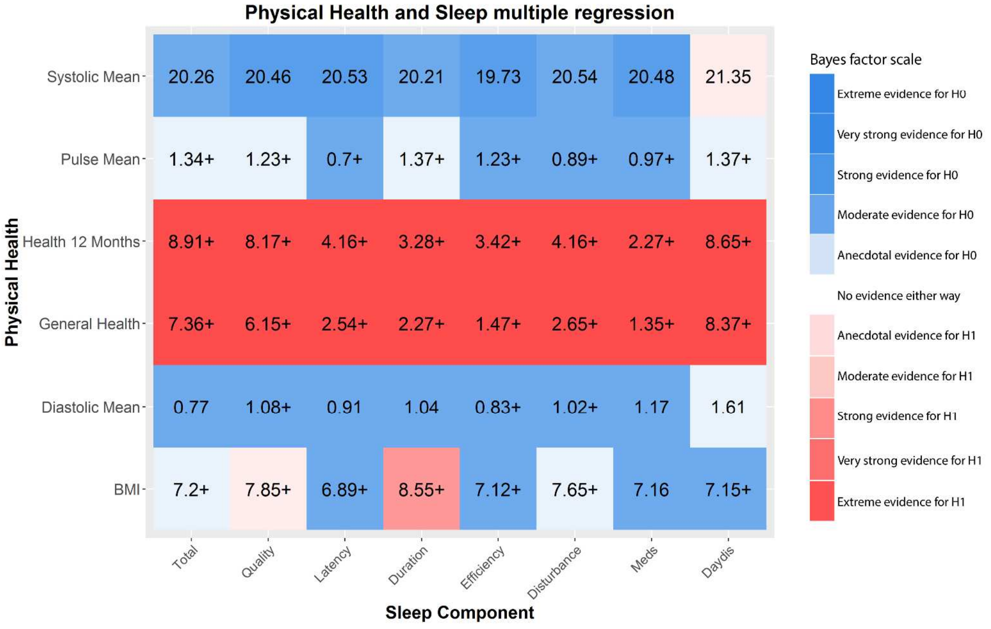

324x208mm (300 x 300 DPI)

For peer review only

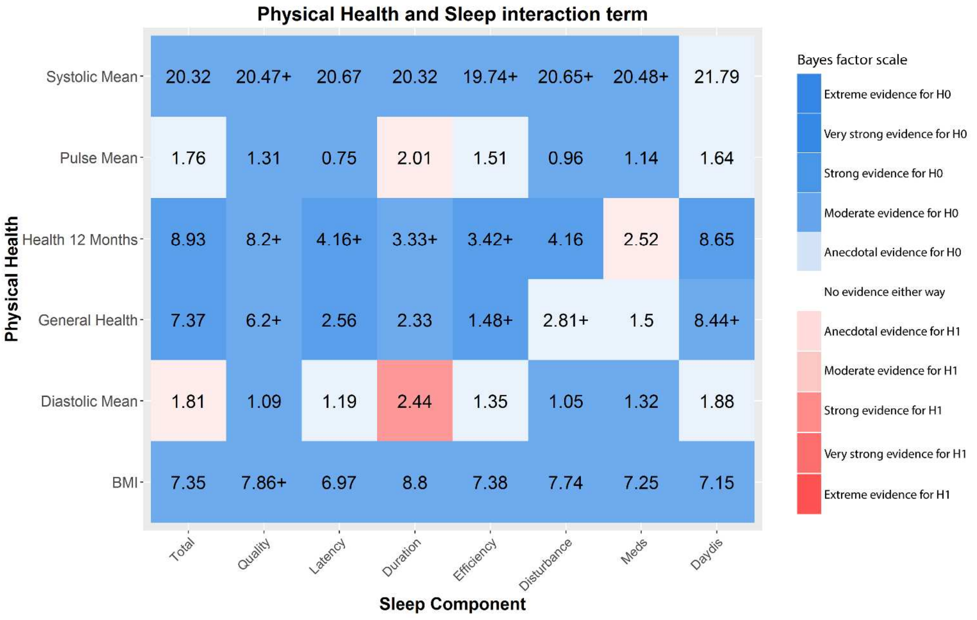

329x214mm (300 x 300 DPI)

view only

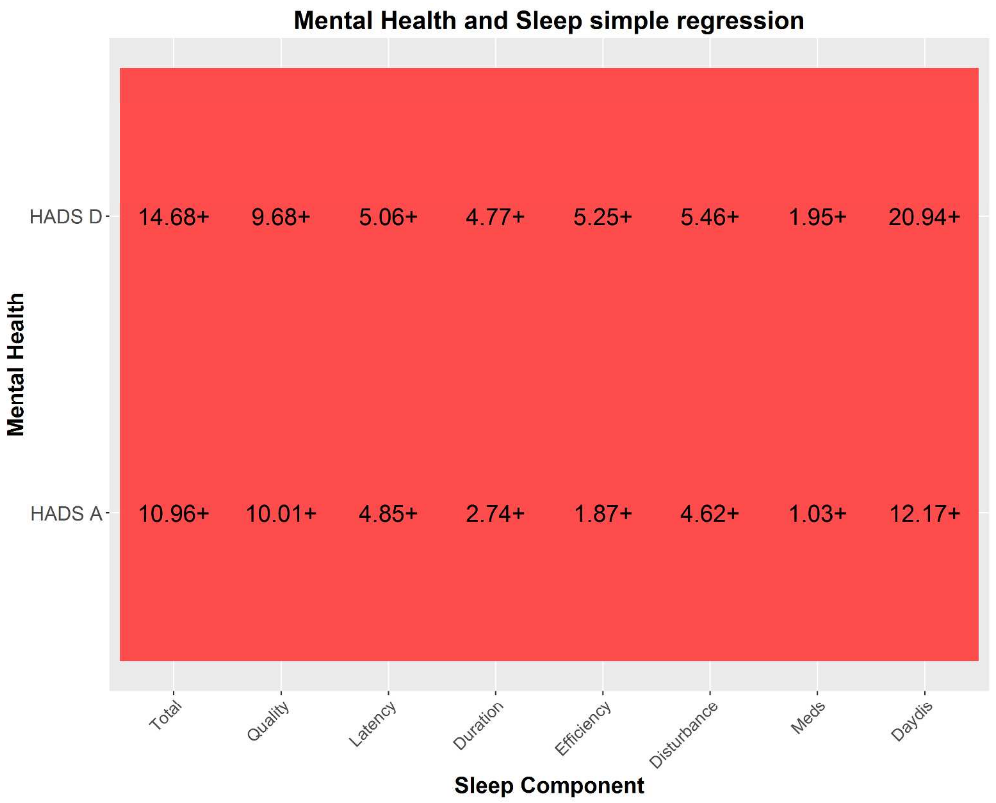

254x203mm (300 x 300 DPI)

STROBE 2007 (v4) Statement—Checklist of items that should be included in reports of *cohort studies*

Section/Topic	Item #	Recommendation	Reported on page #
**Title and abstract**	1	(*a*) Indicate the study's design with a commonly used term in the title or the abstract	1
		(*b*) Provide in the abstract an informative and balanced summary of what was done and what was found	2
**Introduction**			
Background/rationale	2	Explain the scientific background and rationale for the investigation being reported	4
Objectives	3	State specific objectives, including any prespecified hypotheses	5 (lines 87-91)
**Methods**			
Study design	4	Present key elements of study design early in the paper	5
Setting	5	Describe the setting, locations, and relevant dates, including periods of recruitment, exposure, follow-up, and data collection	5 (project protocol)
Participants	6	(*a*) Give the eligibility criteria, and the sources and methods of selection of participants. Describe methods of follow-up	5 (project protocol)
		(*b*) For matched studies, give matching criteria and number of exposed and unexposed	NA
Variables	7	Clearly define all outcomes, exposures, predictors, potential confounders, and effect modifiers. Give diagnostic criteria, if applicable	5-9
Data sources/ measurement	8*	For each variable of interest, give sources of data and details of methods of assessment (measurement). Describe comparability of assessment methods if there is more than one group	6-11
Bias	9	Describe any efforts to address potential sources of bias	N/A
Study size	10	Explain how the study size was arrived at	(project protocol)
Quantitative variables	11	Explain how quantitative variables were handled in the analyses. If applicable, describe which groupings were chosen and why	10
Statistical methods	12	(*a*) Describe all statistical methods, including those used to control for confounding	10-13
		(*b*) Describe any methods used to examine subgroups and interactions	10
		(*c*) Explain how missing data were addressed	11
		(*d*) If applicable, explain how loss to follow-up was addressed	N/A
		(*e*) Describe any sensitivity analyses	N/A

For peer review only

**Results**			
Participants	13*	(a) Report numbers of individuals at each stage of study—eg numbers potentially eligible, examined for eligibility, confirmed eligible, included in the study, completing follow-up, and analysed	14-22
		(b) Give reasons for non-participation at each stage	(Project Proposal)
		(c) Consider use of a flow diagram	NA
Descriptive data	14*	(a) Give characteristics of study participants (eg demographic, clinical, social) and information on exposures and potential confounders	(Project Protocol)
		(b) Indicate number of participants with missing data for each variable of interest	
		(c) Summarise follow-up time (eg, average and total amount)	(Project Protocol)
Outcome data	15*	Report numbers of outcome events or summary measures over time	NA
Main results	16	(*a*) Give unadjusted estimates and, if applicable, confounder-adjusted estimates and their precision (eg, 95% confidence interval). Make clear which confounders were adjusted for and why they were included	14-22
		(*b*) Report category boundaries when continuous variables were categorized	NA
		(*c*) If relevant, consider translating estimates of relative risk into absolute risk for a meaningful time period	NA
Other analyses	17	Report other analyses done—eg analyses of subgroups and interactions, and sensitivity analyses	14-22
**Discussion**			
Key results	18	Summarise key results with reference to study objectives	22-26
**Limitations**			
Interpretation	20	Give a cautious overall interpretation of results considering objectives, limitations, multiplicity of analyses, results from similar studies, and other relevant evidence	25-26
Generalisability	21	Discuss the generalisability (external validity) of the study results	25-26
**Other information**			
Funding	22	Give the source of funding and the role of the funders for the present study and, if applicable, for the original study on which the present article is based	3

*Give information separately for cases and controls in case-control studies and, if applicable, for exposed and unexposed groups in cohort and cross-sectional studies.

Note: An Explanation and Elaboration article discusses each checklist item and gives methodological background and published examples of transparent reporting. The STROBE checklist is best used in conjunction with this article (freely available on the Web sites of PLoS Medicine at http://www.plosmedicine.org/, Annals of Internal Medicine at http://www.annals.org/, and Epidemiology at http://www.epidem.com/). Information on the STROBE Initiative is available at www.strobe-statement.org.

For peer review only

BMJ Open

How are age-related differences in sleep quality associated with health outcomes? An epidemiological investigation in a UK cohort of 2406 adults

Journal:	*BMJ Open*
Manuscript ID	bmjopen-2016-014920.R1
Article Type:	Research
Date Submitted by the Author:	15-Feb-2017
Complete List of Authors:	Gadie, Andrew; MRC Cognition and Brain Sciences Unit<br>Shafto, Meredith; University of Cambridge, Center for Speech, Language and the Brain<br>Leng, Yue; University of Cambridge; University of California San Francisco, School of Medicine<br>Cam-CAN, _; University of Cambridge, Center for Sleep, language and the brain<br>Kievit, Rogier; MRC CBSU
**Primary Subject Heading**:	Epidemiology
Secondary Subject Heading:	Neurology, Mental health, Public health, Geriatric medicine
Keywords:	Ageing, SLEEP MEDICINE, cognition, MENTAL HEALTH, Neurobiology < BASIC SCIENCES

SCHOLARONE™ Manuscripts

For peer review only

How are age-related difference in sleep quality associated with health outcomes? An epidemiological investigation in a UK cohort of 2406 adults

Andrew Gadie[1]

Meredith Shafto[2]

Yue Leng[3]

Cam-CAN[4]

Rogier A. Kievit[1]*

*Corresponding author: rogier.kievit@mrc-cbu.cam.ac.uk
1 MRC Cognition and Brain Sciences Unit, 15 Chaucer Rd, Cambridge, CB2 7EF, United Kingdom
2 Department of Psychology, University of Cambridge, Downing Street, Cambridge, CB2 3EB, United Kingdom
3 University of California, San Francisco
4 Cambridge Centre for Ageing and Neuroscience (Cam-CAN), University of Cambridge and MRC Cognition and Brain Sciences Unit, Cambridge, UK, www.cam-can.com

For peer review only

Abstract

Objectives To examine age related differences in self-reported sleep quality and their associations with health outcomes across four domains: Physical Health, Cognitive Health, Mental Health and Neural Health.

Setting Cam-CAN is a cohort study in East Anglia/England, which collected self-reported health and lifestyle questions as well as a range of objective measures from healthy adults.

Participants 2406 healthy adults (age 18-98) answered questions about their sleep quality (Pittsburgh Sleep Quality Index) and measures of Physical, Cognitive, Mental, and Neural Health. A subset of 641 individuals provided measures of brain structure.

Main outcome measures Pittsburgh Sleep Quality Index scores (PSQI) of sleep, and scores across tests within the four domains of health. Latent Class Analysis (LCA) is used to identify sleep types across the lifespan. Bayesian regressions quantify the presence, and absence, of relationships between sleep quality and health measures.

Results Better sleep is generally associated with better health outcomes, strongly so for mental health, moderately for cognitive and physical health, but not for sleep quality and neural health. Latent Class Analysis identified four sleep types: 'Good sleepers' (68.1%, most frequent in middle age), 'inefficient sleepers' (14.01%, most frequent in old age), 'Delayed sleepers' (9.28%, most frequent in young adults) and 'poor sleepers' (8.5%, most frequent in old age). There is little evidence for interactions between sleep quality and age on health outcomes. Finally, we observe u-shaped associations between sleep duration and mental health (depression and anxiety) as well as self-reported general health, such that both short and long sleep were associated with poorer outcomes.

Conclusions Lifespan changes in sleep quality are multifaceted and not captured well by summary measures, but instead as partially independent symptoms that vary in prevalence across the lifespan. Better self-reported sleep is associated with better health outcomes, and the strength

For peer review only

of these associations differs across health domains. Notably, we do observed associations between self-reported sleep quality and white matter.

Funding Biotechnology and Biological Sciences Research Council (grant number BB/H008217/1). RAK is supported by the Wellcome Trust (grant number 107392/Z/15/Z and the UK Medical Research Council (MC-A060-5PR61).

Keywords

Ageing, sleep quality, healthy ageing, cognition, mental health, cognition, white matter, physical health

Strengths and limitations of this study

- Broad phenotypic assessment of healthy ageing across multiple health domains
- Advanced analytic techniques (i.e. Latent Class Analysis regression) allows new insights
- A uniquely large neuroimaging sample combined with Bayesian inference allows for quantification of evidence for the null hypothesis
- Subjective sleep measures may have drawbacks in older samples
- Cross-sectional data precludes modelling of within subject changes

For peer review only

For peer review only - http://bmjopen.bmj.com/site/about/guidelines.xhtml

BACKGROUND

Sleep is a fundamental human behaviour, with humans spending almost a third of their lives asleep. Regular and sufficient sleep has been shown to benefit human physiology through a number of different routes, ranging from consolidation of memories (1) to removal of free radicals (2) and neurotoxic waste (3). Sleep patterns are known to change across the lifespan in various ways. including decreases in quantity and quality of sleep (4), with up to 50% of older adults report difficulties initiating and/or maintaining sleep (5). A meta-analysis of over 65 studies reflecting 3577 subjects across the lifespan reported a complex pattern of changes, including an increase of stage 1 but a decrease of stage 2 sleep in old age, as well as a decrease in REM sleep (6). An epidemiological investigation of self-reported sleep in older adults observed marker sex differences in age-related sleep changes, with females more likely to report disturbed sleep onset but men reporting night-time awakenings (7). Other findings age-related physiological changes in the alignment of homeostatic and circadian rhythms (8), decreases in sleep efficiency (9) the amount of slow-wave sleep, and an increase in daytime napping (10). Importantly, interruption and loss of sleep has been shown to have wide ranging adverse effects on health (11), leaving open the possibility that age-related changes in sleep patterns and quality may contribute to well-documented age-related declines in various health domains.

In the current study, we examine self-reported sleep habits in a large, population-based cohort Cambridge Centre for Ageing and Neuroscience (Cam-CAN (12)). We relate sleep measures to measures of health across four health domains: cognitive, brain health, physical and mental health. Our goal is to quantify and compare the associations between typical age-related changes in sleep quality and a range of measures of health measures that commonly decline in later life. We assess sleep using a self-reported measure of sleep quality, the Pittsburgh Sleep Quality Index (PSQI) (13). The PSQI has good psychometric properties (14) and has been shown to correlate reliably with diseases of aging and mortality (15–17). Although polysomnography (18) is commonly considered the gold standard of sleep quality measurement, it is often prohibitively challenging to employ in

For peer review only - http://bmjopen.bmj.com/site/about/guidelines.xhtml

large samples. A recent direct comparison of sleep measures (19) suggests that although subjective sleep measures (such as PSQI) may have certain drawbacks in older samples, they also capture complementary aspects of sleep quality not fully captured by polysomnography. Moreover, collecting self-report sleep quality data in a large, deeply phenotyped cohort offers several additional benefits.

By utilising a population cohort of healthy adults, and studying a range of health outcomes in the same population, we can circumvent challenges associated with studying clinical populations and provide new insights. First and foremost, by investigating associations between sleep and outcomes across multiple health domains in the same sample, we can make direct comparisons of the relative magnitude of these effects. Second, larger samples allow us to can generate precise effect size estimates, as well as adduce in favour of the null hypothesis. Third, we investigate the associations between sleep quality and neural health in a uniquely large healthy population. Previous investigations of the consequences of poor sleep on especially neural health have generally focuses on clinical populations such as those suffering from insomnia (20,21). Although such studies are crucial for understanding pathology, the demographic idiosyncrasies and often modest sample sizes of these approaches make it hard to generalize to healthy, community dwelling lifespan populations. Moreover, most studies that study age-related changes or differences focus on (very) old age, while far less is known about young and middle aged adults (6). For these reasons, our focus on a healthy, multimodal lifespan cohort is likely to yield novel insights into the subtle changes in sleep quality across the lifespan.

We will focus on three questions within each health domain: First, is there a relationship between sleep quality and health? Second, does the strength and nature of this relationship change when age is included as a covariate? Third, does the strength and nature of the relationship change across the lifespan? We will examine these questions across each of the four health domains.

For peer review only

For peer review only - http://bmjopen.bmj.com/site/about/guidelines.xhtml

METHODS

Sample

A cohort of 2544 (12) was recruited as part of the population-based Cambridge Centre for Ageing and Neuroscience (Cam-CAN) cohort (www.cam-can.com), drawn from the general population via Primary Care Trust (PCT)'s lists within the Cambridge City (UK) area 10,520 invitation letters were sent between 2010 and 2012, and willing participants were invited to have an interview conducted in their home, with questions on health, lifestyle demographics and core cognitive assessments. Sample size was chosen to allow for 100 participants per decile in further acquisition stages, giving sufficient power to separate age-related change from other sources of individual variation. For additional details of the project protocol see (12,22) and for further details of the Cam-CAN dataset visit http://www.mrc-cbu.cam.ac.uk/datasets/camcan/. A further subset of participants who were MRI compatible with no serious cognitive impairment participated in a neuroimaging session (22) between the 2011 and 2013. Participants included were native English speakers, had normal or corrected to normal vision and hearing, scored 25 or higher on the mini mental state (23). Note that other, more stringent cut-offs are sometimes employed to screen for premorbid dementia, such as a score of 88 or higher in the Addenbrookes Cognitive Examination – Revised (24). For the sake of comprehensiveness we repeated our analyses using this more stringent cut off (ACE-R>88), but observed no noteworthy differences in our findings, so we only report the findings based on the MMSE. Ethical approval for the study was obtained from the Cambridgeshire 2 (now East of England-Cambridge Central) Research Ethics Committee (reference: 10/H0308/50). Participants gave written informed consent. The raw data and analysis code are available upon signing a data sharing request form (see http://www.mrc-cbu.cam.ac.uk/datasets/camcan/ for more detail).

Variables

Sleep Measures

For peer review only - http://bmjopen.bmj.com/site/about/guidelines.xhtml

132 Sleep quality was assessed using the Pittsburgh Sleep Quality Index (PSQI), a well-validated self-report questionnaire (13,19) designed to assist in the diagnosis of sleep disorders. The questions concern sleep patterns, habits, and lifestyle questions, grouped into seven components, each yielding a score ranging from 0 (good sleep/no problems) to 3 (poor sleep/severe problems), that are commonly summed to a PSQI Total score ranging between 0 and 21, with higher scores reflecting poorer sleep quality.

Health Measures

Cognitive health. A number of studies have found associations between poor sleep and cognitive decline, including in elderly populations. Poor sleep affects cognitive abilities such as executive functions (25) and learning and memory processes (26), whereas short term pharmaceutical interventions such as administration of melatonin improve both sleep quality and cognitive performance (27,28). Recent work (29) concluded that “maintaining good sleep quality, at least in young adulthood and middle age, promotes better cognitive functioning and serves to protect against age-related cognitive declines”. As sleep may affect various aspects of cognition differently (30), we include measures that cover a range of cognitive domains including memory, reasoning, response speed, and verbal fluency, as well as including a measure of general cognition (See Table 1 and (12) for more details).

Neural health. Previous research suggests that individuals with a severe disruption of sleep are significantly more likely to exhibit signs of poor neural health (20,31). Specifically, previous studies have observed decreased white matter health in clinical populations suffering from conditions such as chronic insomnia (21), obstructive sleep apnoea (32,33), excessively long sleep in patients with diabetes (34), and REM Sleep Behaviour Disorder (35). Many of these studies focus on white matter hyperintensities (WMH), a measure of the total volume or number of (regions) showing low-level neural pathology (although some study grey matter, e.g. (36,37). White matter hyperintensities are often used as a clinical marker, as longitudinal increases in WMHs are associated with increased risk of stroke, dementia and death (38) and are more prevalent in patients

For peer review only - http://bmjopen.bmj.com/site/about/guidelines.xhtml

with pathological sleep problems (33,34). However, use of this metric in clinical cohorts largely leaves open the question of the impact of sleep quality on neural (white matter) health in non-clinical, healthy populations. To address this question, we use a more general indicator of white matter neural health; *Fractional Anisotropy* (FA). FA is associated with white matter integrity and myelination (39,40). We use FA as recent evidence suggests that WMHs represent the extremes (foci) of white matter damage, and that FA is able to capture the full continuum of white matter integrity (41). For more information regarding the precise white matter pipeline, see (12,22,42).

Physical health. Sleep quality is also an important marker for physical health, with poorer sleep being associated with conditions such as obesity, diabetes mellitus (43), overall health (11,44) and increased all-cause mortality (45,46). We focus on a set of variables that capture three types of health domains commonly associated with poor sleep: Cardiovascular health measured by pulse, systolic and diastolic blood pressure (47), self-reported health, both in general and for the past 12 months (48) and body-mass index (49).

Mental health. Previous work has found that disruptions of sleep quality are a central symptom of forms of psychopathology such as Major Depressive Disorder, including both hypersomnia and insomnia (44,50), and episodes of insomnia earlier greatly increased the risk of later episodes of major depression (51). Kaneita et al. (52) found a U-shaped association between sleep and depression, such that individuals regularly sleeping less than 6, or more than 8, hours were more likely to be depressed. Both depression (53) and anxiety (54,55) are commonly associated with sleep problems. To capture these dimensions we used both scales of the Hospital Anxiety and Depression Scale (HADS) (56), a widely used and standardized questionnaire that captures self-reported frequency and intensity of anxiety and depression symptoms.

Health	Task and Description	Variable	Descriptives	Citati

For peer review only

For peer review only - http://bmjopen.bmj.com/site/about/guidelines.xhtml

domain				on
Cognitive	Story Recall Immediate: Participants hear a short story and are asked to recall as accurately as possible.	Recall manually scored for similarity and precision (min=0, max=24)	N = 2379, M=13.14, SD=4.66, Range=(0-24)	(57)
Cognitive	Story Recall Delayed: Same as above but recall after 30 minute delay	Recall manually scored for similarity and precision (min=0, max=24)	N = 2366, M=11.47, SD=4.92, Range=(0-24)	(57)
Cognitive	Letter Fluency (phonemic fluency): Participants have one minute to generate as many words as possible beginning with the letter 'p'.	Total words generated (min=0,max=30)	N = 2360, M=25.38, SD=3.96, Range=(0-30)	(57)
Cognitive	Animal Fluency (semantic fluency): Participants have one minute to generate as many words as possible in the category 'animals'.	Total words generated (min=0,max=30)	N = 2346, M=25.85, SD=4.47, Range=(0-30)	(57)
Cognitive	Cattell Culture Fair: Test of fluid reasoning using four subtests (series completions, odd-one-out, matrices and topology)	Total correct summed across four subtests. Min=0, max=46	N = 658, M=31.8, SD=6.79, Range=(11-44)	(58)
Cognitive	Simple reaction time: Speed in a simple reaction time task	1/response time in seconds	N = 657, M=0.37, SD=0.08, Range=(0.24-0.93)	(12)
Cognitive	Addenbrookes Cognitive Examination, Revised: Screening test for dementia using seven subtests (orientation, attention and concentration, memory, fluency, language, visuospatial abilities, perceptual abilities)	Performance on multiple tests converted to min=0, max=100 range	N = 2406, M=89.25, SD=13.4, Range=(0-100)	(24)
Neural	White matter health: Measure of tract integrity using fractional anisotropy	Fractional Anisotropy (min=0, max=1, averaged across 10 tracts)	N = 641, M=0.5, SD=0.03, Range=(0.3-0.56)	(59)
Physical	Self-reported Health, in general: Participants use a 4-point scale to respond to the prompt "Would you say for someone of your age, your own health in general is..."	Score from 1 = Excellent to 4= Poor	N = 2404, M=2.02, SD=0.79, Range=(1-3)	(60)
Physical	Self-reported Health, last 12 months: Participants use a 3-point scale to respond to the prompt "Over the last twelve months would you say your health has on the whole been..."	Score from 1 = Good to 3= Poor	N = 2398, M=1.46, SD=0.71, Range=(1-3)	(60)

For peer review only - http://bmjopen.bmj.com/site/about/guidelines.xhtml

Physical	Systolic blood pressure	Mean systolic blood pressure in mmHg, averaged across three consecutive measurements	N = 577, M=120.11, SD=17, Range=(78.5-186)	
Physical	Diastolic blood pressure	Mean diastolic blood pressure in mmHg, averaged across three consecutive measurements	N = 577, M=73.14, SD=10.48, Range=(49-115.5)	
Physical	Resting pulse	Mean pulse in beats per minute, averaged across three consecutive measurements	N = 578, M=65.69, SD=10.5, Range=(40-110.5)	
Physical	Body Mass Index (BMI)	(weight in kg) / (height in m)^2	N = 584, M=25.77, SD=4.59, Range=(16.75-48.32)	(61)
Mental health	Anxiety Subscale (Hospital Anxiety and Depression Scale (HADS)): Participants response to seven questions about anxiety-related behaviours	Seven questions rated on 0 to 3 scale ('Often' to 'Very seldom'). Min=0, Max=21	N = 2393, M=5.17, SD=3.4, Range=(0-19)	(56)
Mental health	Depression Subscale (Hospital Anxiety and Depression Scale (HADS)): Participants response to seven questions about depression-related behaviours	Seven questions rated on 0 to 3 scale ('Often' to 'Very seldom'). Min=0, Max=21	N = 2373, M=3.32, SD=2.91, Range=(0-14)	

Table 1. Description of health variables across each of four domains (cognitive, neural, physical, mental). For each variable details are given including a description of the task it is derived from, relevant citations, a brief definition and descriptive statistics.

For peer review only - http://bmjopen.bmj.com/site/about/guidelines.xhtml

STATISTICAL ANALYSES

We examine whether self-reported sleep patterns change across the lifespan, both for the PSQI sum score and for each of the seven PSQI components. We then examine the relationships between the sleep quality and the four health domains in three ways: First, simple regression of the health outcome on sleep variables, to determine evidence for association between poor sleep quality and poor health outcomes. Second, we include age as a covariate. Finally, we include a (standard normal rescaled) continuous interaction term to examine whether there is evidence for a changing relationship between sleep and outcomes across the lifespan.

For all regressions we will use a default Bayesian approach advocated by (62–65) which avoids several well-documented issues with p-values (64), allows for quantification of null effects, and decreases the risk of multiple comparison problems (66). Bayesian regressions allows us to symmetrically quantify evidence in favour of, or against, some substantive model as compared to a baseline (e.g. null) model. This evidentiary strength is expressed as a Bayes Factor (67), which can be interpreted as the relative likelihood of one model versus another given the data and a certain prior expectation. A Bayes Factor of, e.g., 7, in favour of a regression model suggests that the data are seven times *more likely* under that model than an intercept only model for a given prior (for an empirical comparison of p-values and Bayes factors, see (65)). A heuristic summary of evidentiary interpretation can be seen in Figure 1.

[insert Figure 1 here]

We report log Bayes Factors for (very) large effects and regular Bayes Factors for smaller effects. To compute Bayes Factors we will use Default Bayes Factor approach for model selection (62,63) in the package BayesFactor (68) using the open source software package R (69). As previous papers report associations between sleep and outcomes ranging from absent to considerable in size we utilize the default, symmetric Cauchy prior with width $\frac{\sqrt{2}}{2}$ which translates to a 50% confidence that the true effect will lie between -.707 and .707. Prior to further analysis, scores on all outcomes were transformed to a standard normal distribution, and any scores exceeding a z-score of 4 or -4

For peer review only

For peer review only - http://bmjopen.bmj.com/site/about/guidelines.xhtml

were recoded as missing (aggregate percentage outliers across the four health domains: Cognitive, 0.41%, Mental, 0.16%, Neural, 0.37% Physical, 0.031%).

RESULTS

Age-related differences in sleep quality

First, we examined sleep changes across the lifespan by examining age-related differences in the PSQI sum score (N= 2178, M=5.16, SD=3.35, Range=0-19). Regressing the PSQI global score on age, (see Supplementary Figure 1) showed evidence for a positive relationship across the lifespan ($logBF_{10}$= 10.45). This suggests that on the whole, sleep quality decreases across the lifespan (note that *higher* PSQI scores correspond to worse sleep). Although we observe strong statistical evidence for an age-related difference ('Extreme' according to (70)) age explained only 1.23 % of the variance in the PSQI Total score. Next, we examined each of the seven components on age in the same manner. In Supplementary Figure 2 we see that that age has varying and specific effects on different aspects of sleep quality, and did not worsen uniformly across the lifespan. For example, we observed moderate evidence that sleep latency did not change across the lifespan (Sleep Latency, BF_{01}= 9.25, in favour of the null), Sleep Quality showed no evidence for either change or stasis (BF_{10}= 1.63) and one sleep component, Daytime Dysfunction, improved slightly across the lifespan (BF_{10}= 7.03). Medication). The strongest age-related decline is that of Efficiency, showing an R-squared of 6.6%.

Finally, we entered all seven components into a Bayesian multiple regression simultaneously, to examine to what extent they could, together, predict age. The best model included every component except Sleep Latency ($logBF_{10}$= 142.71). Interestingly, this model explained 13.66% of the variance in age, compared to 1.23% for the PSQI Total score, and 6.6% for the strongest single component (efficiency). This shows that lifespan changes in self-reported sleep are heterogeneous and partially independent, and that specific patterns and components need to be taken into account simultaneously to fully understand age-related differences in sleep quality. These

For peer review only - http://bmjopen.bmj.com/site/about/guidelines.xhtml

finding shows that neither the PSQI sum score nor the sleep components in isolation fully capture differences in sleep quality across the lifespan.

The analysis above suggests that conceptualizing 'poor sleep' as a single dimension does not reflect the subtleties in lifespan changes – An often computed sumscore changes little across the lifespan, whereas the totality of sleep symptoms shows far stronger, and more subtle, patterns. To better elucidate individual differences in sleep quality we next use *Latent Class Analysis* (71). This technique will allow us examine individual differences in sleep quality across the lifespan in more detail than afforded by simple linear regressions: Rather than examining continuous variation in sleep components, LCA classifies individuals into different *sleep types*, each associated with a distinct profile of 'sleep symptoms'. If there are specific constellations of sleep problems across individuals, we can quantify and visualize such sleep types.

To analyse the data in this manner, we binarized the responses on each component into 'good' (0 or 1) or 'poor' (2 or 3). Our measures of PSQI symptoms straddle the border between continuous and categorical – Although some are fully continuous (e.g. sleep latency) others are less so. For instance, although scored on a range of four several of the scales (such as Subjective Sleep quality) have implicitly binary response options of 'Very good' and 'fairly good' on the one hand and 'fairly bad' and 'very bad' on the other. As analytical work in psychometrics (72) suggests that likert-like graded scales can be treated as continuous only from five ordinal categories upwards, by fitting an LCA we are erring on the side of caution (although a latent profile analysis would likely give similar results). Note that although our analysis divides individuals into discrete classes with specific profiles, it is still possible to examine the conditional response likelihood of responding 'yes' to each symptom as a continuous metric (between 0 and 1) that reflects the nature of the association between the class and the outcome. By modelling sleep 'types' we hope to illustrate the complex patterns in a more intelligible manner – notably, doing so allows us to examine whether the likelihood of belonging to any sleep 'type' changes as a function of age.

For peer review only - http://bmjopen.bmj.com/site/about/guidelines.xhtml

Next we examined evidence for distinct sleep types using We fit a set of possible models (varying from 2 to 6 sleep types) We found that the four class solution gives the best solution, according to the Bayesian Information Criterion (73) (BIC for 4 Classes = 11825.65, lowest BIC for other solutions= 11884.92 (5 classes) (with 50 repetitions per class, at 5000 maximum iterations). Next we inspected the nature of the sleep types, the prevalence of each 'sleep type' in the population, and whether the likelihood of belonging to a certain sleep type changes across the lifespan. See Figure 2 for the component profiles of the four sleep types identified.

[insert Figure 2 here]

Class 1, 'Good sleepers', make up 68.1% of participants. Their sleep profile is shown in Figure 2A, top left, and is characterised by a low probability of responding 'poor' to any of the sleep components. Class 2, 'inefficient sleepers', make up 14.01% of the participants, and are characterized by poor sleep Efficiency: Members of this group uniformly (100%) report poor sleep Efficiency, despite relatively low prevalence of other sleep problems, as seen in Figure 2A, top right. Class 3, 'Delayed Sleepers' seen in the bottom left of Figure 2a, makes up 9.28% of the participants: characterized by modestly poor sleep across the board, but a relatively high probability of poor scores on Sleep Latency (59%), Sleep Quality (51%) and sleep Disturbance (31%). Finally, Class 4, 'Poor sleepers', make up 8.5% of the participants, shown bottom right in Figure 2A. Their responses to any of the seven sleep components are likely to be 'poor' or 'very poor', almost universally so for 'sleep quality' (94%) and 'Sleep Efficiency' (97.7%).

Next, we including age as a covariate (simultaneously including a covariate is known as *latent class regression* or concomitant-variable latent class models (74). This analysis, visualised in Figure 2b, shows that the probability of membership of each classes compared to the reference class (good sleepers) changes significantly across the lifespan for each of the classes (Class 2 versus class 1: beta/SE= 0.05/0.00681, t=7.611, Class 3 versus class 1: beta/SE= -0.01948/0.0055, t=-3.54), Class 4 versus class 1: beta/SE 0.01269/0.00478, t=2.655, for more details on generalized logit coefficients , see (71). The frequency of Class 1 (Good sleepers) peaks in middle to late adulthood, dropping

For peer review only - http://bmjopen.bmj.com/site/about/guidelines.xhtml

increasingly quickly after age 50. Class 2 (Inefficient sleepers) are relatively rare in younger individuals, but the prevalence increases rapidly in individuals over age 50. On the other hand, Class 3 (Delayed sleepers) shows a steady decrease in the probability of an individual showing this profile across the lifespan, suggesting that this specific pattern of poor sleep is more commonly associated with younger adults. Finally, the proportion of Class 4 (poor sleepers) members increases only slightly across the lifespan. Together, the latent class analysis provides additional evidence that the PSQI sum score as an indicator of sleep quality does not fully capture the subtleties of age-related differences. Age-related changes in sleep patterns are characterized by specific, clustered patterns of sleep problems that cannot be adequately characterized by summation of the component scores.

The above analyses show how both a summary measure and individual measures of sleep quality change across the lifespan. Next, we examined the relationships between sleep quality measures (seven components and the global PSQI score) and health variables (specific variables across four domains, as shown in Table 1).

Sleep, health domains and age

Cognitive health

First, we examined the relationships between sleep quality and seven measures of cognitive health (see Table 1 for details). We visualize our findings using tileplots (75). Each cell shows the numeric effect size (R-squared, 0-100) of the bivariate association between a sleep component and a health outcome, colour coded by the statistical evidence for a relationship using the Bayes Factor. If the parameter estimate is positive, the r-squared value has the symbol '+' added (note the interpretation depends on the nature of the variable, cf. Table 1).

As can be seen in Supplementary Figure 3, several relationships exist between measures of cognitive health and measures of sleep quality. However, these results attenuate in a multiple regression model including age as shown in Figure 3.

[Insert Figure 3 here]

For peer review only - http://bmjopen.bmj.com/site/about/guidelines.xhtml

The cognitive abilities most strongly associated with poor sleep are a measure of general cognitive health, ACE-R, and a test of verbal phonemic fluency. Two patterns emerged: First, the strongest predictor across the simple and multiple regressions was for the PSQI Total score. Tentatively this suggests that a cumulative index of sleep problems, rather than any specific pattern of poor sleep, is the biggest risk factor for poorer cognitive performance. Secondly, after controlling for age, the most strongly affected cognitive measure is phonemic fluency, the ability to generate name as many different words as possible starting with a given letter within a minute. Verbal fluency is commonly used as a neuropsychological test (76). Previous work suggests it depends on both the ability to cluster (generating words within a semantic cluster) and to switch (switching between categories), and is especially vulnerable to frontal and temporal lobe damage (with specific regions dependant on either a semantic or phonemic task (77)). Although modest in size, our findings suggests this task, dependent on multiple executive processes, is particularly affected by poor sleep quality (78). The second strongest association was with the ACE-R, a general cognitive test battery similar in style and content to the MMSE. When an interaction term with age was included, little evidence for interactions with age (mean $logBF_{10}$=-2.08, see Supplementary Figure 4), suggesting that the negative associations between sleep and cognitive performance are a constant feature across the lifespan, rather than specifically in elderly individuals. Together this suggests that poor sleep quality is modestly but consistently associated with poorer general cognitive performance across the lifespan, most strongly with semantic fluency.

Neural Health

Using Diffusion Tensor Imaging, we estimated a general index of white matter integrity in 10 tracts (59) (shown in Supplementary Figure 5), by taking the average Fractional Anisotropy in each white matter ROI (see (79) for more information). We use the data from a subsample of 641 individuals (age M=54.87, range 18.48-88.96) who were scanned in a 3T MRI scanner (for more details regarding the pipeline, sequence and processing steps, see (22,79). Regressing neural WM ROI's on sleep

For peer review only

For peer review only - http://bmjopen.bmj.com/site/about/guidelines.xhtml

quality, we find several small effects, with the strongest associations between sleep efficiency and neural health (see Supplementary Figure 6). All effects are such that poorer sleep is associated with poorer neural health, apart from a small effect in the opposite direction for Uncinate and Daytime Dysfunction (BF_{10}= 6.20). However, when age is included as a covariate, the negative associations between sleep quality and white matter health are attenuated virtually to zero (Figure 4, mean/median BF_{10}= 0.18/.10), with Bayes Factors providing strong evidence for the lack of associations between sleep quality and white matter integrity. One exception was observed: The use of Sleep Medication is associated with *better* neural health in the corticospinal tract, a region previously found to be affected by pathological sleep problems such as sleep apnoea (33). However, this effect is very small (BF_{10}=3.24) given the magnitude of the sample and the range of comparisons, so should be interpreted with caution.

[Insert Figure 4 here]

Finally, we tested for any interactions by including a mean-scaled interaction term (sleep*age, Supplementary Figure 7). This analysis found evidence for a significant interaction, between the Superior Longitudinal Fasciculus (SLF) and Sleep Medication (BF_{10}= 13.77), such that better neural health in the SLF was associated with the use of Sleep Medication more strongly in older adults. Together, these findings suggest that in general, once age is taken into account, self-reported sleep problems in a non-clinical sample are *not* associated with poorer neural health, although there is some evidence for a modest associations between better neural health in specific tracts and the use of sleep medication in the elderly.

Physical health

Next we examined whether sleep quality is associated with physical health. Figure 5 shows the simple regressions between sleep quality and physical health. Strong associations were found between poor overall sleep (PSQI sum score) and poor self-reported health, both in general ($logBF_{10}$=77.51) and even more strongly for health in the past 12 months ($logBF_{10}$=91.25). This may

For peer review only - http://bmjopen.bmj.com/site/about/guidelines.xhtml

be because poorer sleep, across all components, directly affects general physical health (43,80) or because people subjectively experience sleep quality as a fundamental part of overall general health. A second association was between BMI and poor sleep quality, most strongly poor Duration ($logBF_{10}$=4.69).

[Insert Figure 5 here]

This not only replicates previous findings but is in line with an increasing body of evidence that suggests that shorted sleep duration causes metabolic changes, which in turn increases the risk of both diabetes mellitus and obesity (43,81,82). Next, we examined whether these effects were attenuated once age was included. We show that although the relationships are slightly weaker, the overall pattern remains (Supplementary Figure 8), suggesting these associations are not merely co-occurences across the lifespan. Our findings suggest self-reported sleep quality, especially sleep Duration, is related to differences in physical health outcomes in a healthy sample.

Finally, there was evidence of a single interaction with age (Supplementary Figure 9): Although poor sleep Duration was associated with *higher* diastolic blood pressure in younger adults, it was associated with *lower* diastolic blood pressure in older individuals (BF_{10}= 8.53). This may reflect the fact that diastolic blood pressure is related to cardiovascular health in a different way across the lifespan, although given the small effect size it should be interpreted with caution.

Mental health

Finally, we examined the relationship between sleep quality and mental health, as measured by the Hospital Anxiety and Depression Scale (56). One benefit of the HADS in this context is that, unlike some other definitions (e.g. the DSM-V), sleep quality is not an integral (scored) symptom of these dimensions. As shown in Supplementary Figure 10, there are very strong relationships between all aspects of sleep quality and measures of both anxiety and depression. The strongest predictors of Depression are Daytime Dysfunction ($logBF_{10}$= 245.9, R^2=20.9%), followed by the overall sleep score ($logBF_{10}$= 170.5, R^2=14.6%) and sleep quality ($logBF_{10}$= 106.8, R^2=9.7%). The effects size for

For peer review only - http://bmjopen.bmj.com/site/about/guidelines.xhtml

Anxiety was comparable but slightly smaller in magnitude. When age is included as a covariate the relationships remained virtually unchanged (Supplementary Figure 11), suggesting these relationships are present throughout across the lifespan. These findings replicate and extend previous work, suggesting that sleep quality is strongly associated with both anxiety and depression across the lifespan.

Finally we examined a model with an interaction term (Supplementary Figure 12). Most prominently we found interactions with age in the relationship between HADS depression and the PSQI Total, and in the relationship between HADS depression and Sleep Duration, such that for the relationship between anxiety and overall sleep quality is stronger in younger adults (BF_{10} =9.91, see Figure 6). Together our findings show that poor sleep quality is consistently, strongly and stably associated with poorer mental health across the adult lifespan.

[Insert Figure 6 here]

Non-linear associations between sleep and health outcomes

In the above analyses, we focused on linear associations between symptoms and health outcomes. However, for one aspect of sleep, namely sleep duration (in hours), evidence exists that these associations are likely to be non-linear, such that both shorter and longer than average sleep are associated with poorer health outcomes (e.g. (83–85). This is echoed in clinical criteria for depression, which commonly include that include both hyper- and hypo-somnia as 'sleep disruption' symptoms – In other words, both too much or too little sleep are suboptimal. To examine whether we observe evidence for non-linearities we examined the relationship between raw scores on sleep duration (in hours, not transformed to PSQI norms) and health outcomes across the four domains. If the association between sleep and outcomes is indeed u-shaped (or inverted U, depending on the scale) then a Bayesian regression would prefer the less parsimonious model that includes the quadratic term. We observed no non-linear associations between any neural or cognitive health variables. We find strong evidence for a quadratic (subscript q) over a linear (subscript l) associations

For peer review only - http://bmjopen.bmj.com/site/about/guidelines.xhtml

between sleep duration and HADS anxiety ($logBF_{ql}$= 19.98), even more strongly so with HADS Depression ($logBF_{ql}$= 25.83, see Figure 7A shows the strongest curvilinear association, namely with depression). We find a similar u-shaped curve with general health (BF_{ql}= 277.81) and self-reported health over the last 12 months (BF_{ql}=887.59), the latter shown in Figure 7b. Together, these analyses support previous conclusions that some (although not all) poorer health outcomes can be associated with both too much and too little sleep.

[Insert Figure 7 here]

DISCUSSION

In this study, we report on the associations between age-related differences in sleep quality and health outcomes in a large, age-heterogeneous sample of community dwelling adults of the Cambridge Neuroscience and Aging (Cam-CAN) cohort. We find that sleep quality generally decreases across the lifespan, most strongly for sleep Efficiency. However age-related changes in sleep patterns are complex and multifaceted, so we used Latent Class Analysis to identify 'sleep types' associated with specific sleep quality profiles. We found that Younger adults are more likely than older adults to display a pattern of sleep problems characterised by poor sleep quality and longer sleep latency, whereas older adults are more likely to display inefficient sleeping, characterised by long periods spent in bed whilst not asleep. Moreover, the probability of being a 'good' sleeper, unaffected by any adverse sleep symptoms, decreases considerably after age fifty.

Notably, closer investigation of the sleep classes reveals likely further complexities of age-related differences. The category 'poor sleepers', most prevalent in older adults, shows high conditional likelihood of 'poor sleep' across all symptoms except 'daytime dysfunction'. One possible explanation is that almost all individuals in this group are beyond retirement age. For this reason, they likely have greater flexibility in tailoring their day to day activities to their energy levels (as opposed to individuals working fulltime), and are therefore less likely to consider themselves 'disrupted' even in the presence of suboptimal sleep. Although more detailed, interview-based

For peer review only - http://bmjopen.bmj.com/site/about/guidelines.xhtml

investigations would be necessary to examine the precise nature of these findings, it stands to reason that certain symptoms change not just in prevalence but also in meaning across the lifespan.

One key strength of our broad phenotypic assessment allows for direct comparison of the different measures of sleep quality and four key health domains. We find strongest associations between sleep quality and mental health, moderate relations between sleep quality and physical health and cognitive health and sleep, virtually all such that poorer sleep is associated with poorer health outcomes. We did not find evidence for associations between self-reported sleep and neural health. Notably, the relationships we observe are mostly stable across the lifespan, affecting younger and older individuals alike. A notable exception to these effects is the absence of any strong relation (after controlling for age) between sleep quality and neural health as indexed by tract-based average fractional anisotropy. Perhaps surprisingly, given we found strong relationships in the same sample between sleep and other outcomes (e.g. mental health, Figure 10) we find that self-reported sleep problems in a non-clinical sample are not associated with fractional anisotropy above and beyond old age. This is despite the fact that previous work within the same cohort observed moderate to strong associations between white matter and various cognitive outcomes (42,86,87). However, although notable, our finding does not rule out that such associations do exist with other white matter metrics, that they would be observed with objective measures of sleep such as polysomnography, or that the co-occurrence of age-related declines in sleep quality and white matter share an underlying causal association that cannot be teased apart in a cross-sectional sample.

One strength of our study is the assessment of neuroimaging metrics, namely fractional anisotropy, in a large, community-dwelling healthy population. Fractional anisotropy is often used in studies of aging (e.g. Madden, is relatively reliable (88)) and is sensitive to clinical anomalies such as white matter hyperintensities. However, the relationship between FA and white-matter health is indirect (40,89) and drawbacks include its inability to distinguish crossing fibers (e.g. (40,89) and vulnerability to movement and the fact that it likely reflects a combination of underlying

For peer review only - http://bmjopen.bmj.com/site/about/guidelines.xhtml

physiological properties. Various alternative white matter metrics exist, including summary measures of diffusivity (e.g. axial/radial/mean diffusivity), volumetric measures of white matter hyperintensity (e.g.) and various innovative measures currently in development, but their physiological validity is ongoing (89,90).

While there are limitations of self-report measures including in older cohorts (19), including the fact that they likely reflect different aspects of sleep health than polysomnography (sleep in the lab), our results suggest there are considerable advantages in using self-reported sleep measures: first, obtaining sleep quality data in a large and broadly phenotyped sample is feasible; and second, our results demonstrated clear and consistent associations across multiple domains for both subjective (e.g. self-reported health) and objective measures (e.g. memory tests, BMI), which both replicate and extend previous lab-based sleep findings. Future work should ideally simultaneously measure polysomnography and self-report in longitudinal, large scale cohorts to fully capture the range of overlapping and complementary relations between different aspects of sleep quality and health outcomes (19).

For both self-report and objective measures of sleep quality an open question is that of causality: Does poor sleep affect health outcomes, do health problems affect sleep, are they both markers of some third problem, or do causal influences go both ways? Most likely, all these patterns occur to varying degrees. Previous studies have shown that sleep quality causally affects health outcomes such as diabetes (43) and memory consolidation (1) while other evidence suggests that depression directly affect sleep quality (91,92) and that damage to neural structures may affect sleep regulation (93). Although our findings are in keeping with previous findings, our cross-sectional sample cannot tease apart the causal direction of the observed associations, more work remains to be done to disentangle these complex causal pathways.

In our paper we focus on a healthy, age-heterogeneous community dwelling sample. This allows us to study the associations between healthy aging and self-reported sleep quality, but comes with two key limitations of the interpretations of our findings. First and foremost, our findings are

For peer review only - http://bmjopen.bmj.com/site/about/guidelines.xhtml

cross-sectional, not longitudinal. This means we can make inferences about age-related *differences*, but not necessarily age-related *changes* (94,95). One reason why cross-sectional and longitudinal estimates may diverge is that older adults can be thought of as cohorts that differ from the younger adults in more ways than age alone. For example, our age range includes individuals born in the twenties and thirties of the 20th century. Compared to someone born in the 21st century, these individuals will likely have experience various differences during early life development (e.g. less broadly accessible education, lower quality of healthcare, poorer nutrition and similar patterns). For some of our measures, these are inherent limitations –*truly* longitudinal study of neural aging is inherently impossible as scanner technology has not been around sufficiently long. This means our findings likely reflect a combination of effects attributable to age-related changes as well as baseline differences between subpopulations that may affect both mean differences as well as developmental trajectories.

Second, our sample reflects an atypical population in the sense that they are willing and able to visit the laboratory on multiple occasions for testing sessions. This subsample is likely a more healthy subset of the full population, which will mean the range of (poor) sleep quality as well as (poorer) health outcomes will likely be less extreme that in the full population. However, this challenge is not specific to our sample. In fact, as the Cam-CAN cohort was developed using stratified sampling based on primary healthcare providers, our sample is likely as population-representative as is feasible for a cohort of this magnitude and phenotypic breadth (see (12) for further details). Nonetheless, a healthier subsample may lead to restriction of range (96), i.e. an attenuation of the strength of the associations observed between sleep quality and health outcomes. Practically, this means that our results likely generalise to comparable, healthy community dwelling adults, but not necessarily to populations that include those affected by either clinical sleep deprivation or other serious health conditions.

For peer review only - http://bmjopen.bmj.com/site/about/guidelines.xhtml

Conclusions

Taken together, our study allows several conclusions. First, although we replicate the age-related deterioration in some aspects of sleep quality, other aspects remain stable or even improve. Second, we show that the profile of sleep quality changes across the lifespan. This is important methodologically, as it suggests that PSQI sum scores do not capture the full picture, especially in age-heterogeneous samples. Moreover, it is important from a psychological standpoint: We show that ‘sleep quality’ is a multidimensional construct and should be treated as such if we wish to understand the complex effects and consequences of sleep quality across the lifespan. Third, moderate to strong relations exist between sleep quality and cognitive, physical and mental health, and these relations largely remain stable across the lifespan. In contrast, we show evidence that in non-clinical populations, poorer self-reported sleep is not reliably associated with poorer neural health. Finally, we find that for absolute sleep duration, we replicate previous findings that both longer and shorter than average amounts of sleep are association with poorer self-reported general health and higher levels of depression and anxiety.

Together with previous experimental and longitudinal evidence, our findings suggest that at least some age-related decreases in health outcomes may be due to poorer sleep quality. We show that self-reported sleep quality can be an important indicator of other aspects of healthy functioning throughout the lifespan, especially for mental and general physical health. Our findings suggest accurate understanding of sleep quality is essential in understanding and supporting healthy aging across the lifespan.

For peer review only - http://bmjopen.bmj.com/site/about/guidelines.xhtml

Author contributions

AG, MS and MS designed the study. AG and RAK performed the analyses. CC organized and conducted the data collection. AG, MS and RAK wrote the manuscript. YL provided considerable expertise on sleep and poor sleep outcomes. All authors approved the final manuscript.

Acknowledgements

The Cambridge Centre for Ageing and Neuroscience (Cam-CAN) research was supported by the Biotechnology and Biological Sciences Research Council (grant number BB/H008217/1). RAK is supported by the Sir Henry Wellcome Trust (grant number 107392/Z/15/Z) and the by UK Medical Research Council Programme (MC-A060-5PR61).

We would like to thank Richard Morey and Eric-Jan Wagenmakers for valuable suggestions regarding the use of the BayesFactor package. We are grateful to the Cam-CAN respondents and their primary care teams in Cambridge for their participation in this study. We also thank colleagues at the MRC Cognition and Brain Sciences Unit MEG and MRI facilities for their assistance. The Cam-CAN corporate author consists of the project principal personnel: Lorraine K Tyler, Carol Brayne, Edward T Bullmore, Andrew C Calder, Rhodri Cusack, Tim Dalgleish, John Duncan, Richard N Henson, Fiona E Matthews, William D Marslen-Wilson, James B Rowe, Research Associates: Karen Campbell, Teresa Cheung, Simon Davis, Linda Geerligs, Anna McCarrey, Abdur Mustafa, Darren Price, David Samu, Jason R Taylor, Matthias Treder, Kamen Tsvetanov, Janna van Belle, Nitin Williams; Research Assistants: Lauren Bates, Tina Emery, Sharon Erzinçlioglu, Sofia Gerbase, Stanimira Georgieva, Claire Hanley, Beth Parkin, David Troy; Affiliated Personnel: Tibor Auer, Marta Correia, Lu Gao, Emma Green, Rafael Henriques; Research Interviewers: Jodie Allen, Gillian Amery, Liana Amunts, Anne Barcroft, Amanda Castle, Cheryl Dias, Jonathan Dowrick, Melissa Fair, Hayley Fisher, Anna Goulding, Adarsh Grewal, Geoff Hale, Andrew Hilton, Frances Johnson, Patricia Johnston, Thea Kavanagh-Williamson, Magdalena Kwasniewska, Alison McMinn, Kim Norman, Jessica Penrose, Fiona Roby, Diane Rowland, John Sargeant, Maggie Squire, Beth Stevens, Aldabra Stoddart, Cheryl Stone, Tracy

For peer review only - http://bmjopen.bmj.com/site/about/guidelines.xhtml

571 Thompson, Ozlem Yazlik; and administrative staff: Dan Barnes, Marie Dixon, Jaya Hillman, Joanne
572 Mitchell, Laura Villis.

573

For peer review only

For peer review only - http://bmjopen.bmj.com/site/about/guidelines.xhtml

References

1. Stickgold R. Sleep-dependent memory consolidation. Nature [Internet]. 2005 Oct 27 [cited 2014 Jul 10];437(7063):1272–8. Available from: http://dx.doi.org/10.1038/nature04286

2. Inoué S, Honda K, Komoda Y. Sleep as neuronal detoxification and restitution. Behav Brain Res. 1995 Jul;69(1–2):91–6.

3. Xie L, Kang H, Xu Q, Chen MJ, Liao Y, Thiyagarajan M, et al. Sleep drives metabolite clearance from the adult brain. Science [Internet]. NIH Public Access; 2013 Oct 18 [cited 2014 Jul 11];342(6156):373–7. Available from: http://europepmc.org/articles/PMC3880190/?report=abstract

4. D'Ambrosio C, Redline S. Impact of Sleep and Sleep Disturbances on Obesity and Cancer. Redline S, Berger NA, editors. New York, NY: Springer New York; 2014.

5. Crowley K. Sleep and Sleep Disorders in Older Adults. Neuropsychol Rev [Internet]. Springer US; 2011 Mar 12 [cited 2017 Feb 10];21(1):41–53. Available from: http://link.springer.com/10.1007/s11065-010-9154-6

6. Ohayon MM, Carskadon MA, Guilleminault C, Vitiello M V. Meta-analysis of quantitative sleep parameters from childhood to old age in healthy individuals: developing normative sleep values across the human lifespan. Sleep [Internet]. American Academy of Sleep Medicine; 2004 Nov 1 [cited 2017 Feb 10];27(7):1255–73. Available from: http://www.ncbi.nlm.nih.gov/pubmed/15586779

7. Middelkoop HAM, Smilde-van den Doel DA, Neven AK, Kamphuisen HAC, Springer CP. Subjective Sleep Characteristics of 1,485 Males and Females Aged 50-93: Effects of Sex and Age, and Factors Related to Self-Evaluated Quality of Sleep. Journals Gerontol Ser A Biol Sci Med Sci [Internet]. 1996 May 1 [cited 2015 Jun 22];51A(3):M108–15. Available from: http://biomedgerontology.oxfordjournals.org/content/51A/3/M108.short

8. Schmidt C, Peigneux P, Cajochen C. Age-related changes in sleep and circadian rhythms: impact on cognitive performance and underlying neuroanatomical networks. Front Neurol [Internet]. 2012 Jan [cited 2014 Jun 4];3:118. Available from: http://www.pubmedcentral.nih.gov/articlerender.fcgi?artid=3405459&tool=pmcentrez&rendertype=abstract

9. Leng Y, Wainwright NWJ, Cappuccio FP, Surtees PG, Luben R, Wareham N, et al. Self-reported sleep patterns in a British population cohort. Sleep Med [Internet]. 2014 Mar [cited 2016 Jan 28];15(3):295–302. Available from: http://www.sciencedirect.com/science/article/pii/S1389945714000185

10. Stanley N. The physiology of sleep and the impact of ageing. Eur Urol Suppl [Internet]. 2005 Jan [cited 2014 Sep 23];3(6):17–23. Available from: http://www.sciencedirect.com/science/article/pii/S156990560580003X

11. Briones B, Adams N, Strauss M, Rosenberg C, et al. Relationship between sleepiness and general health status. Sleep. 1996;19(7):583–8.

12. Shafto MA, Tyler LK, Dixon M, Taylor JR, Rowe JB, Cusack R, et al. The Cambridge Centre for Ageing and Neuroscience (Cam-CAN) study protocol: a cross-sectional, lifespan, multidisciplinary examination of healthy cognitive ageing. BMC Neurol [Internet]. BioMed Central; 2014 Jan 14 [cited 2015 May 20];14(1):204. Available from: http://bmcneurol.biomedcentral.com/articles/10.1186/s12883-014-0204-1

13. Buysse D, Reynolds C, Monk T, Berman S, Kupfer D. The Pittsburgh Sleep Quality Index: A new

For peer review only

For peer review only - http://bmjopen.bmj.com/site/about/guidelines.xhtml

instrument for Psychiatric Practise and Research .pdf. 1988. p. 193–213.

14. Carpenter JS, Andrykowski MA. Psychometric evaluation of the pittsburgh sleep quality index. J Psychosom Res [Internet]. 1998 Jul [cited 2015 Dec 10];45(1):5–13. Available from: http://www.sciencedirect.com/science/article/pii/S0022399997002985

15. Kang S-H, Yoon I-Y, Lee SD, Kim J-W. The impact of sleep apnoea syndrome on nocturia according to age in men. BJU Int [Internet]. 2012 Dec [cited 2015 Nov 25];110(11 Pt C):E851-6. Available from: http://www.ncbi.nlm.nih.gov/pubmed/22958406

16. Lou P, Qin Y, Zhang P, Chen P, Zhang L, Chang G, et al. Association of sleep quality and quality of life in type 2 diabetes mellitus: a cross-sectional study in China. Diabetes Res Clin Pract [Internet]. 2015 Jan [cited 2015 Nov 25];107(1):69–76. Available from: http://www.sciencedirect.com/science/article/pii/S0168822714004604

17. Mellor A, Waters F, Olaithe M, McGowan H, Bucks RS. Sleep and aging: examining the effect of psychological symptoms and risk of sleep-disordered breathing. Behav Sleep Med [Internet]. Routledge; 2014 Jan 28 [cited 2015 Nov 25];12(3):222–34. Available from: http://www.tandfonline.com/doi/abs/10.1080/15402002.2013.801343#.VlVu9HYrKHs

18. Kushida CA, Littner MR, Morgenthaler T, Alessi CA, Bailey D, Coleman J, et al. Practice Parameters for the Indications for PSG—AASM Practice Parameters Practice Parameters for the Indications for Polysomnography and Related Procedures: An Update for 2005. Sleep. 2005;28(4).

19. Landry GJ, Best JR, Liu-Ambrose T. Measuring sleep quality in older adults: a comparison using subjective and objective methods. Front Aging Neurosci [Internet]. Frontiers; 2015 Sep 7 [cited 2015 Sep 7];7. Available from: http://journal.frontiersin.org/article/10.3389/fnagi.2015.00166/abstract

20. Altena E, Vrenken H, Van der Werf YD, Heuvel OA van den H, Someren EJW van, van den Heuvel OA, et al. Reduced Orbitofrontal and Parietal Gray Matter in Chronic Insomnia: A Voxel-Based Morphometric Study [Internet]. Vol. 67, BIOL PSYCHIATRY. 2010 [cited 2014 Jul 3]. p. 182–185. Available from: http://www.sciencedirect.com/science/article/pii/S0006322309009548

21. Spiegelhalder K, Regen W, Prem M, Baglioni C, Nissen C, Feige B, et al. Reduced anterior internal capsule white matter integrity in primary insomnia. Hum Brain Mapp [Internet]. 2014 Jul 13 [cited 2015 Aug 4];35(7):3431–8. Available from: http://doi.wiley.com/10.1002/hbm.22412

22. Taylor JR, Williams N, Cusack R, Auer T, Shafto MA, Dixon M, et al. The Cambridge Centre for Ageing and Neuroscience (Cam-CAN) data repository: Structural and functional MRI, MEG, and cognitive data from a cross-sectional adult lifespan sample. Neuroimage [Internet]. 2015 Sep 12 [cited 2015 Sep 21]; Available from: http://www.sciencedirect.com/science/article/pii/S1053811915008150

23. Folstein MF, Folstein SE, McHugh PR. “Mini-mental state” a practical method for grading the cognitive state of patients for the clinician. J Psychiatr Res. 1975;12:189–98.

24. Mioshi E, Dawson K, Mitchell J, Arnold R, Hodges JR. The Addenbrooke’s Cognitive Examination Revised (ACE-R): a brief cognitive test battery for dementia screening. Int J Geriatr Psychiatry [Internet]. 2006 Nov [cited 2015 Sep 29];21(11):1078–85. Available from: http://www.ncbi.nlm.nih.gov/pubmed/16977673

25. Regestein QR, Friebely J, Shifren JL, Scharf MB, Wiita B, Carver J, et al. Self-reported sleep in postmenopausal women. Menopause [Internet]. 2004 [cited 2015 Feb 17];11(2):198–207.

For peer review only - http://bmjopen.bmj.com/site/about/guidelines.xhtml

Available from: http://journals.lww.com/menopausejournal/Abstract/2004/11020/Self_reported_sleep_in_postmenopausal_women.12.aspx

26. Curcio G, Ferrara M, De Gennaro L. Sleep loss, learning capacity and academic performance. Sleep Med Rev [Internet]. 2006 Oct [cited 2015 Sep 15];10(5):323–37. Available from: http://www.sciencedirect.com/science/article/pii/S1087079205001231

27. Ferracioli-Oda E, Qawasmi A, Bloch MH, Hossain J, Shapiro C, Dikeos D, et al. Meta-Analysis: Melatonin for the Treatment of Primary Sleep Disorders. Romanovsky AA, editor. PLoS One [Internet]. Public Library of Science; 2013 May 17 [cited 2017 Jan 31];8(5):e63773. Available from: http://dx.plos.org/10.1371/journal.pone.0063773

28. Jean-Louis G, Gizycki H, Zizi F. Melatonin effects on sleep, mood, and cognition in elderly with mild cognitive impairment. J Pineal Res [Internet]. 1998 Nov [cited 2015 Jun 16];25(3):177–83. Available from: http://doi.wiley.com/10.1111/j.1600-079X.1998.tb00557.x

29. Scullin MK, Bliwise DL. Sleep, Cognition, and Normal Aging: Integrating a Half Century of Multidisciplinary Research. Perspect Psychol Sci. 2015 Jan;10(1):97–137.

30. Nebes RD, Buysse DJ, Halligan EM, Houck PR, Monk TH. Self-reported sleep quality predicts poor cognitive performance in healthy older adults. J Gerontol B Psychol Sci Soc Sci [Internet]. 2009 Mar [cited 2014 Sep 8];64(2):180–7. Available from: http://www.pubmedcentral.nih.gov/articlerender.fcgi?artid=2655169&tool=pmcentrez&rendertype=abstract

31. Kamba M, Inoue Y, Higami S, Suto Y, Ogawa T, Chen W. Cerebral metabolic impairment in patients with obstructive sleep apnoea: an independent association of obstructive sleep apnoea with white matter change. J Neurol Neurosurg Psychiatry [Internet]. 2001 Sep;71(3):334–9. Available from: http://www.pubmedcentral.nih.gov/articlerender.fcgi?artid=1737534&tool=pmcentrez&rendertype=abstract

32. Harbison J, Gibson GJ, Birchall D, Zammit-Maempel I, Ford GA. White matter disease and sleep-disordered breathing after acute stroke. Neurology [Internet]. 2003 Oct 13 [cited 2016 Jan 7];61(7):959–63. Available from: http://www.neurology.org/content/61/7/959.short

33. Macey PM, Kumar R, Woo M a, Valladares EM, Yan-Go FL, Harper RM. Brain structural changes in obstructive sleep apnea. Sleep [Internet]. 2008 Jul;31(7):967–77. Available from: http://www.ncbi.nlm.nih.gov/pubmed/21300501

34. Ramos AR, Dong C, Rundek T, Elkind MS V, Boden-Albala B, Sacco RL, et al. Sleep duration is associated with white matter hyperintensity volume in older adults: the Northern Manhattan Study. J Sleep Res [Internet]. 2014 Jul 7 [cited 2014 Sep 8];i. Available from: http://www.ncbi.nlm.nih.gov/pubmed/25040435

35. Unger MM, Belke M, Menzler K, Heverhagen JT, Keil B, Stiasny-Kolster K, et al. Diffusion tensor imaging in idiopathic REM sleep behavior disorder reveals microstructural changes in the brainstem, substantia nigra, olfactory region, and other brain regions. Sleep [Internet]. American Academy of Sleep Medicine; 2010 Jun 1 [cited 2015 Dec 28];33(6):767–73. Available from: /pmc/articles/PMC2881532/?report=abstract

36. Macey PM, Henderson LA, Macey KE, Alger JR, Frysinger RC, Woo MA, et al. Brain morphology associated with obstructive sleep apnea. Am J Respir Crit Care Med [Internet]. American Thoracic Society; 2002 Nov 15 [cited 2014 Nov 16];166(10):1382–7. Available from: http://www.atsjournals.org/doi/abs/10.1164/rccm.200201-050OC#.VGkRkfmsXIk

For peer review only - http://bmjopen.bmj.com/site/about/guidelines.xhtml

37. Sexton CE, Storsve AB, Walhovd KB, Johansen-Berg H, Fjell AM. Poor sleep quality is associated with increased cortical atrophy in community-dwelling adults. Neurology [Internet]. 2014 Sep 9 [cited 2016 Jun 21];83(11):967–73. Available from: http://www.ncbi.nlm.nih.gov/pubmed/25186857

38. Debette S, Markus HS. The clinical importance of white matter hyperintensities on brain magnetic resonance imaging: systematic review and meta-analysis. BMJ [Internet]. 2010 Jul 26 [cited 2016 Jan 12];341(jul26 1):c3666–c3666. Available from: http://www.bmj.com/content/341/bmj.c3666

39. Mädler B, Drabycz SA, Kolind SH, Whittall KP, MacKay AL. Is diffusion anisotropy an accurate monitor of myelination? Correlation of multicomponent T2 relaxation and diffusion tensor anisotropy in human brain. Magn Reson Imaging [Internet]. 2008 Sep [cited 2016 Jan 6];26(7):874–88. Available from: http://www.ncbi.nlm.nih.gov/pubmed/18524521

40. Jones DK, Knösche TR, Turner R. White matter integrity, fiber count, and other fallacies: The do's and don'ts of diffusion MRI. Vol. 73, NeuroImage. 2013. p. 239–54.

41. Maillard P, Fletcher E, Harvey D, Carmichael O, Reed B, Mungas D, et al. White matter hyperintensity penumbra. Stroke [Internet]. 2011 Jul 1 [cited 2016 Jan 6];42(7):1917–22. Available from: http://stroke.ahajournals.org/content/42/7/1917.short

42. Kievit RA, Davis SW, Griffiths J, Correia MM, Cam-CAN, Henson RN. A watershed model of individual differences in fluid intelligence. Neuropsychologia. 2016;91:186–98.

43. Spiegel K, Tasali E, Leproult R, Van Cauter E. Effects of poor and short sleep on glucose metabolism and obesity risk. Nat Rev Endocrinol [Internet]. Nature Publishing Group; 2009 May [cited 2015 Aug 4];5(5):253–61. Available from: http://dx.doi.org/10.1038/nrendo.2009.23

44. Grandner MA, Jackson NJ, Izci-Balserak B, Gallagher RA, Murray-Bachmann R, Williams NJ, et al. Social and Behavioral Determinants of Perceived Insufficient Sleep. Front Neurol [Internet]. Frontiers; 2015 Jan 5 [cited 2015 Aug 3];6:112. Available from: http://journal.frontiersin.org/article/10.3389/fneur.2015.00112/abstract

45. Leng Y, Wainwright NWJ, Cappuccio FP, Surtees PG, Hayat S, Luben R, et al. Daytime napping and the risk of all-cause and cause-specific mortality: a 13-year follow-up of a British population. Am J Epidemiol [Internet]. 2014 May 1 [cited 2014 Aug 27];179(9):1115–24. Available from: http://www.pubmedcentral.nih.gov/articlerender.fcgi?artid=3992821&tool=pmcentrez&rendertype=abstract

46. Leng Y, Cappuccio FP, Wainwright NWJ, Surtees PG, Luben R, Brayne C, et al. Sleep duration and risk of fatal and nonfatal stroke: a prospective study and meta-analysis. Neurology [Internet]. 2015 Mar 17 [cited 2016 Jan 28];84(11):1072–9. Available from: http://www.neurology.org/content/early/2015/02/25/WNL.0000000000001371.abstract

47. Hoevenaar-Blom MP, Spijkerman AMW, Kromhout D, van den Berg JF, Verschuren WMM. Sleep duration and sleep quality in relation to 12-year cardiovascular disease incidence: the MORGEN study. Sleep [Internet]. 2011 Nov [cited 2016 Jan 6];34(11):1487–92. Available from: http://www.pubmedcentral.nih.gov/articlerender.fcgi?artid=3198203&tool=pmcentrez&rendertype=abstract

48. Strine TW, Chapman DP. Associations of frequent sleep insufficiency with health-related quality of life and health behaviors. Sleep Med [Internet]. 2005 Jan [cited 2015 Oct 19];6(1):23–7. Available from:

For peer review only - http://bmjopen.bmj.com/site/about/guidelines.xhtml

http://www.sciencedirect.com/science/article/pii/S1389945704001078

49. Taheri S, Lin L, Austin D, Young T, Mignot E. Short sleep duration is associated with reduced leptin, elevated ghrelin, and increased body mass index. PLoS Med [Internet]. Public Library of Science; 2004 Dec 7 [cited 2015 Nov 1];1(3):e62. Available from: http://journals.plos.org/plosmedicine/article?id=10.1371/journal.pmed.0010062

50. Roberts RE, Shema SJ, Kaplan GA, Strawbridge WJ. Sleep Complaints and Depression in an Aging Cohort: A Prospective Perspective. Am J Psychiatry [Internet]. American Psychiatric Publishing; 2000 Jan 1 [cited 2015 Jun 16];157(1):81–8. Available from: http://ajp.psychiatryonline.org/doi/10.1176/ajp.157.1.81

51. Breslau N, Roth T, Rosenthal L, Andreski P. Sleep disturbance and psychiatric disorders: A longitudinal epidemiological study of young Adults. Biol Psychiatry [Internet]. 1996 Mar [cited 2015 Apr 8];39(6):411–8. Available from: http://www.sciencedirect.com/science/article/pii/0006322395001883

52. Kaneita Y, Ohida T, Uchiyama M, Takemura S, Kawahara K, Yokoyama E, et al. The Relationship Between Depression and Sleep Disturbances: A Japanese Nationwide General Population Survey. J Clin Psychiatry [Internet]. 2006 Feb [cited 2015 Jun 16];67(2):196–203. Available from: http://www.ncbi.nlm.nih.gov/pubmed/16566613

53. Fried EI, Nesse RM. Depression sum-scores don't add up: why analyzing specific depression symptoms is essential. BMC Med [Internet]. 2015 Apr 6 [cited 2015 Apr 9];13(1):72. Available from: http://www.biomedcentral.com/1741-7015/13/72

54. Novati A, Hulshof HJ, Koolhaas JM, Lucassen PJ, Meerlo P. Chronic sleep restriction causes a decrease in hippocampal volume in adolescent rats, which is not explained by changes in glucocorticoid levels or neurogenesis. Neuroscience [Internet]. 2011 Sep 8 [cited 2015 Jan 20];190:145–55. Available from: http://www.sciencedirect.com/science/article/pii/S0306452211007111

55. Ramsawh HJ, Stein MB, Belik S-L, Jacobi F, Sareen J. Relationship of anxiety disorders, sleep quality, and functional impairment in a community sample. J Psychiatr Res [Internet]. 2009 Jul [cited 2015 Dec 7];43(10):926–33. Available from: http://www.sciencedirect.com/science/article/pii/S0022395609000211

56. Zigmond AS, Snaith RP. The hospital anxiety and depression scale. Acta Psychiatr Scand [Internet]. 1983 Jun [cited 2014 Jul 11];67(6):361–70. Available from: http://www.ncbi.nlm.nih.gov/pubmed/6880820

57. Wechsler CJ. Wechsler Memory Scale. 3d UK. London: Harcourt; 1999.

58. Cattell RB. Abilities: their structure, growth, and action. Boston: Houghton-Mifflin; 1971.

59. Hua K, Zhang J, Wakana S, Jiang H, Li X, Reich DS, et al. Tract probability maps in stereotaxic spaces: analyses of white matter anatomy and tract-specific quantification. Neuroimage. 2008 Jan;39(1):336–47.

60. McGee DL, Liao Y, Cao G, Cooper RS. Self-reported Health Status and Mortality in a Multiethnic US Cohort. Am J Epidemiol [Internet]. 1999 Jan 1 [cited 2015 Nov 25];149(1):41–6. Available from: http://aje.oxfordjournals.org/content/149/1/41.short

61. Deurenberg P, Weststrate JA, Seidell JC. Body mass index as a measure of body fatness: age- and sex-specific prediction formulas. Br J Nutr [Internet]. Cambridge University Press; 2007 Mar 9 [cited 2015 Oct 7];65(2):105. Available from: http://journals.cambridge.org/abstract_S0007114591000193

For peer review only

For peer review only - http://bmjopen.bmj.com/site/about/guidelines.xhtml

62. Liang F, Paulo R, Molina G, Clyde MA, Berger JO. Mixtures of g Priors for Bayesian Variable Selection. J Am Stat Assoc [Internet]. Taylor & Francis; 2008 Mar 1 [cited 2015 Oct 7];103(481):410–23. Available from: http://amstat.tandfonline.com/doi/abs/10.1198/016214507000001337#.VhTxTPl3lpg

63. Rouder JN, Morey RD. Default Bayes Factors for Model Selection in Regression. Multivariate Behav Res [Internet]. Taylor & Francis Group; 2012 Nov 17 [cited 2015 Jun 16];47(6):877–903. Available from: http://www.tandfonline.com/doi/abs/10.1080/00273171.2012.734737

64. Wagenmakers E-J. A practical solution to the pervasive problems ofp values. Psychon Bull Rev [Internet]. 2007 Oct [cited 2015 Jun 16];14(5):779–804. Available from: http://www.springerlink.com/index/10.3758/BF03194105

65. Wetzels R, Matzke D, Lee MD, Rouder JN, Iverson GJ, Wagenmakers E-J. Statistical Evidence in Experimental Psychology: An Empirical Comparison Using 855 t Tests. Perspect Psychol Sci [Internet]. 2011 May 18 [cited 2015 May 12];6(3):291–8. Available from: http://pps.sagepub.com/content/6/3/291.short

66. Gelman A, Hill J, Yajima M. Why We (Usually) Don't Have to Worry About Multiple Comparisons. J Res Educ Eff [Internet]. Taylor & Francis Group; 2012 Apr 3 [cited 2014 Jul 15];5(2):189–211. Available from: http://www.tandfonline.com/doi/abs/10.1080/19345747.2011.618213

67. Jeffreys H. A theory of probability. Oxford: Oxford University Press; 1961.

68. Morey RD, Rouder JN. BayesFactor. CRAN; 2015.

69. Team. R: a language and environment for statistical computing. Vienna; 2013.

70. Jeffreys H. Theory of Probability. Oxford: Oxford University Press; 1961.

71. Linzer DA, Lewis JB. poLCA: An R Package for Polytomous Variable Latent Class Analysis [Internet]. Journal of Statistical Software. 2011 [cited 2014 Sep 8]. p. 42: 10. Available from: http://www.jstatsoft.org/v42/i10/paper

72. Rhemtulla M, Brosseau-Liard PÉ, Savalei V. When can categorical variables be treated as continuous? A comparison of robust continuous and categorical SEM estimation methods under suboptimal conditions. Psychol Methods [Internet]. American Psychological Association; 2012 [cited 2017 Feb 10];17(3):354–73. Available from: http://doi.apa.org/getdoi.cfm?doi=10.1037/a0029315

73. Schwarz G. Estimating the Dimension of a Model. Ann Stat [Internet]. Institute of Mathematical Statistics; 1978 Mar 1 [cited 2015 Jun 16];6(2):461–4. Available from: http://projecteuclid.org/euclid.aos/1176344136

74. Dayton CM, Macready GB. Concomitant-Variable Latent-Class Models. J Am Stat Assoc [Internet]. Taylor & Francis; 1988 Mar [cited 2014 Sep 8];83(401):173–8. Available from: http://www.tandfonline.com/doi/abs/10.1080/01621459.1988.10478584

75. Wickham H. ggplot2: Elegant Graphics for Data Analysis [Internet]. Springer Science & Business Media; 2009 [cited 2015 Aug 4]. 221 p. Available from: https://books.google.com/books?hl=en&lr=&id=bes-AAAAQBAJ&pgis=1

76. Miller E. Verbal fluency as a function of a measure of verbal intelligence and in relation to different types of cerebral pathology. Br J Clin Psychol [Internet]. 1984 Feb 12 [cited 2016 Jan 7];23(1):53–7. Available from: http://doi.wiley.com/10.1111/j.2044-8260.1984.tb00626.x

77. Biesbroek JM, van Zandvoort MJE, Kappelle LJ, Velthuis BK, Biessels GJ, Postma A. Shared and distinct anatomical correlates of semantic and phonemic fluency revealed by lesion-symptom

For peer review only

mapping in patients with ischemic stroke. Brain Struct Funct [Internet]. Springer Berlin Heidelberg; 2016 May 5 [cited 2017 Jan 31];221(4):2123–34. Available from: http://link.springer.com/10.1007/s00429-015-1033-8

78. Troyer AK, Moscovitch M, Winocur G. Clustering and switching as two components of verbal fluency: Evidence from younger and older healthy adults. Neuropsychology [Internet]. 1997 [cited 2016 Jan 7];11(1). Available from: http://psycnet.apa.orgjournals/neu/11/1/138

79. Kievit RA, Davis SW, Griffiths JD, Correia MM, Henson RNA. A watershed model of individual differences in fluid intelligence. bioRxiv [Internet]. Cold Spring Harbor Labs Journals; 2016 Feb 26 [cited 2016 Mar 29];41368. Available from: http://www.biorxiv.org/content/early/2016/02/26/041368.abstract

80. Briones B, Adams N, Strauss M, Rosenberg C, Whalen C, Carskadon M, et al. Relationship between sleepiness and general health status. Sleep [Internet]. 1996 Sep [cited 2015 Aug 4];19(7):583–8. Available from: http://www.ncbi.nlm.nih.gov/pubmed/8899938

81. Cizza G, Skarulis M, Mignot E. A link between short sleep and obesity : Building the evidence for causation. Sleep [Internet]. American Academy of Sleep Medicine; 2005 [cited 2016 Jan 12];28(10):1217–20. Available from: http://cat.inist.fr/?aModele=afficheN&cpsidt=17179376

82. Gangwisch JE, Malaspina D, Boden-Albala B, Heymsfield SB. Inadequate sleep as a risk factor for obesity: analyses of the NHANES I. Sleep [Internet]. 2005 Oct [cited 2015 Sep 3];28(10):1289–96. Available from: http://europepmc.org/abstract/med/16295214

83. Grandner MA, Drummond SPA. Who are the long sleepers? Towards an understanding of the mortality relationship. Sleep Med Rev. 2007;11(5):341–60.

84. KANEITA Y, UCHIYAMA M, YOSHIIKE N, OHIDA T. Associations of Usual Sleep Duration with Serum Lipid and Lipoprotein Levels. Sleep. American Academy of Sleep Medicine; 31(5):645–52.

85. Grandner MA, Hale L, Moore M, Patel NP. Mortality associated with short sleep duration: The evidence, the possible mechanisms, and the future. Sleep Med Rev. 2010;14(3):191–203.

86. Kievit RA, Davis SW, Mitchell D, Taylor JR, Duncan J, Cam-CAN, et al. Distinct aspects of frontal lobe structure mediate age-related differences in fluid intelligence and multitasking. Nat Commun. 2014;

87. Henson RN, Campbell KL, Davis SW, Taylor JR, Emery T, Erzinclioglu S, et al. Multiple determinants of lifespan memory differences. Sci Rep [Internet]. Nature Publishing Group; 2016 Sep 7 [cited 2017 Feb 10];6:32527. Available from: http://www.nature.com/articles/srep32527

88. Fox RJ, Sakaie K, Lee J-C, Debbins JP, Liu Y, Arnold DL, et al. A validation study of multicenter diffusion tensor imaging: reliability of fractional anisotropy and diffusivity values. AJNR Am J Neuroradiol [Internet]. American Society of Neuroradiology; 2012 Apr [cited 2017 Feb 10];33(4):695–700. Available from: http://www.ncbi.nlm.nih.gov/pubmed/22173748

89. Wandell BA. Clarifying Human White Matter. Annu Rev Neurosci [Internet]. Annual Reviews ; 2016 Jul 8 [cited 2017 Feb 10];39(1):103–28. Available from: http://www.annualreviews.org/doi/10.1146/annurev-neuro-070815-013815

90. Tournier J-D, Mori S, Leemans A. Diffusion tensor imaging and beyond. Magn Reson Med [Internet]. Wiley Subscription Services, Inc., A Wiley Company; 2011 Jun [cited 2017 Feb 10];65(6):1532–56. Available from: http://doi.wiley.com/10.1002/mrm.22924

91. Lustberg L, Reynolds CF. Depression and insomnia: questions of cause and effect. Sleep Med

For peer review only

For peer review only - http://bmjopen.bmj.com/site/about/guidelines.xhtml

Rev [Internet]. 2000 Jun [cited 2015 Dec 28];4(3):253–62. Available from: http://www.sciencedirect.com/science/article/pii/S1087079299900758

92. Sbarra DA, Allen JJB. Decomposing depression: On the prospective and reciprocal dynamics of mood and sleep disturbances.

93. Lim ASP, Ellison BA, Wang JL, Yu L, Schneider JA, Buchman AS, et al. Sleep is related to neuron numbers in the ventrolateral preoptic/intermediate nucleus in older adults with and without Alzheimer's disease. Brain [Internet]. 2014 Oct 20 [cited 2015 Dec 16];137(Pt 10):2847–61. Available from: http://brain.oxfordjournals.org/content/early/2014/08/11/brain.awu222

94. Raz N, Lindenberger U. Only time will tell: Cross-sectional studies offer no solution to the age–brain–cognition triangle: Comment on Salthouse (2011).

95. Schaie KW. The course of adult intellectual development.

96. Wiberg M, Sundstrom A. A Comparison of Two Approaches to Correction of Restriction of Range in Correlation Analysis. Pract Assessment, Res Eval [Internet]. Dr. Lawrence M. Rudner. e-mail: editor@pareonline.net; Web site: http://pareonline.net; 2009 Feb 28 [cited 2016 Feb 19];14(5). Available from: http://eric.ed.gov/?id=EJ933658

For peer review only

For peer review only - http://bmjopen.bmj.com/site/about/guidelines.xhtml

Legends

Figure 1. Descriptive interpretation of Bayes Factors

Figure 2. Latent Class Analysis. Panel A shows the sleep quality profiles for each of the four classes. Panel B shows the conditional probability of belonging to each class across the lifespan.

Figure 3. Simple regressions between sleep components and Cognitive Health.
The strength of the effect is colour-coded by Bayes Factor, and the effect size is shown as r-squared (as a percentage out of 100). Sample varies across components and measures due to varying missingness. Cattell and Reaction Time were measured only in the imaging cohort: mean N = 648, N=11.11. Sample sizes for 5 other domains are similar: mean N= 2300.25, SD= 65.57)

Figure 4. Multiple regressions between sleep components and Neural Health. Each cell represents the relationship between a sleep component and the mean neural health in a given tract as index by Fractional Anisotropy. Numbers represent R-squared, the sample size is show in the last column. Strong associations are observed between measures of Sleep Efficiency and multiple tracts, along with sporadic associations between other components and tracts. White matter tracts abbreviations: Uncinate fasciculus (UNC), superior longitudinal fasciculus (SLF), inferior longitudinal fasciculus (ILF), inferior Fronto-occipital fasciculus (IFOF), forceps minor (FMin), forceps major (FMaj), cerebrospinal tract (CST), the ventral cingulate gyrus (CINGHipp), the dorsal cingulate gyrus (CING), and the anterior thalamic radiations (ATR). N varies slightly across components due to varying missingness (N mean = 631.325, SD = 10.32).

Figure 5 Physical health and sleep quality. Numbers represent Rsquared, the sample size is show in the last column. Strong associations between general indices of health and sleep quality are found, and several more modest relationships with BMI and sleep quality. Self-reported health (12 month and General) were measured in the full cohort (Mean = 2315.37, SD=66.29), the other indicators were measured in the imaging cohort only (Mean = 569.87, SD= 11.16).

Figure 6. Interaction between sleep quality and anxiety. (N=724, age 18.48 to 46.2) compared to the oldest third of participants (N=725, age 71.79 to 98.88).

Figure 7. Curvilinear associations between sleep duration in hours and A) HADS depression and B) general health (self-reported). For visual clarity a small amount of random jitter was added to the data points.

For peer review only - http://bmjopen.bmj.com/site/about/guidelines.xhtml

Bayes Factor BF_{10}	Log BF_{10}	Tileplot colour	Description (Jeffreys, 1961)
>100	>4.6		Extreme evidence for H1
30 – 100	3.4 – 4-6		Very strong evidence for H1
10 – 30	2.3 – 3.4		Strong evidence for H1
3 – 10	1.098 – 2.3		Substantial evidence for H1
1 – 3	1 – 1.098		Anecdotal evidence for H1
1	0		No evidence either way
1/3 – 1	-1.098 – -1		Anecdotal evidence for H0
1/3 - 1/10	-2.3 – -1.098		Substantial evidence for H0
1/10 - 1/30	-3.4 – -2.3		Strong evidence for H0
1/30 - 1/100	-4.6 – - 3.4		Very strong evidence for H0
<1/100	< -4.6		Extreme evidence for H0

Figure 1. Descriptive interpretation of Bayes Factors

Insert Figure 1 here
338x190mm (96 x 96 DPI)

For peer review only

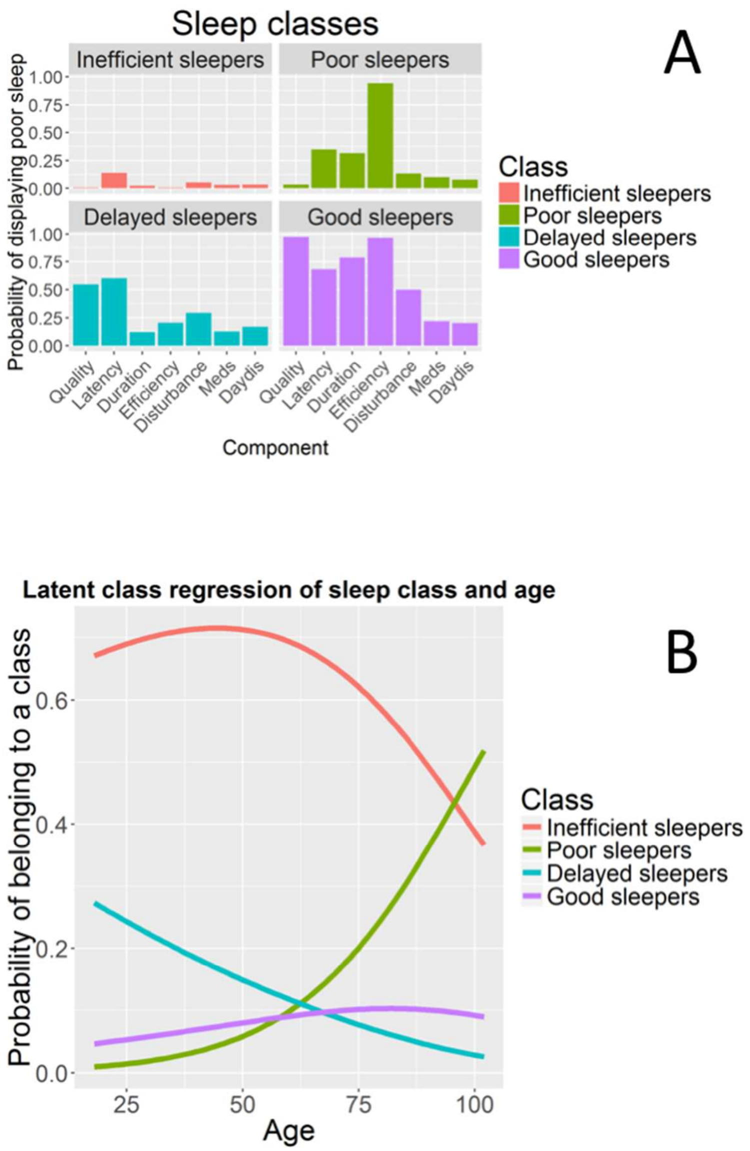

Figure 2. Latent Class Analysis. Panel A shows the sleep quality profiles for each of the four classes. Panel B shows the conditional probability of belonging to each class across the lifespan.

Insert Figure 2 here
60x89mm (300 x 300 DPI)

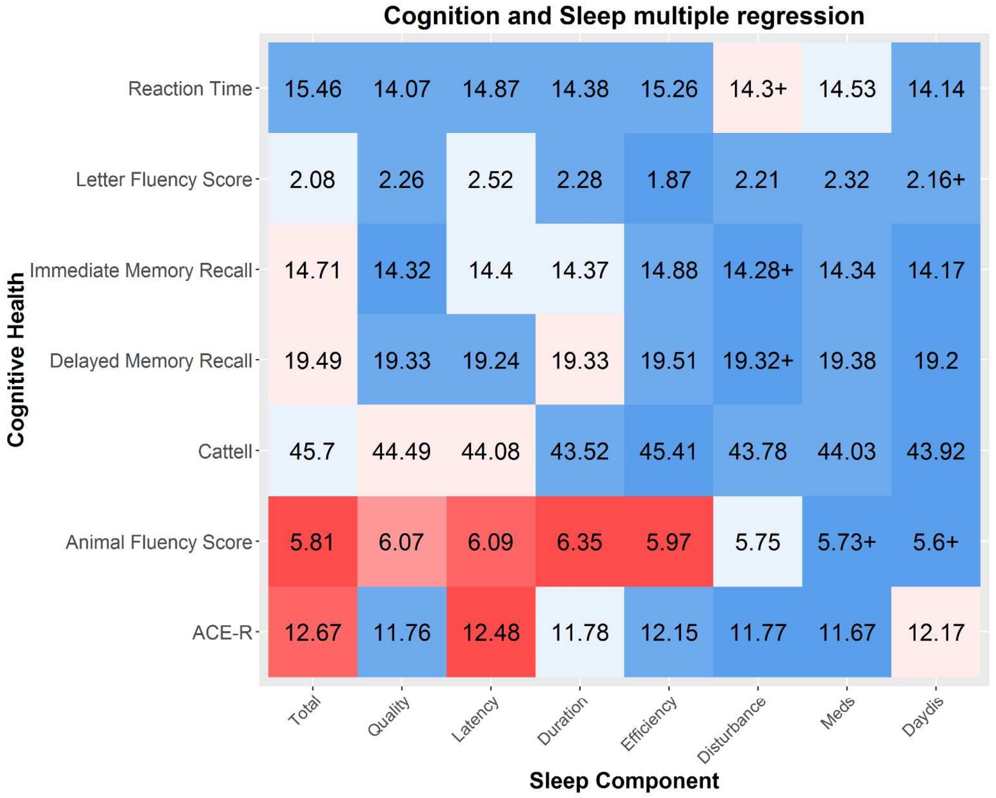

Figure 3. Simple regressions between sleep components and Cognitive Health.
The strength of the effect is colour-coded by Bayes Factor, and the effect size is shown as r-squared (as a percentage out of 100). Sample varies across components and measures due to varying missingness. Cattell and Reaction Time were measured only in the imaging cohort: mean N = 648, N=11.11. Sample sizes for 5 other domains are similar: mean N= 2300.25, SD= 65.57)

Insert Figure 3 here
254x203mm (300 x 300 DPI)

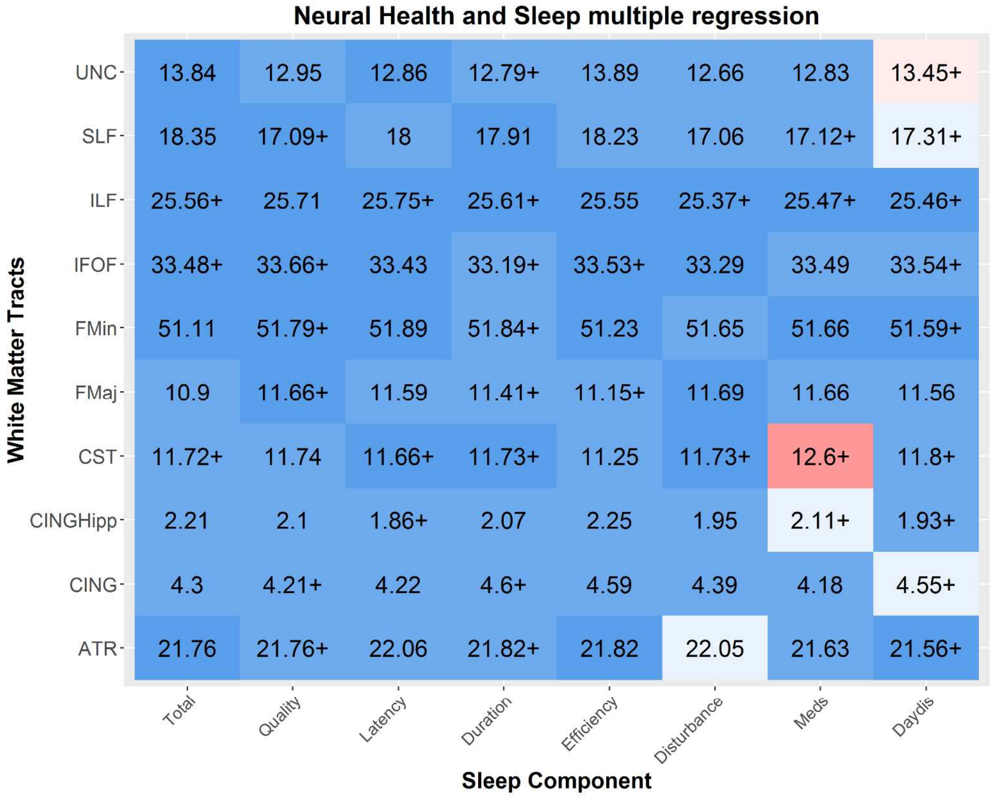

Figure 4. Multiple regressions between sleep components and Neural Health. Each cell represents the relationship between a sleep component and the mean neural health in a given tract as index by Fractional Anisotropy. Numbers represent R-squared, the sample size is show in the last column. Strong associations are observed between measures of Sleep Efficiency and multiple tracts, along with sporadic associations between other components and tracts. White matter tracts abbreviations: Uncinate fasciculus (UNC), superior longitudinal fasciculus (SLF), inferior longitudinal fasciculus (ILF), inferior Fronto-occipital fasciculus (IFOF), forceps minor (FMin), forceps major (FMaj), cerebrospinal tract (CST), the ventral cingulate gyrus (CINGHipp), the dorsal cingulate gyrus (CING), and the anterior thalamic radiations (ATR). N varies slightly across components due to varying missingness (N mean = 631.325, SD = 10.32).

Insert Figure 4 here
254x203mm (300 x 300 DPI)

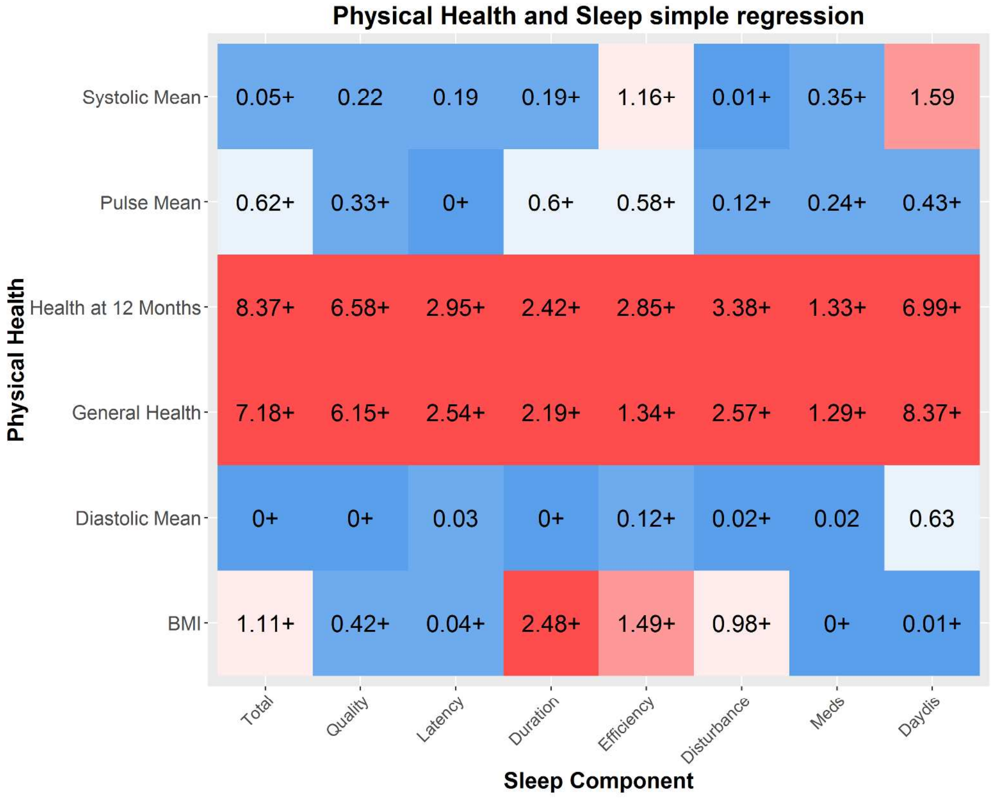

Figure 5 Physical health and sleep quality. Numbers represent Rsquared, the sample size is show in the last column. Strong associations between general indices of health and sleep quality are found, and several more modest relationships with BMI and sleep quality. Self-reported health (12 month and General) were measured in the full cohort (Mean = 2315.37, SD=66.29), the other indicators were measured in the imaging cohort only (Mean = 569.87, SD= 11.16).

Insert Figure 5 here
254x203mm (300 x 300 DPI)

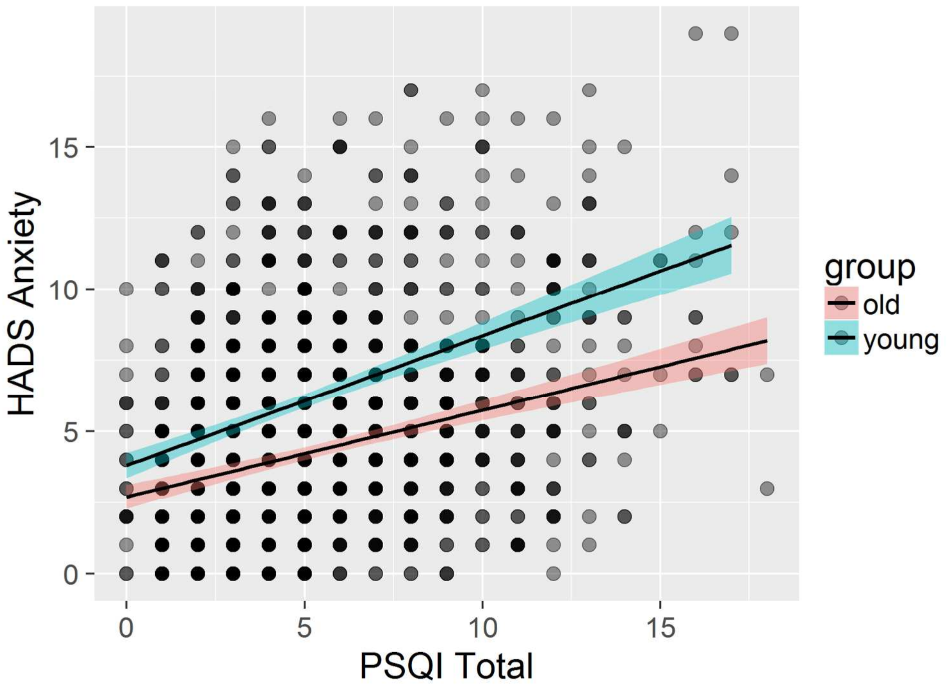

Figure 6. Interaction between sleep quality and anxiety. (N=724, age 18.48 to 46.2) compared to the oldest third of participants (N=725, age 71.79 to 98.88).

Insert Figure 6 here
177x127mm (300 x 300 DPI)

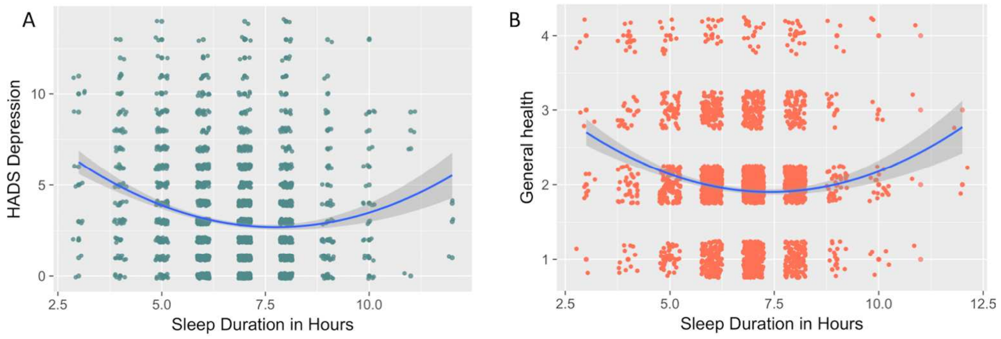

Figure 7. Curvilinear associations between sleep duration in hours and A) HADS depression and B) general health (self-reported). For visual clarity a small amount of random jitter was added to the data points.

Insert Figure 7 here
527x179mm (300 x 300 DPI)

For peer review only

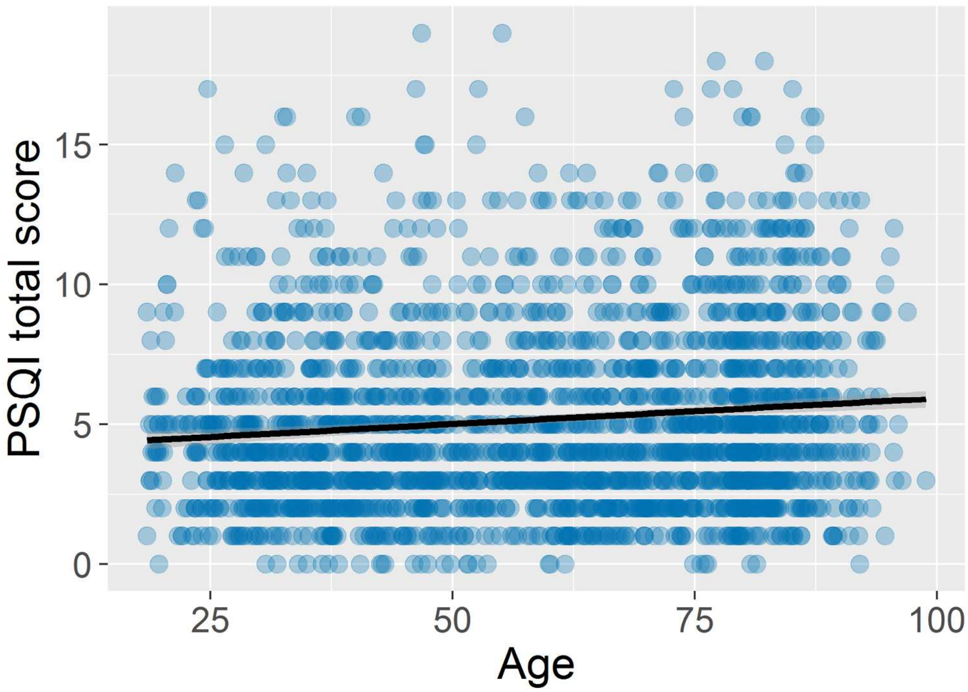

177x127mm (300 x 300 DPI)

iew only

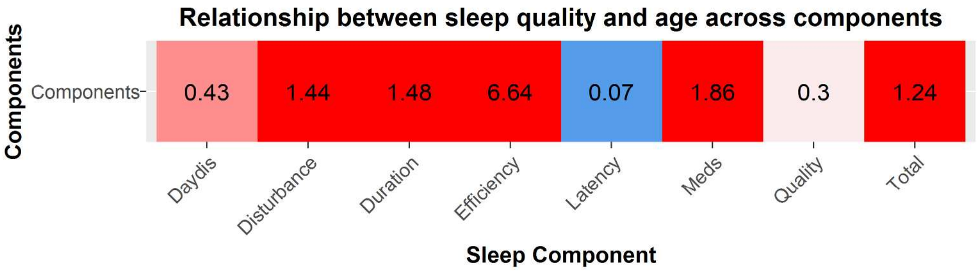

254x127mm (300 x 300 DPI)

For peer review only

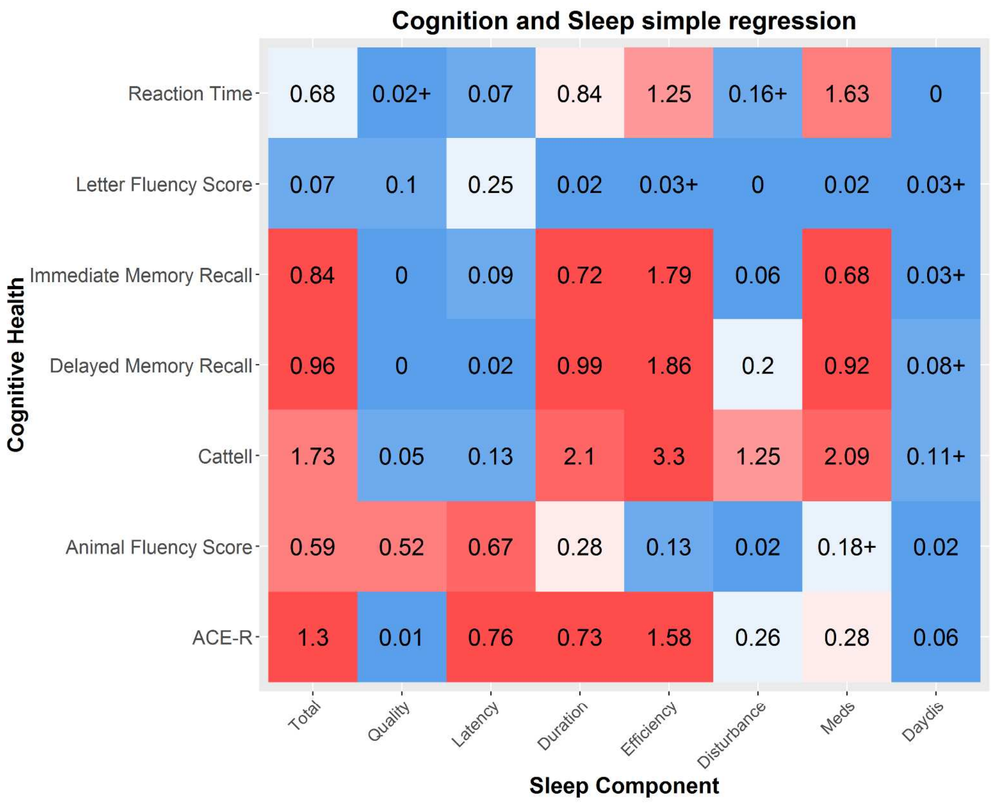

254x203mm (300 x 300 DPI)

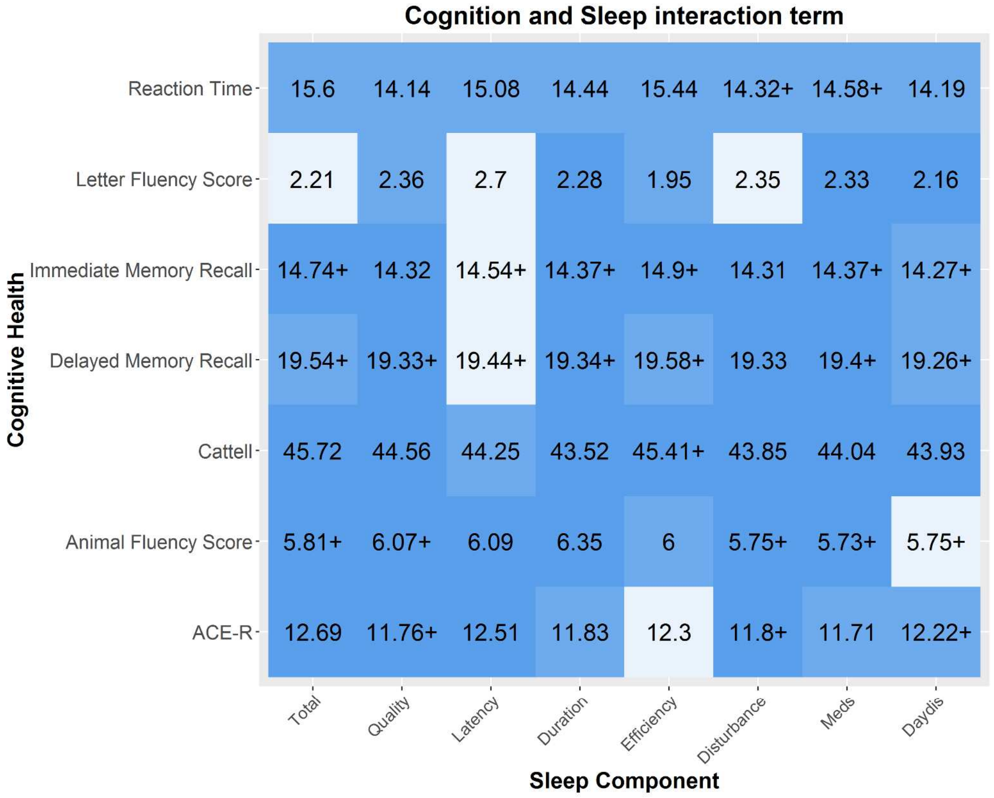

254x203mm (300 x 300 DPI)

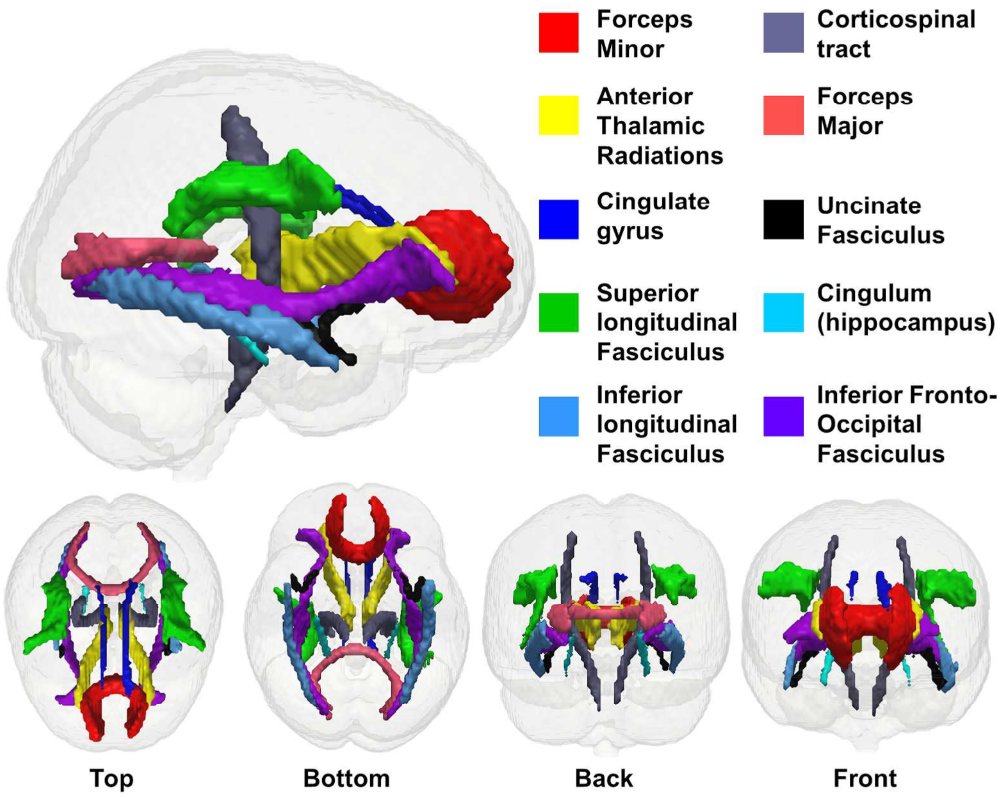

500x400mm (300 x 300 DPI)

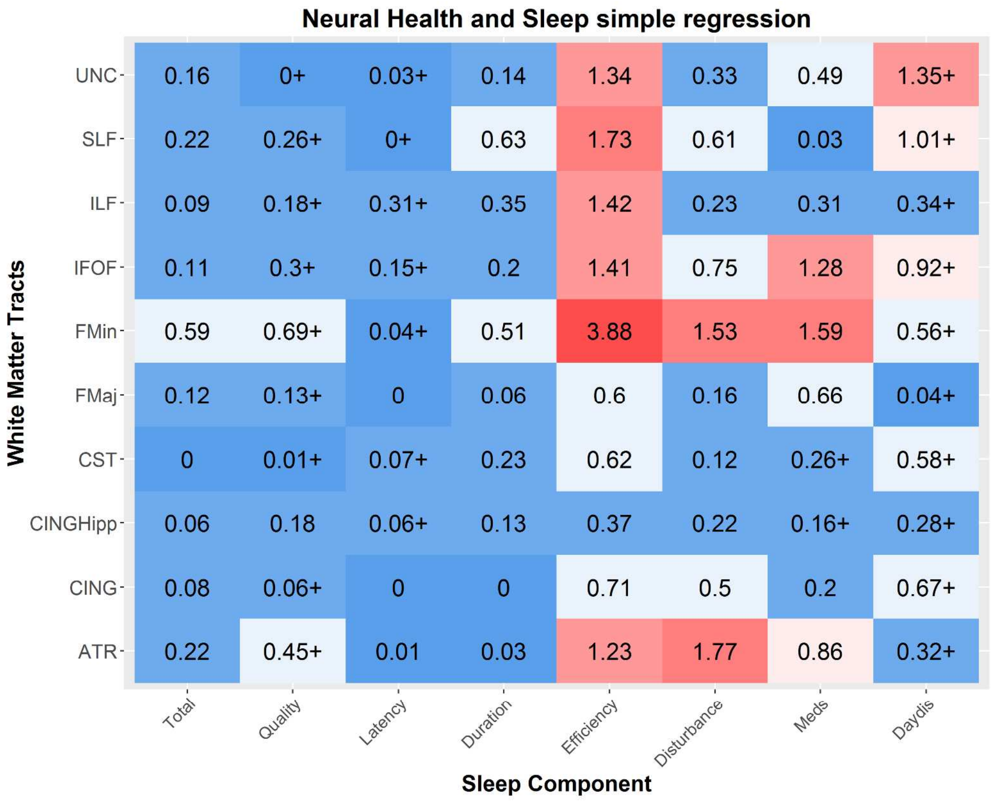

254x203mm (300 x 300 DPI)

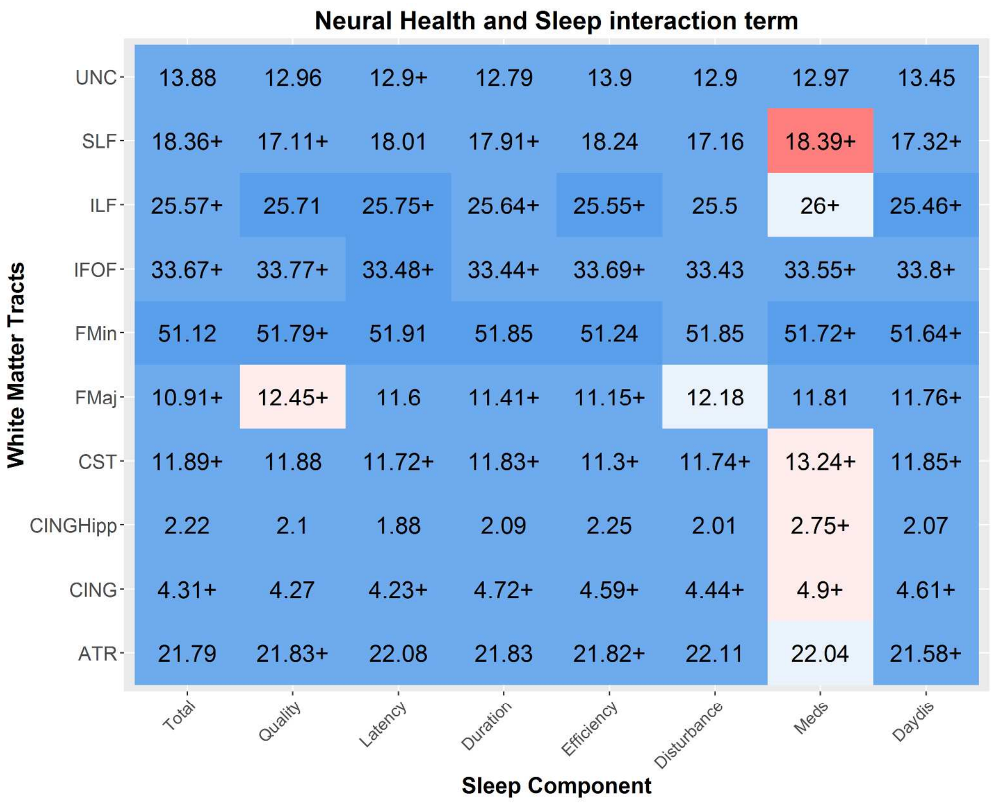

254x203mm (300 x 300 DPI)

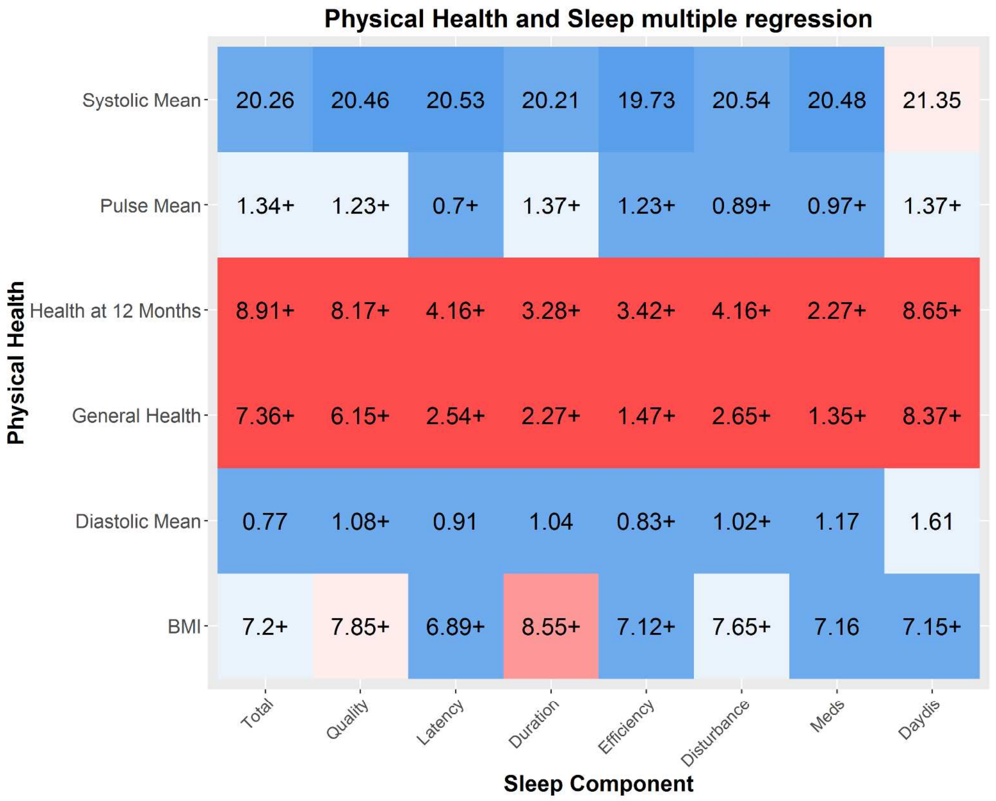

254x203mm (300 x 300 DPI)

w only

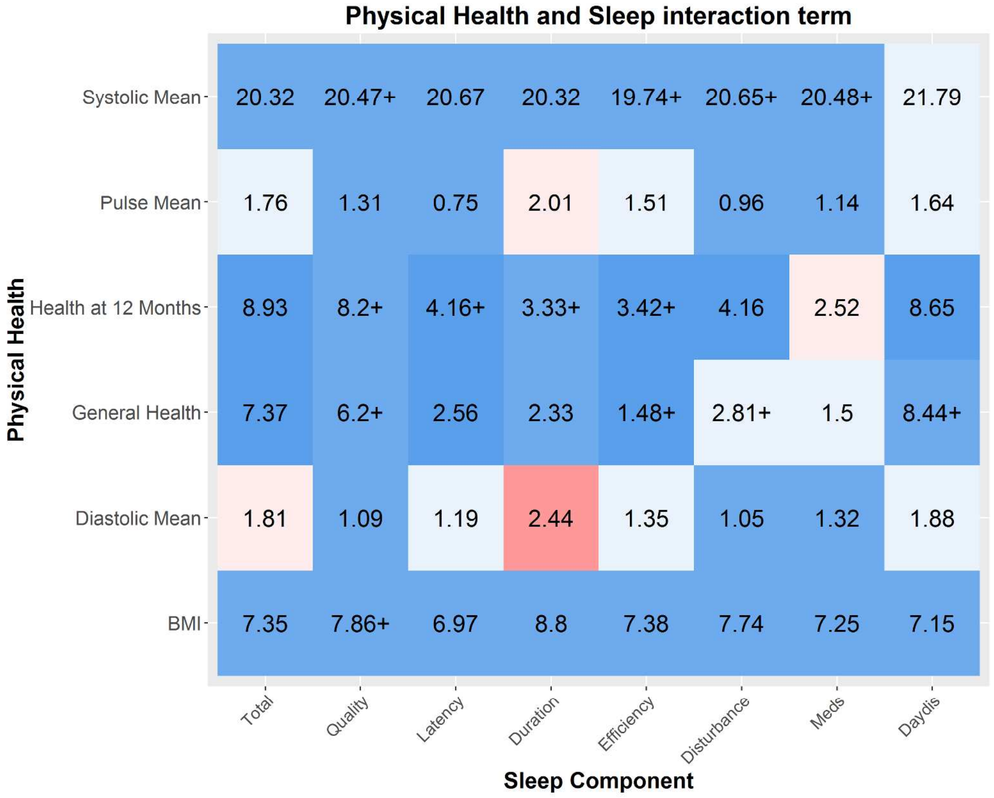

254x203mm (300 x 300 DPI)

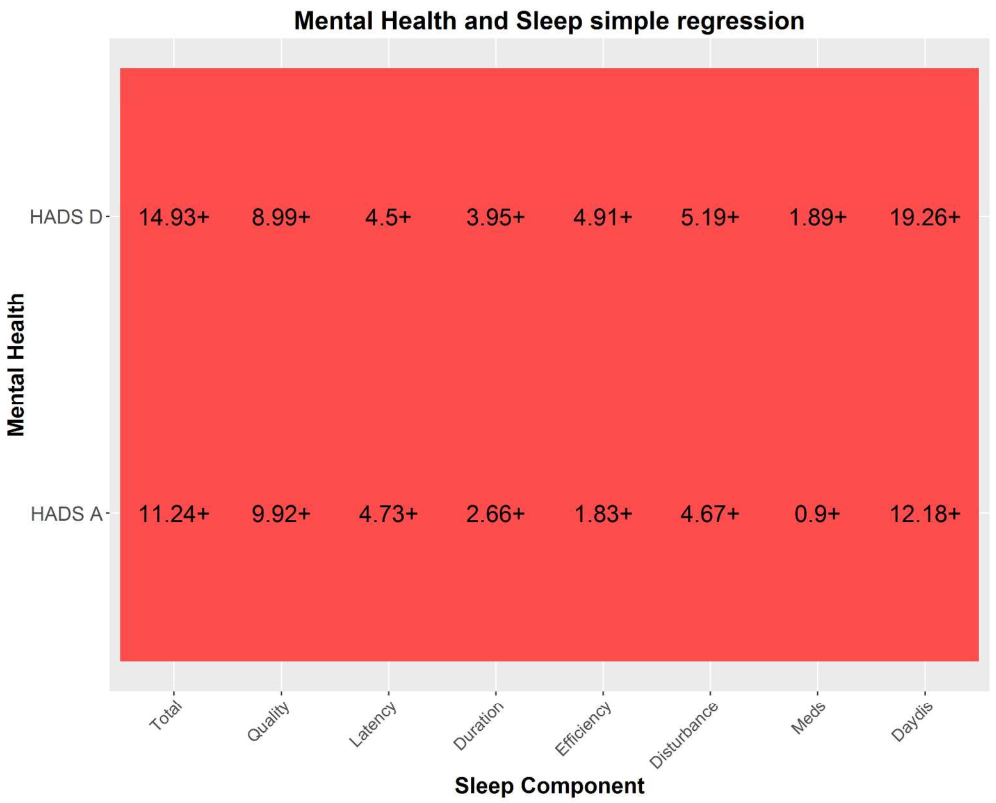

254x203mm (300 x 300 DPI)

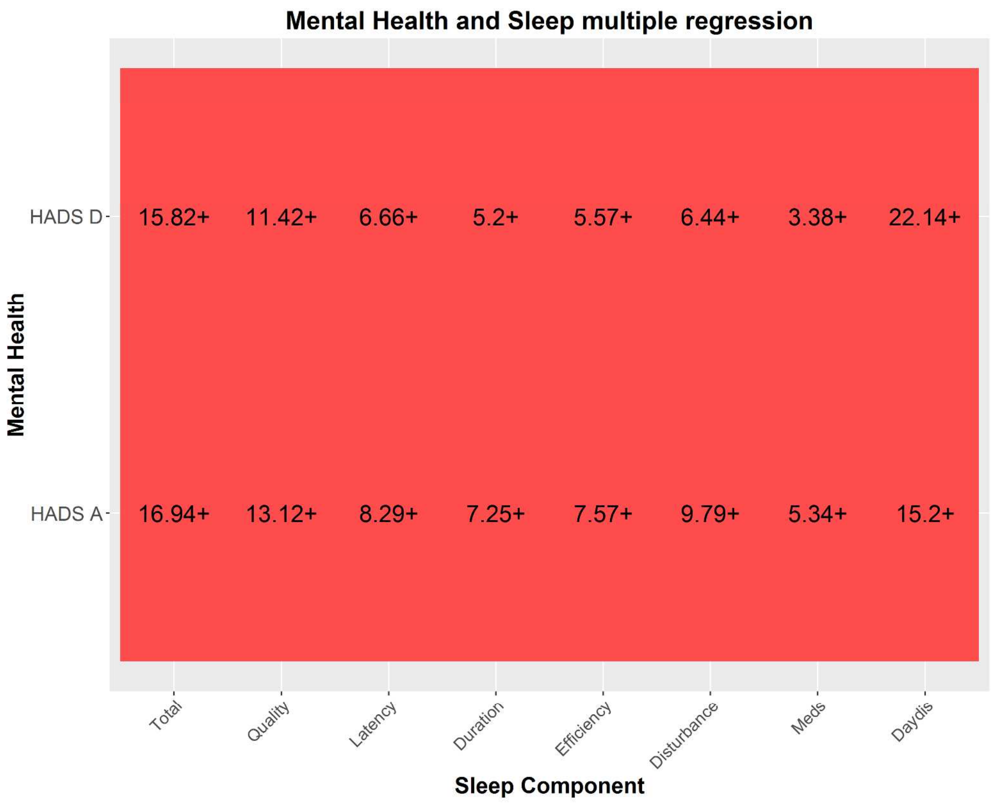

254x203mm (300 x 300 DPI)

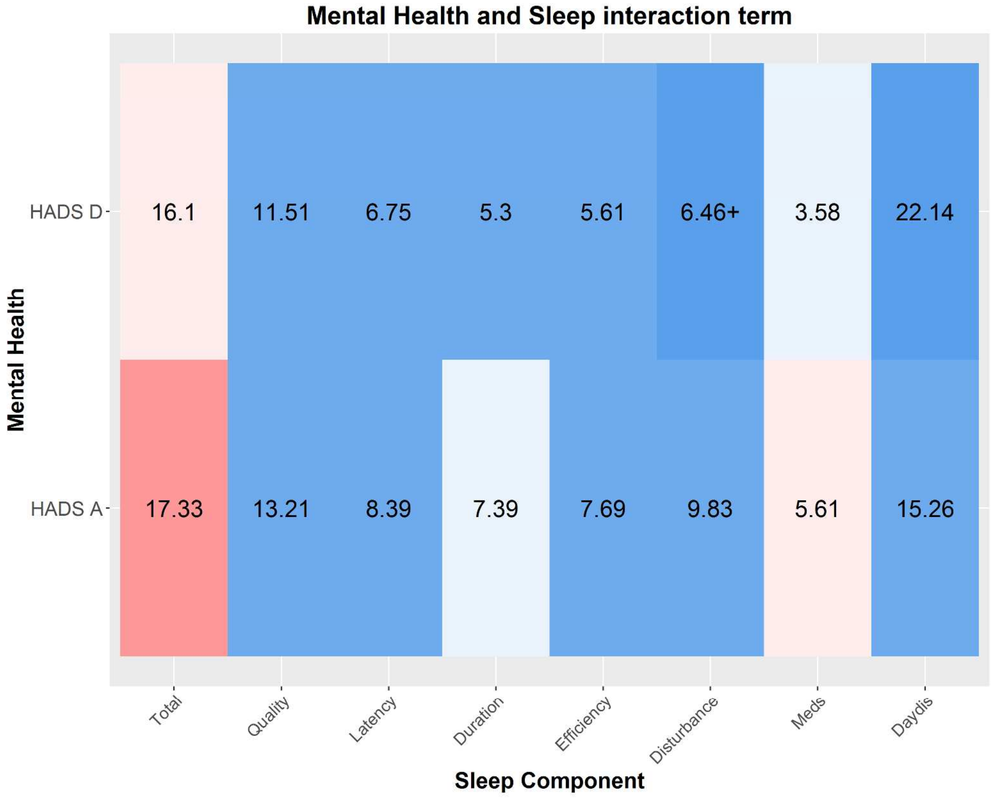

254x203mm (300 x 300 DPI)

STROBE 2007 (v4) Statement—Checklist of items that should be included in reports of *cohort studies*

Section/Topic	Item #	Recommendation	Reported on page #
**Title and abstract**	1	(*a*) Indicate the study's design with a commonly used term in the title or the abstract	1
		(*b*) Provide in the abstract an informative and balanced summary of what was done and what was found	2
**Introduction**			
Background/rationale	2	Explain the scientific background and rationale for the investigation being reported	4
Objectives	3	State specific objectives, including any prespecified hypotheses	5
**Methods**			
Study design	4	Present key elements of study design early in the paper	5
Setting	5	Describe the setting, locations, and relevant dates, including periods of recruitment, exposure, follow-up, and data collection	6
Participants	6	(*a*) Give the eligibility criteria, and the sources and methods of selection of participants. Describe methods of follow-up	6
		(*b*) For matched studies, give matching criteria and number of exposed and unexposed	NA
Variables	7	Clearly define all outcomes, exposures, predictors, potential confounders, and effect modifiers. Give diagnostic criteria, if applicable	5-9
Data sources/ measurement	8*	For each variable of interest, give sources of data and details of methods of assessment (measurement). Describe comparability of assessment methods if there is more than one group	6-11
Bias	9	Describe any efforts to address potential sources of bias	N/A
Study size	10	Explain how the study size was arrived at	6
Quantitative variables	11	Explain how quantitative variables were handled in the analyses. If applicable, describe which groupings were chosen and why	10
Statistical methods	12	(*a*) Describe all statistical methods, including those used to control for confounding	10-13
		(*b*) Describe any methods used to examine subgroups and interactions	10
		(*c*) Explain how missing data were addressed	11
		(*d*) If applicable, explain how loss to follow-up was addressed	N/A
		(*e*) Describe any sensitivity analyses	N/A

For peer review only

**Results**			
Participants	13*	(a) Report numbers of individuals at each stage of study—eg numbers potentially eligible, examined for eligibility, confirmed eligible, included in the study, completing follow-up, and analysed	14-22
		(b) Give reasons for non-participation at each stage	6
		(c) Consider use of a flow diagram	NA
Descriptive data	14*	(a) Give characteristics of study participants (eg demographic, clinical, social) and information on exposures and potential confounders	6
		(b) Indicate number of participants with missing data for each variable of interest	9,10
		(c) Summarise follow-up time (eg, average and total amount)	6
Outcome data	15*	Report numbers of outcome events or summary measures over time	NA
Main results	16	(*a*) Give unadjusted estimates and, if applicable, confounder-adjusted estimates and their precision (eg, 95% confidence interval). Make clear which confounders were adjusted for and why they were included	14-22
		(*b*) Report category boundaries when continuous variables were categorized	NA
		(*c*) If relevant, consider translating estimates of relative risk into absolute risk for a meaningful time period	NA
Other analyses	17	Report other analyses done—eg analyses of subgroups and interactions, and sensitivity analyses	14-22
**Discussion**			
Key results	18	Summarise key results with reference to study objectives	22-26
**Limitations**			
Interpretation	20	Give a cautious overall interpretation of results considering objectives, limitations, multiplicity of analyses, results from similar studies, and other relevant evidence	25-26
Generalisability	21	Discuss the generalisability (external validity) of the study results	25-26
**Other information**			
Funding	22	Give the source of funding and the role of the funders for the present study and, if applicable, for the original study on which the present article is based	3

*Give information separately for cases and controls in case-control studies and, if applicable, for exposed and unexposed groups in cohort and cross-sectional studies.

Note: An Explanation and Elaboration article discusses each checklist item and gives methodological background and published examples of transparent reporting. The STROBE checklist is best used in conjunction with this article (freely available on the Web sites of PLoS Medicine at http://www.plosmedicine.org/, Annals of Internal Medicine at http://www.annals.org/, and Epidemiology at http://www.epidem.com/). Information on the STROBE Initiative is available at www.strobe-statement.org.

For peer review only

BMJ Open

How are age-related differences in sleep quality associated with health outcomes? An epidemiological investigation in a UK cohort of 2406 adults

Journal:	*BMJ Open*
Manuscript ID	bmjopen-2016-014920.R2
Article Type:	Research
Date Submitted by the Author:	07-Mar-2017
Complete List of Authors:	Gadie, Andrew; MRC Cognition and Brain Sciences Unit<br>Shafto, Meredith; University of Cambridge, Center for Speech, Language and the Brain<br>Leng, Yue; University of Cambridge; University of California San Francisco, School of Medicine<br>Cam-CAN, _; University of Cambridge, Center for Sleep, language and the brain<br>Kievit, Rogier; MRC CBSU
**Primary Subject Heading**:	Epidemiology
Secondary Subject Heading:	Neurology, Mental health, Public health, Geriatric medicine
Keywords:	Ageing, SLEEP MEDICINE, cognition, MENTAL HEALTH, Neurobiology < BASIC SCIENCES

SCHOLARONE™
Manuscripts

For peer review only

How are age-related difference in sleep quality associated with health outcomes? An epidemiological investigation in a UK cohort of 2406 adults

Andrew Gadie[1]

Meredith Shafto[2]

Yue Leng[3]

Cam-CAN[4]

Rogier A. Kievit[1]*

For peer review only

*Corresponding author: rogier.kievit@mrc-cbu.cam.ac.uk

1 MRC Cognition and Brain Sciences Unit, 15 Chaucer Rd, Cambridge, CB2 7EF, United Kingdom

2 Department of Psychology, University of Cambridge, Downing Street, Cambridge, CB2 3EB, United Kingdom

3 University of California, San Francisco

4 Cambridge Centre for Ageing and Neuroscience (Cam-CAN), University of Cambridge and MRC Cognition and Brain Sciences Unit, Cambridge, UK, www.cam-can.com

For peer review only - http://bmjopen.bmj.com/site/about/guidelines.xhtml

Abstract

Objectives To examine age related differences in self-reported sleep quality and their associations with health outcomes across four domains: Physical Health, Cognitive Health, Mental Health and Neural Health.

Setting Cam-CAN is a cohort study in East Anglia/England, which collected self-reported health and lifestyle questions as well as a range of objective measures from healthy adults.

Participants 2406 healthy adults (age 18-98) answered questions about their sleep quality (Pittsburgh Sleep Quality Index) and measures of Physical, Cognitive, Mental, and Neural Health. A subset of 641 individuals provided measures of brain structure.

Main outcome measures Pittsburgh Sleep Quality Index scores (PSQI) of sleep, and scores across tests within the four domains of health. Latent Class Analysis (LCA) is used to identify sleep types across the lifespan. Bayesian regressions quantify the presence, and absence, of relationships between sleep quality and health measures.

Results Better sleep is generally associated with better health outcomes, strongly so for mental health, moderately for cognitive and physical health, but not for sleep quality and neural health. Latent Class Analysis identified four sleep types: 'Good sleepers' (68.6%, most frequent in middle age), 'inefficient sleepers' (13.05%, most frequent in old age), 'Delayed sleepers' (9.76%, most frequent in young adults) and 'poor sleepers' (8.6%, most frequent in old age). There is little evidence for interactions between sleep quality and age on health outcomes. Finally, we observe u-shaped associations between sleep duration and mental health (depression and anxiety) as well as self-reported general health, such that both short and long sleep were associated with poorer outcomes.

Conclusions Lifespan changes in sleep quality are multifaceted and not captured well by summary measures, but instead as partially independent symptoms that vary in prevalence across the lifespan. Better self-reported sleep is associated with better health outcomes, and the strength

For peer review only - http://bmjopen.bmj.com/site/about/guidelines.xhtml

of these associations differs across health domains. Notably, we do observed associations between self-reported sleep quality and white matter.

Funding Biotechnology and Biological Sciences Research Council (grant number BB/H008217/1). RAK is supported by the Wellcome Trust (grant number 107392/Z/15/Z and the UK Medical Research Council (MC-A060-5PR61).

Keywords

Ageing, sleep quality, healthy ageing, cognition, mental health, cognition, white matter, physical health

Strengths and limitations of this study

- Broad phenotypic assessment of healthy ageing across multiple health domains
- Advanced analytic techniques (i.e. Latent Class Analysis regression) allows new insights
- A uniquely large neuroimaging sample combined with Bayesian inference allows for quantification of evidence for the null hypothesis
- Subjective sleep measures may have drawbacks in older samples
- Cross-sectional data precludes modelling of within subject changes

For peer review only

BACKGROUND

Sleep is a fundamental human behaviour, with humans spending almost a third of their lives asleep. Regular and sufficient sleep has been shown to benefit human physiology through a number of different routes, ranging from consolidation of memories (1) to removal of free radicals (2) and neurotoxic waste (3). Sleep patterns are known to change across the lifespan in various ways. including decreases in quantity and quality of sleep (4), with up to 50% of older adults report difficulties initiating and/or maintaining sleep (5). A meta-analysis of over 65 studies reflecting 3577 subjects across the lifespan reported a complex pattern of changes, including an increase of stage 1 but a decrease of stage 2 sleep in old age, as well as a decrease in REM sleep (6). An epidemiological investigation of self-reported sleep in older adults observed marker sex differences in age-related sleep changes, with females more likely to report disturbed sleep onset but men reporting night-time awakenings (7). Other findings age-related physiological changes in the alignment of homeostatic and circadian rhythms (8), decreases in sleep efficiency (9) the amount of slow-wave sleep, and an increase in daytime napping (10). Importantly, interruption and loss of sleep has been shown to have wide ranging adverse effects on health (11), leaving open the possibility that age-related changes in sleep patterns and quality may contribute to well-documented age-related declines in various health domains.

In the current study, we examine self-reported sleep habits in a large, population-based cohort Cambridge Centre for Ageing and Neuroscience (Cam-CAN (12)). We relate sleep measures to measures of health across four health domains: cognitive, brain health, physical and mental health. Our goal is to quantify and compare the associations between typical age-related changes in sleep quality and a range of measures of health measures that commonly decline in later life. We assess sleep using a self-reported measure of sleep quality, the Pittsburgh Sleep Quality Index (PSQI) (13). The PSQI has good psychometric properties (14) and has been shown to correlate reliably with diseases of aging and mortality (15–17). Although polysomnography (18) is commonly considered the gold standard of sleep quality measurement, it is often prohibitively challenging to employ in

For peer review only

For peer review only - http://bmjopen.bmj.com/site/about/guidelines.xhtml

large samples. A recent direct comparison of sleep measures (19) suggests that although subjective sleep measures (such as PSQI) may have certain drawbacks in older samples, they also capture complementary aspects of sleep quality not fully captured by polysomnography. Moreover, collecting self-report sleep quality data in a large, deeply phenotyped cohort offers several additional benefits.

By utilising a population cohort of healthy adults, and studying a range of health outcomes in the same population, we can circumvent challenges associated with studying clinical populations and provide new insights. First and foremost, by investigating associations between sleep and outcomes across multiple health domains in the same sample, we can make direct comparisons of the relative magnitude of these effects. Second, larger samples allow us to can generate precise effect size estimates, as well as adduce in favour of the null hypothesis. Third, we investigate the associations between sleep quality and neural health in a uniquely large healthy population. Previous investigations of the consequences of poor sleep on especially neural health have generally focuses on clinical populations such as those suffering from insomnia (20,21). Although such studies are crucial for understanding pathology, the demographic idiosyncrasies and often modest sample sizes of these approaches make it hard to generalize to healthy, community dwelling lifespan populations. Moreover, most studies that study age-related changes or differences focus on (very) old age, while far less is known about young and middle aged adults (6). For these reasons, our focus on a healthy, multimodal lifespan cohort is likely to yield novel insights into the subtle changes in sleep quality across the lifespan.

We will focus on three questions within each health domain: First, is there a relationship between sleep quality and health? Second, does the strength and nature of this relationship change when age is included as a covariate? Third, does the strength and nature of the relationship change across the lifespan? We will examine these questions across each of the four health domains.

For peer review only

METHODS

Sample

A cohort of 2544 (12) was recruited as part of the population-based Cambridge Centre for Ageing and Neuroscience (Cam-CAN) cohort (www.cam-can.com), drawn from the general population via Primary Care Trust (PCT)'s lists within the Cambridge City (UK) area 10,520 invitation letters were sent between 2010 and 2012, and willing participants were invited to have an interview conducted in their home, with questions on health, lifestyle demographics and core cognitive assessments. Sample size was chosen to allow for 100 participants per decile in further acquisition stages, giving sufficient power to separate age-related change from other sources of individual variation. For additional details of the project protocol see (12,22) and for further details of the Cam-CAN dataset visit http://www.mrc-cbu.cam.ac.uk/datasets/camcan/. A further subset of participants who were MRI compatible with no serious cognitive impairment participated in a neuroimaging session (22) between the 2011 and 2013. Participants included were native English speakers, had normal or corrected to normal vision and hearing, scored 25 or higher on the mini mental state (23). Note that other, more stringent cut-offs are sometimes employed to screen for premorbid dementia, such as a score of 88 or higher in the Addenbrookes Cognitive Examination – Revised (24). For the sake of comprehensiveness we repeated our analyses using this more stringent cut off (ACE-R>88), but observed no noteworthy differences in our findings, so we only report the findings based on the MMSE. Ethical approval for the study was obtained from the Cambridgeshire 2 (now East of England-Cambridge Central) Research Ethics Committee (reference: 10/H0308/50). Participants gave written informed consent. The raw data and analysis code are available upon signing a data sharing request form (see http://www.mrc-cbu.cam.ac.uk/datasets/camcan/ for more detail).

Variables

Sleep Measures

For peer review only

For peer review only - http://bmjopen.bmj.com/site/about/guidelines.xhtml

Sleep quality was assessed using the Pittsburgh Sleep Quality Index (PSQI), a well-validated self-report questionnaire (13,19) designed to assist in the diagnosis of sleep disorders. The questions concern sleep patterns, habits, and lifestyle questions, grouped into seven components, each yielding a score ranging from 0 (good sleep/no problems) to 3 (poor sleep/severe problems), that are commonly summed to a PSQI Total score ranging between 0 and 21, with higher scores reflecting poorer sleep quality.

Health Measures

Cognitive health. A number of studies have found associations between poor sleep and cognitive decline, including in elderly populations. Poor sleep affects cognitive abilities such as executive functions (25) and learning and memory processes (26), whereas short term pharmaceutical interventions such as administration of melatonin improve both sleep quality and cognitive performance (27,28). Recent work (29) concluded that "maintaining good sleep quality, at least in young adulthood and middle age, promotes better cognitive functioning and serves to protect against age-related cognitive declines". As sleep may affect various aspects of cognition differently (30), we include measures that cover a range of cognitive domains including memory, reasoning, response speed, and verbal fluency, as well as including a measure of general cognition (See Table 1 and (12) for more details).

Neural health. Previous research suggests that individuals with a severe disruption of sleep are significantly more likely to exhibit signs of poor neural health (20,31). Specifically, previous studies have observed decreased white matter health in clinical populations suffering from conditions such as chronic insomnia (21), obstructive sleep apnoea (32,33), excessively long sleep in patients with diabetes (34), and REM Sleep Behaviour Disorder (35). Many of these studies focus on white matter hyperintensities (WMH), a measure of the total volume or number of (regions) showing low-level neural pathology (although some study grey matter, e.g. (36,37). White matter hyperintensities are often used as a clinical marker, as longitudinal increases in WMHs are associated with increased risk of stroke, dementia and death (38) and are more prevalent in patients

For peer review only

For peer review only - http://bmjopen.bmj.com/site/about/guidelines.xhtml

with pathological sleep problems (33,34). However, use of this metric in clinical cohorts largely leaves open the question of the impact of sleep quality on neural (white matter) health in non-clinical, healthy populations. To address this question, we use a more general indicator of white matter neural health; *Fractional Anisotropy* (FA). FA is associated with white matter integrity and myelination (39,40). We use FA as recent evidence suggests that WMHs represent the extremes (foci) of white matter damage, and that FA is able to capture the full continuum of white matter integrity (41). For more information regarding the precise white matter pipeline, see (12,22,42).

Physical health. Sleep quality is also an important marker for physical health, with poorer sleep being associated with conditions such as obesity, diabetes mellitus (43), overall health (11,44) and increased all-cause mortality (45,46). We focus on a set of variables that capture three types of health domains commonly associated with poor sleep: Cardiovascular health measured by pulse, systolic and diastolic blood pressure (47), self-reported health, both in general and for the past 12 months (48) and body-mass index (49).

Mental health. Previous work has found that disruptions of sleep quality are a central symptom of forms of psychopathology such as Major Depressive Disorder, including both hypersomnia and insomnia (44,50), and episodes of insomnia earlier greatly increased the risk of later episodes of major depression (51). Kaneita et al. (52) found a U-shaped association between sleep and depression, such that individuals regularly sleeping less than 6, or more than 8, hours were more likely to be depressed. Both depression (53) and anxiety (54,55) are commonly associated with sleep problems. To capture these dimensions we used both scales of the Hospital Anxiety and Depression Scale (HADS) (56), a widely used and standardized questionnaire that captures self-reported frequency and intensity of anxiety and depression symptoms.

Health	Task and Description	Variable	Descriptives	Citati

For peer review only - http://bmjopen.bmj.com/site/about/guidelines.xhtml

domain				on
Cognitive	Story Recall Immediate: Participants hear a short story and are asked to recall as accurately as possible.	Recall manually scored for similarity and precision (min=0, max=24)	N = 2379, M=13.14, SD=4.66, Range=(0-24)	(57)
Cognitive	Story Recall Delayed: Same as above but recall after 30 minute delay	Recall manually scored for similarity and precision (min=0, max=24)	N = 2366, M=11.47, SD=4.92, Range=(0-24)	(57)
Cognitive	Letter Fluency (phonemic fluency): Participants have one minute to generate as many words as possible beginning with the letter 'p'.	Total words generated (min=0,max=30)	N = 2360, M=25.38, SD=3.96, Range=(0-30)	(57)
Cognitive	Animal Fluency (semantic fluency): Participants have one minute to generate as many words as possible in the category 'animals'.	Total words generated (min=0,max=30)	N = 2346, M=25.85, SD=4.47, Range=(0-30)	(57)
Cognitive	Cattell Culture Fair: Test of fluid reasoning using four subtests (series completions, odd-one-out, matrices and topology)	Total correct summed across four subtests. Min=0, max=46	N = 658, M=31.8, SD=6.79, Range=(11-44)	(58)
Cognitive	Simple reaction time: Speed in a simple reaction time task	1/response time in seconds	N = 657, M=0.37, SD=0.08, Range=(0.24-0.93)	(12)
Cognitive	Addenbrookes Cognitive Examination, Revised: Screening test for dementia using seven subtests (orientation, attention and concentration, memory, fluency, language, visuospatial abilities, perceptual abilities)	Performance on multiple tests converted to min=0, max=100 range	N = 2406, M=89.25, SD=13.4, Range=(0-100)	(24)
Neural	White matter health: Measure of tract integrity using fractional anisotropy	Fractional Anisotropy (min=0, max=1, averaged across 10 tracts)	N = 641, M=0.5, SD=0.03, Range=(0.3-0.56)	(59)
Physical	Self-reported Health, in general: Participants use a 4-point scale to respond to the prompt "Would you say for someone of your age, your own health in general is..."	Score from 1 = Excellent to 4= Poor	N = 2404, M=2.02, SD=0.79, Range=(1-3)	(60)
Physical	Self-reported Health, last 12 months: Participants use a 3-point scale to respond to the prompt "Over the last twelve months would you say your health has on the whole been..."	Score from 1 = Good to 3= Poor	N = 2398, M=1.46, SD=0.71, Range=(1-3)	(60)

For peer review only

Physical	Systolic blood pressure	Mean systolic blood pressure in mmHg, averaged across three consecutive measurements	N = 577, M=120.11, SD=17, Range=(78.5-186)	
Physical	Diastolic blood pressure	Mean diastolic blood pressure in mmHg, averaged across three consecutive measurements	N = 577, M=73.14, SD=10.48, Range=(49-115.5)	
Physical	Resting pulse	Mean pulse in beats per minute, averaged across three consecutive measurements	N = 578, M=65.69, SD=10.5, Range=(40-110.5)	
Physical	Body Mass Index (BMI)	(weight in kg) / (height in m)^2	N = 584, M=25.77, SD=4.59, Range=(16.75-48.32)	(61)
Mental health	Anxiety Subscale (Hospital Anxiety and Depression Scale (HADS)): Participants response to seven questions about anxiety-related behaviours	Seven questions rated on 0 to 3 scale ('Often' to 'Very seldom'). Min=0, Max=21	N = 2393, M=5.17, SD=3.4, Range=(0-19)	(56)
Mental health	Depression Subscale (Hospital Anxiety and Depression Scale (HADS)): Participants response to seven questions about depression-related behaviours	Seven questions rated on 0 to 3 scale ('Often' to 'Very seldom'). Min=0, Max=21	N = 2373, M=3.32, SD=2.91, Range=(0-14)	

183

Table 1. Description of health variables across each of four domains (cognitive, neural, physical, mental). For each variable details are given including a description of the task it is derived from, relevant citations, a brief definition and descriptive statistics.

For peer review only - http://bmjopen.bmj.com/site/about/guidelines.xhtml

STATISTICAL ANALYSES

We examine whether self-reported sleep patterns change across the lifespan, both for the PSQI sum score and for each of the seven PSQI components. We then examine the relationships between the sleep quality and the four health domains in three ways: First, simple regression of the health outcome on sleep variables, to determine evidence for association between poor sleep quality and poor health outcomes. Second, we include age as a covariate. Finally, we include a (standard normal rescaled) continuous interaction term to examine whether there is evidence for a changing relationship between sleep and outcomes across the lifespan.

For all regressions we will use a default Bayesian approach advocated by (62–65) which avoids several well-documented issues with p-values (64), allows for quantification of null effects, and decreases the risk of multiple comparison problems (66). Bayesian regressions allows us to symmetrically quantify evidence in favour of, or against, some substantive model as compared to a baseline (e.g. null) model. This evidentiary strength is expressed as a Bayes Factor (67), which can be interpreted as the relative likelihood of one model versus another given the data and a certain prior expectation. A Bayes Factor of, e.g., 7, in favour of a regression model suggests that the data are seven times *more likely* under that model than an intercept only model for a given prior (for an empirical comparison of p-values and Bayes factors, see (65)). A heuristic summary of evidentiary interpretation can be seen in Figure 1.

[insert Figure 1 here]

We report log Bayes Factors for (very) large effects and regular Bayes Factors for smaller effects. To compute Bayes Factors we will use Default Bayes Factor approach for model selection (62,63) in the package BayesFactor (68) using the open source software package R (69). As previous papers report associations between sleep and outcomes ranging from absent to considerable in size we utilize the default, symmetric Cauchy prior with width $\frac{\sqrt{2}}{2}$ which translates to a 50% confidence that the true effect will lie between -.707 and .707. Prior to further analysis, scores on all outcomes were transformed to a standard normal distribution, and any scores exceeding a z-score of 4 or -4

For peer review only - http://bmjopen.bmj.com/site/about/guidelines.xhtml

were recoded as missing (aggregate percentage outliers across the four health domains: Cognitive, 0.41%, Mental, 0.16%, Neural, 0.37% Physical, 0.031%).

RESULTS

Age-related differences in sleep quality

First, we examined sleep changes across the lifespan by examining age-related differences in the PSQI sum score (N= 2178, M=5.16, SD=3.35, Range=0-19). Regressing the PSQI global score on age, (see Supplementary Figure 1) showed evidence for a positive relationship across the lifespan ($logBF_{10}$= 10.45). This suggests that on the whole, sleep quality decreases across the lifespan (note that *higher* PSQI scores correspond to worse sleep). Although we observe strong statistical evidence for an age-related difference ('Extreme' according to (70)) age explained only 1.11 % of the variance in the PSQI Total score. Next, we examined each of the seven components on age in the same manner. In Supplementary Figure 2 we see that that age has varying and specific effects on different aspects of sleep quality, and did not worsen uniformly across the lifespan. For example, we observed moderate evidence that sleep latency did not change across the lifespan (Sleep Latency, BF_{01}= 9.66, in favour of the null), Sleep Quality showed no evidence for either change or stasis (BF_{10}= 1.64) and one sleep component, Daytime Dysfunction, improved slightly across the lifespan (BF_{10}= 7.04). Medication). The strongest age-related decline is that of Efficiency, showing an R-squared of 6.6%.

Finally, we entered all seven components into a Bayesian multiple regression simultaneously, to examine to what extent they could, together, predict age. The best model included every component except Sleep Duration ($logBF_{10}$= 142.98). Interestingly, this model explained 13.66% of the variance in age, compared to 1.12% for the PSQI Total score, and 6.6% for the strongest single component (efficiency). This shows that lifespan changes in self-reported sleep are heterogeneous and partially independent, and that specific patterns and components need to be taken into account simultaneously to fully understand age-related differences in sleep quality. These

For peer review only - http://bmjopen.bmj.com/site/about/guidelines.xhtml

finding shows that neither the PSQI sum score nor the sleep components in isolation fully capture differences in sleep quality across the lifespan.

The analysis above suggests that conceptualizing 'poor sleep' as a single dimension does not reflect the subtleties in lifespan changes – An often computed sumscore changes little across the lifespan, whereas the totality of sleep symptoms shows far stronger, and more subtle, patterns. To better elucidate individual differences in sleep quality we next use *Latent Class Analysis* (71). This technique will allow us examine individual differences in sleep quality across the lifespan in more detail than afforded by simple linear regressions: Rather than examining continuous variation in sleep components, LCA classifies individuals into different *sleep types*, each associated with a distinct profile of 'sleep symptoms'. If there are specific constellations of sleep problems across individuals, we can quantify and visualize such sleep types.

To analyse the data in this manner, we binarized the responses on each component into 'good' (0 or 1) or 'poor' (2 or 3). Our measures of PSQI symptoms straddle the border between continuous and categorical – Although some are fully continuous (e.g. sleep latency) others are less so. For instance, although scored on a range of four several of the scales (such as Subjective Sleep quality) have implicitly binary response options of 'Very good' and 'fairly good' on the one hand and 'fairly bad' and 'very bad' on the other. As analytical work in psychometrics (72) suggests that likert-like graded scales can be treated as continuous only from five ordinal categories upwards, by fitting an LCA we are erring on the side of caution (although a latent profile analysis would likely give similar results). Note that although our analysis divides individuals into discrete classes with specific profiles, it is still possible to examine the conditional response likelihood of responding 'yes' to each symptom as a continuous metric (between 0 and 1) that reflects the nature of the association between the class and the outcome. By modelling sleep 'types' we hope to illustrate the complex patterns in a more intelligible manner – notably, doing so allows us to examine whether the likelihood of belonging to any sleep 'type' changes as a function of age.

For peer review only - http://bmjopen.bmj.com/site/about/guidelines.xhtml

260 Next we examined evidence for distinct sleep types using We fit a set of possible models (varying from 2 to 6 sleep types) We found that the four class solution gives the best solution, according to the Bayesian Information Criterion (73) (BIC for 4 Classes = 11874.67, lowest BIC for other solutions= 11892.17 (5 classes) (with 50 repetitions per class, at 5000 maximum iterations). Next we inspected the nature of the sleep types, the prevalence of each 'sleep type' in the population, and whether the likelihood of belonging to a certain sleep type changes across the lifespan. See Figure 2 for the component profiles of the four sleep types identified.

[insert Figure 2 here]

Class 1, 'Good sleepers', make up 68.62% of participants. Their sleep profile is shown in Figure 2A, top left, and is characterised by a low probability of responding 'poor' to any of the sleep components. Class 2, 'inefficient sleepers', make up 13.05% of the participants, and are characterized by poor sleep Efficiency: Members of this group uniformly (100%) report poor sleep Efficiency, despite relatively low prevalence of other sleep problems, as seen in Figure 2A, top right. Class 3, 'Delayed Sleepers' seen in the bottom left of Figure 2a, makes up 9.76% of the participants: characterized by modestly poor sleep across the board, but a relatively high probability of poor scores on Sleep Latency (60%), Sleep Quality (54%) and sleep Disturbance (29.2%). Finally, Class 4, 'Poor sleepers', make up 8.6% of the participants, shown bottom right in Figure 2A. Their responses to any of the seven sleep components are likely to be 'poor' or 'very poor', almost universally so for 'sleep quality' (97%) and 'Sleep Efficiency' (96.6%).

Next, we including age as a covariate (simultaneously including a covariate is known as *latent class regression* or concomitant-variable latent class models (74). This analysis, visualised in Figure 2b, shows that the probability of membership of each classes compared to the reference class (good sleepers) changes significantly across the lifespan for each of the classes (Class 2 versus class 1: beta/SE= 0.054/0.0069, t=7.9, Class 3 versus class 1: beta/SE= -0.020/0.0057, t=-3.63, Class 4 versus class 1: beta/SE 0.015/0.0049, t=3.05), for more details on generalized logit coefficients , see (71). The frequency of Class 1 (Good sleepers) peaks in middle to late adulthood, dropping

For peer review only - http://bmjopen.bmj.com/site/about/guidelines.xhtml

increasingly quickly after age 50. Class 2 (Inefficient sleepers) are relatively rare in younger individuals, but the prevalence increases rapidly in individuals over age 50. On the other hand, Class 3 (Delayed sleepers) shows a steady decrease in the probability of an individual showing this profile across the lifespan, suggesting that this specific pattern of poor sleep is more commonly associated with younger adults. Finally, the proportion of Class 4 (poor sleepers) members increases only slightly across the lifespan. Together, the latent class analysis provides additional evidence that the PSQI sum score as an indicator of sleep quality does not fully capture the subtleties of age-related differences. Age-related changes in sleep patterns are characterized by specific, clustered patterns of sleep problems that cannot be adequately characterized by summation of the component scores. The above analyses show how both a summary measure and individual measures of sleep quality change across the lifespan. Next, we examined the relationships between sleep quality measures (seven components and the global PSQI score) and health variables (specific variables across four domains, as shown in Table 1).

Sleep, health domains and age

Cognitive health

First, we examined the relationships between sleep quality and seven measures of cognitive health (see Table 1 for details). We visualize our findings using tileplots (75). Each cell shows the numeric effect size (R-squared, 0-100) of the bivariate association between a sleep component and a health outcome, colour coded by the statistical evidence for a relationship using the Bayes Factor. If the parameter estimate is positive, the r-squared value has the symbol '+' added (note the interpretation depends on the nature of the variable, cf. Table 1).

As can be seen in Supplementary Figure 3, several relationships exist between measures of cognitive health and measures of sleep quality. However, these results attenuate in a multiple regression model including age as shown in Figure 3.

[Insert Figure 3 here]

For peer review only - http://bmjopen.bmj.com/site/about/guidelines.xhtml

For peer review only

The cognitive abilities most strongly associated with poor sleep are a measure of general cognitive health, ACE-R, and a test of verbal phonemic fluency. Two patterns emerged: First, the strongest predictor across the simple and multiple regressions was for the PSQI Total score. Tentatively this suggests that a cumulative index of sleep problems, rather than any specific pattern of poor sleep, is the biggest risk factor for poorer cognitive performance. Secondly, after controlling for age, the most strongly affected cognitive measure is phonemic fluency, the ability to generate name as many different words as possible starting with a given letter within a minute. Verbal fluency is commonly used as a neuropsychological test (76). Previous work suggests it depends on both the ability to cluster (generating words within a semantic cluster) and to switch (switching between categories), and is especially vulnerable to frontal and temporal lobe damage (with specific regions dependant on either a semantic or phonemic task (77)). Although modest in size, our findings suggests this task, dependent on multiple executive processes, is particularly affected by poor sleep quality (78). The second strongest association was with the ACE-R, a general cognitive test battery similar in style and content to the MMSE. When an interaction term with age was included, little evidence for interactions with age (mean $logBF_{10}$=-2.09, see Supplementary Figure 4), suggesting that the negative associations between sleep and cognitive performance are a constant feature across the lifespan, rather than specifically in elderly individuals. Together this suggests that poor sleep quality is modestly but consistently associated with poorer general cognitive performance across the lifespan, most strongly with semantic fluency.

Neural Health

Using Diffusion Tensor Imaging, we estimated a general index of white matter integrity in 10 tracts (59) (shown in Supplementary Figure 5), by taking the average Fractional Anisotropy in each white matter ROI (see (79) for more information). We use the data from a subsample of 641 individuals (age M=54.87, range 18.48-88.96) who were scanned in a 3T MRI scanner (for more details regarding the pipeline, sequence and processing steps, see (22,79). Regressing neural WM ROI's on sleep

For peer review only

For peer review only - http://bmjopen.bmj.com/site/about/guidelines.xhtml

quality, we find several small effects, with the strongest associations between sleep efficiency and neural health (see Supplementary Figure 6). All effects are such that poorer sleep is associated with poorer neural health, apart from a small effect in the opposite direction for Uncinate and Daytime Dysfunction (BF_{10}= 6.20). However, when age is included as a covariate, the negative associations between sleep quality and white matter health are attenuated virtually to zero (Figure 4, mean/median BF_{10}= 0.18/.10), with Bayes Factors providing strong evidence for the lack of associations between sleep quality and white matter integrity. One exception was observed: The use of Sleep Medication is associated with *better* neural health in the corticospinal tract, a region previously found to be affected by pathological sleep problems such as sleep apnoea (33). However, this effect is very small (BF_{10}=3.24) given the magnitude of the sample and the range of comparisons, so should be interpreted with caution.

[Insert Figure 4 here]

Finally, we tested for any interactions by including a mean-scaled interaction term (sleep*age, Supplementary Figure 7). This analysis found evidence for a significant interaction, between the Superior Longitudinal Fasciculus (SLF) and Sleep Medication (BF_{10}= 13.77), such that better neural health in the SLF was associated with the use of Sleep Medication more strongly in older adults. Together, these findings suggest that in general, once age is taken into account, self-reported sleep problems in a non-clinical sample are *not* associated with poorer neural health, although there is some evidence for a modest associations between better neural health in specific tracts and the use of sleep medication in the elderly.

Physical health

Next we examined whether sleep quality is associated with physical health. Figure 5 shows the simple regressions between sleep quality and physical health. Strong associations were found between poor overall sleep (PSQI sum score) and poor self-reported health, both in general ($logBF_{10}$=77.51) and even more strongly for health in the past 12 months ($logBF_{10}$=91.25). This may

For peer review only - http://bmjopen.bmj.com/site/about/guidelines.xhtml

be because poorer sleep, across all components, directly affects general physical health (43,80) or because people subjectively experience sleep quality as a fundamental part of overall general health. A second association was between BMI and poor sleep, most strongly for Duration ($logBF_{10}$=4.69).

[Insert Figure 5 here]

This not only replicates previous findings but is in line with an increasing body of evidence that suggests that shorted sleep duration causes metabolic changes, which in turn increases the risk of both diabetes mellitus and obesity (43,81,82). Next, we examined whether these effects were attenuated once age was included. We show that although the relationships are slightly weaker, the overall pattern remains (Supplementary Figure 8), suggesting these associations are not merely co-occurences across the lifespan. Our findings suggest self-reported sleep quality, especially sleep Duration, is related to differences in physical health outcomes in a healthy sample.

Finally, there was evidence of a single interaction with age (Supplementary Figure 9): Although poor sleep Duration was associated with *higher* diastolic blood pressure in younger adults, it was associated with *lower* diastolic blood pressure in older individuals (BF_{10}= 8.43). This may reflect the fact that diastolic blood pressure is related to cardiovascular health in a different way across the lifespan, although given the small effect size it should be interpreted with caution.

Mental health

Finally, we examined the relationship between sleep quality and mental health, as measured by the Hospital Anxiety and Depression Scale (56). One benefit of the HADS in this context is that, unlike some other definitions (e.g. the DSM-V), sleep quality is not an integral (scored) symptom of these dimensions. As shown in Supplementary Figure 10, there are very strong relationships between all aspects of sleep quality and measures of both anxiety and depression. The strongest predictors of Depression are Daytime Dysfunction ($logBF_{10}$= 245.9, R^2=19.26%), followed by the overall sleep score ($logBF_{10}$= 170.5, R^2=14.92%) and sleep quality ($logBF_{10}$= 106.8, R^2=8.9%). The effects size for Anxiety was comparable but slightly smaller in magnitude. When age is included as a covariate the

For peer review only - http://bmjopen.bmj.com/site/about/guidelines.xhtml

relationships remained virtually unchanged (Supplementary Figure 11), suggesting these relationships are present throughout across the lifespan. These findings replicate and extend previous work, suggesting that sleep quality is strongly associated with both anxiety and depression across the lifespan.

Finally we examined a model with an interaction term (Supplementary Figure 12). Most prominently we found interactions with age in the relationship between HADS depression and the PSQI Total, and in the relationship between HADS depression and Sleep Duration, such that for the relationship between anxiety and overall sleep quality is stronger in younger adults (BF_{10} =9.91, see Figure 6). Together our findings show that poor sleep quality is consistently, strongly and stably associated with poorer mental health across the adult lifespan.

[Insert Figure 6 here]

Non-linear associations between sleep and health outcomes

In the above analyses, we focused on linear associations between symptoms and health outcomes. However, for one aspect of sleep, namely sleep duration (in hours), evidence exists that these associations are likely to be non-linear, such that both shorter and longer than average sleep are associated with poorer health outcomes (e.g. (83–85). This is echoed in clinical criteria for depression, which commonly include that include both hyper- and hypo-somnia as 'sleep disruption' symptoms – In other words, both too much or too little sleep are suboptimal. To examine whether we observe evidence for non-linearities we examined the relationship between raw scores on sleep duration (in hours, not transformed to PSQI norms) and health outcomes across the four domains. If the association between sleep and outcomes is indeed u-shaped (or inverted U, depending on the scale) then a Bayesian regression would prefer the less parsimonious model that includes the quadratic term. We observed no non-linear associations between any neural or cognitive health variables. We find strong evidence for a quadratic (subscript q) over a linear (subscript l) associations between sleep duration and HADS anxiety ($logBF_{ql}$= 19.98), even more strongly so with HADS

For peer review only - http://bmjopen.bmj.com/site/about/guidelines.xhtml

Depression (logBF_{ql}= 25.83, see Figure 7A shows the strongest curvilinear association, namely with depression). We find a similar u-shaped curve with general health (BF_{ql}= 277.81) and self-reported health over the last 12 months (BF_{ql}=887.59), the latter shown in Figure 7b. Together, these analyses support previous conclusions that some (although not all) poorer health outcomes can be associated with both too much and too little sleep.

[Insert Figure 7 here]

DISCUSSION

In this study, we report on the associations between age-related differences in sleep quality and health outcomes in a large, age-heterogeneous sample of community dwelling adults of the Cambridge Neuroscience and Aging (Cam-CAN) cohort. We find that sleep quality generally decreases across the lifespan, most strongly for sleep Efficiency. However age-related changes in sleep patterns are complex and multifaceted, so we used Latent Class Analysis to identify 'sleep types' associated with specific sleep quality profiles. We found that Younger adults are more likely than older adults to display a pattern of sleep problems characterised by poor sleep quality and longer sleep latency, whereas older adults are more likely to display inefficient sleeping, characterised by long periods spent in bed whilst not asleep. Moreover, the probability of being a 'good' sleeper, unaffected by any adverse sleep symptoms, decreases considerably after age fifty.

Notably, closer investigation of the sleep classes reveals likely further complexities of age-related differences. The category 'poor sleepers', most prevalent in older adults, shows high conditional likelihood of 'poor sleep' across all symptoms except 'daytime dysfunction'. One possible explanation is that almost all individuals in this group are beyond retirement age. For this reason, they likely have greater flexibility in tailoring their day to day activities to their energy levels (as opposed to individuals working fulltime), and are therefore less likely to consider themselves 'disrupted' even in the presence of suboptimal sleep. Although more detailed, interview-based investigations would be necessary to examine the precise nature of these findings, it stands to reason that certain symptoms change not just in prevalence but also in meaning across the lifespan.

For peer review only - http://bmjopen.bmj.com/site/about/guidelines.xhtml

One key strength of our broad phenotypic assessment allows for direct comparison of the different measures of sleep quality and four key health domains. We find strongest associations between sleep quality and mental health, moderate relations between sleep quality and physical health and cognitive health and sleep, virtually all such that poorer sleep is associated with poorer health outcomes. We did not find evidence for associations between self-reported sleep and neural health. Notably, the relationships we observe are mostly stable across the lifespan, affecting younger and older individuals alike. A notable exception to these effects is the absence of any strong relation (after controlling for age) between sleep quality and neural health as indexed by tract-based average fractional anisotropy. Perhaps surprisingly, given we found strong relationships in the same sample between sleep and other outcomes (e.g. mental health, Figure 10) we find that self-reported sleep problems in a non-clinical sample are not associated with fractional anisotropy above and beyond old age. This is despite the fact that previous work within the same cohort observed moderate to strong associations between white matter and various cognitive outcomes (42,86,87). However, although notable, our finding does not rule out that such associations do exist with other white matter metrics, that they would be observed with objective measures of sleep such as polysomnography, or that the co-occurrence of age-related declines in sleep quality and white matter share an underlying causal association that cannot be teased apart in a cross-sectional sample.

One strength of our study is the assessment of neuroimaging metrics, namely fractional anisotropy, in a large, community-dwelling healthy population. Fractional anisotropy is often used in studies of aging (e.g. Madden, is relatively reliable (88)) and is sensitive to clinical anomalies such as white matter hyperintensities. However, the relationship between FA and white-matter health is indirect (40,89) and drawbacks include its inability to distinguish crossing fibers (e.g. (40,89) and vulnerability to movement and the fact that it likely reflects a combination of underlying physiological properties. Various alternative white matter metrics exist, including summary measures of diffusivity (e.g. axial/radial/mean diffusivity), volumetric measures of white matter

For peer review only - http://bmjopen.bmj.com/site/about/guidelines.xhtml

hyperintensity (e.g.) and various innovative measures currently in development, but their physiological validity is ongoing (89,90).

While there are limitations of self-report measures including in older cohorts (19), including the fact that they likely reflect different aspects of sleep health than polysomnography (sleep in the lab), our results suggest there are considerable advantages in using self-reported sleep measures: first, obtaining sleep quality data in a large and broadly phenotyped sample is feasible; and second, our results demonstrated clear and consistent associations across multiple domains for both subjective (e.g. self-reported health) and objective measures (e.g. memory tests, BMI), which both replicate and extend previous lab-based sleep findings. Future work should ideally simultaneously measure polysomnography and self-report in longitudinal, large scale cohorts to fully capture the range of overlapping and complementary relations between different aspects of sleep quality and health outcomes (19).

For both self-report and objective measures of sleep quality an open question is that of causality: Does poor sleep affect health outcomes, do health problems affect sleep, are they both markers of some third problem, or do causal influences go both ways? Most likely, all these patterns occur to varying degrees. Previous studies have shown that sleep quality causally affects health outcomes such as diabetes (43) and memory consolidation (1) while other evidence suggests that depression directly affect sleep quality (91,92) and that damage to neural structures may affect sleep regulation (93). Although our findings are in keeping with previous findings, our cross-sectional sample cannot tease apart the causal direction of the observed associations, more work remains to be done to disentangle these complex causal pathways.

In our paper we focus on a healthy, age-heterogeneous community dwelling sample. This allows us to study the associations between healthy aging and self-reported sleep quality, but comes with two key limitations of the interpretations of our findings. First and foremost, our findings are cross-sectional, not longitudinal. This means we can make inferences about age-related *differences*, but not necessarily age-related *changes* (94,95). One reason why cross-sectional and longitudinal

For peer review only - http://bmjopen.bmj.com/site/about/guidelines.xhtml

estimates may diverge is that older adults can be thought of as cohorts that differ from the younger adults in more ways than age alone. For example, our age range includes individuals born in the twenties and thirties of the 20th century. Compared to someone born in the 21st century, these individuals will likely have experience various differences during early life development (e.g. less broadly accessible education, lower quality of healthcare, poorer nutrition and similar patterns). For some of our measures, these are inherent limitations –*truly* longitudinal study of neural aging is inherently impossible as scanner technology has not been around sufficiently long. This means our findings likely reflect a combination of effects attributable to age-related changes as well as baseline differences between subpopulations that may affect both mean differences as well as developmental trajectories.

Second, our sample reflects an atypical population in the sense that they are willing and able to visit the laboratory on multiple occasions for testing sessions. This subsample is likely a more healthy subset of the full population, which will mean the range of (poor) sleep quality as well as (poorer) health outcomes will likely be less extreme that in the full population. However, this challenge is not specific to our sample. In fact, as the Cam-CAN cohort was developed using stratified sampling based on primary healthcare providers, our sample is likely as population-representative as is feasible for a cohort of this magnitude and phenotypic breadth (see (12) for further details). Nonetheless, a healthier subsample may lead to restriction of range (96), i.e. an attenuation of the strength of the associations observed between sleep quality and health outcomes. Practically, this means that our results likely generalise to comparable, healthy community dwelling adults, but not necessarily to populations that include those affected by either clinical sleep deprivation or other serious health conditions.

Conclusions

For peer review only

Taken together, our study allows several conclusions. First, although we replicate the age-related deterioration in some aspects of sleep quality, other aspects remain stable or even improve. Second, we show that the profile of sleep quality changes across the lifespan. This is important methodologically, as it suggests that PSQI sum scores do not capture the full picture, especially in age-heterogeneous samples. Moreover, it is important from a psychological standpoint: We show that ‘sleep quality’ is a multidimensional construct and should be treated as such if we wish to understand the complex effects and consequences of sleep quality across the lifespan. Third, moderate to strong relations exist between sleep quality and cognitive, physical and mental health, and these relations largely remain stable across the lifespan. In contrast, we show evidence that in non-clinical populations, poorer self-reported sleep is not reliably associated with poorer neural health. Finally, we find that for absolute sleep duration, we replicate previous findings that both longer and shorter than average amounts of sleep are association with poorer self-reported general health and higher levels of depression and anxiety.

Together with previous experimental and longitudinal evidence, our findings suggest that at least some age-related decreases in health outcomes may be due to poorer sleep quality. We show that self-reported sleep quality can be an important indicator of other aspects of healthy functioning throughout the lifespan, especially for mental and general physical health. Our findings suggest accurate understanding of sleep quality is essential in understanding and supporting healthy aging across the lifespan.

Author contributions

For peer review only - http://bmjopen.bmj.com/site/about/guidelines.xhtml

AG, MS and MS designed the study. AG and RAK performed the analyses. CC organized and conducted the data collection. AG, MS and RAK wrote the manuscript. YL provided considerable expertise on sleep and poor sleep outcomes. All authors approved the final manuscript.

Acknowledgements

The Cambridge Centre for Ageing and Neuroscience (Cam-CAN) research was supported by the Biotechnology and Biological Sciences Research Council (grant number BB/H008217/1). RAK is supported by the Sir Henry Wellcome Trust (grant number 107392/Z/15/Z) and the by UK Medical Research Council Programme (MC-A060-5PR61).

We would like to thank Richard Morey and Eric-Jan Wagenmakers for valuable suggestions regarding the use of the BayesFactor package. We are grateful to the Cam-CAN respondents and their primary care teams in Cambridge for their participation in this study. We also thank colleagues at the MRC Cognition and Brain Sciences Unit MEG and MRI facilities for their assistance. The Cam-CAN corporate author consists of the project principal personnel: Lorraine K Tyler, Carol Brayne, Edward T Bullmore, Andrew C Calder, Rhodri Cusack, Tim Dalgleish, John Duncan, Richard N Henson, Fiona E Matthews, William D Marslen-Wilson, James B Rowe, Research Associates: Karen Campbell, Teresa Cheung, Simon Davis, Linda Geerligs, Anna McCarrey, Abdur Mustafa, Darren Price, David Samu, Jason R Taylor, Matthias Treder, Kamen Tsvetanov, Janna van Belle, Nitin Williams; Research Assistants: Lauren Bates, Tina Emery, Sharon Erzinçlioglu, Sofia Gerbase, Stanimira Georgieva, Claire Hanley, Beth Parkin, David Troy; Affiliated Personnel: Tibor Auer, Marta Correia, Lu Gao, Emma Green, Rafael Henriques; Research Interviewers: Jodie Allen, Gillian Amery, Liana Amunts, Anne Barcroft, Amanda Castle, Cheryl Dias, Jonathan Dowrick, Melissa Fair, Hayley Fisher, Anna Goulding, Adarsh Grewal, Geoff Hale, Andrew Hilton, Frances Johnson, Patricia Johnston, Thea Kavanagh-Williamson, Magdalena Kwasniewska, Alison McMinn, Kim Norman, Jessica Penrose, Fiona Roby, Diane Rowland, John Sargeant, Maggie Squire, Beth Stevens, Aldabra Stoddart, Cheryl Stone, Tracy

For peer review only

For peer review only - http://bmjopen.bmj.com/site/about/guidelines.xhtml

Thompson, Ozlem Yazlik; and administrative staff: Dan Barnes, Marie Dixon, Jaya Hillman, Joanne Mitchell, Laura Villis.

For peer review only

For peer review only - http://bmjopen.bmj.com/site/about/guidelines.xhtml

References

1. Stickgold R. Sleep-dependent memory consolidation. Nature [Internet]. 2005 Oct 27 [cited 2014 Jul 10];437(7063):1272–8. Available from: http://dx.doi.org/10.1038/nature04286

2. Inoué S, Honda K, Komoda Y. Sleep as neuronal detoxification and restitution. Behav Brain Res. 1995 Jul;69(1–2):91–6.

3. Xie L, Kang H, Xu Q, Chen MJ, Liao Y, Thiyagarajan M, et al. Sleep drives metabolite clearance from the adult brain. Science [Internet]. NIH Public Access; 2013 Oct 18 [cited 2014 Jul 11];342(6156):373–7. Available from: http://europepmc.org/articles/PMC3880190/?report=abstract

4. D'Ambrosio C, Redline S. Impact of Sleep and Sleep Disturbances on Obesity and Cancer. Redline S, Berger NA, editors. New York, NY: Springer New York; 2014.

5. Crowley K. Sleep and Sleep Disorders in Older Adults. Neuropsychol Rev [Internet]. Springer US; 2011 Mar 12 [cited 2017 Feb 10];21(1):41–53. Available from: http://link.springer.com/10.1007/s11065-010-9154-6

6. Ohayon MM, Carskadon MA, Guilleminault C, Vitiello M V. Meta-analysis of quantitative sleep parameters from childhood to old age in healthy individuals: developing normative sleep values across the human lifespan. Sleep [Internet]. American Academy of Sleep Medicine; 2004 Nov 1 [cited 2017 Feb 10];27(7):1255–73. Available from: http://www.ncbi.nlm.nih.gov/pubmed/15586779

7. Middelkoop HAM, Smilde-van den Doel DA, Neven AK, Kamphuisen HAC, Springer CP. Subjective Sleep Characteristics of 1,485 Males and Females Aged 50-93: Effects of Sex and Age, and Factors Related to Self-Evaluated Quality of Sleep. Journals Gerontol Ser A Biol Sci Med Sci [Internet]. 1996 May 1 [cited 2015 Jun 22];51A(3):M108–15. Available from: http://biomedgerontology.oxfordjournals.org/content/51A/3/M108.short

8. Schmidt C, Peigneux P, Cajochen C. Age-related changes in sleep and circadian rhythms: impact on cognitive performance and underlying neuroanatomical networks. Front Neurol [Internet]. 2012 Jan [cited 2014 Jun 4];3:118. Available from: http://www.pubmedcentral.nih.gov/articlerender.fcgi?artid=3405459&tool=pmcentrez&rendertype=abstract

9. Leng Y, Wainwright NWJ, Cappuccio FP, Surtees PG, Luben R, Wareham N, et al. Self-reported sleep patterns in a British population cohort. Sleep Med [Internet]. 2014 Mar [cited 2016 Jan 28];15(3):295–302. Available from: http://www.sciencedirect.com/science/article/pii/S1389945714000185

10. Stanley N. The physiology of sleep and the impact of ageing. Eur Urol Suppl [Internet]. 2005 Jan [cited 2014 Sep 23];3(6):17–23. Available from: http://www.sciencedirect.com/science/article/pii/S156990560580003X

11. Briones B, Adams N, Strauss M, Rosenberg C, et al. Relationship between sleepiness and general health status. Sleep. 1996;19(7):583–8.

12. Shafto MA, Tyler LK, Dixon M, Taylor JR, Rowe JB, Cusack R, et al. The Cambridge Centre for Ageing and Neuroscience (Cam-CAN) study protocol: a cross-sectional, lifespan, multidisciplinary examination of healthy cognitive ageing. BMC Neurol [Internet]. BioMed Central; 2014 Jan 14 [cited 2015 May 20];14(1):204. Available from: http://bmcneurol.biomedcentral.com/articles/10.1186/s12883-014-0204-1

13. Buysse D, Reynolds C, Monk T, Berman S, Kupfer D. The Pittsburgh Sleep Quality Index: A new

For peer review only

For peer review only - http://bmjopen.bmj.com/site/about/guidelines.xhtml

instrument for Psychiatric Practise and Research .pdf. 1988. p. 193–213.

14. Carpenter JS, Andrykowski MA. Psychometric evaluation of the pittsburgh sleep quality index. J Psychosom Res [Internet]. 1998 Jul [cited 2015 Dec 10];45(1):5–13. Available from: http://www.sciencedirect.com/science/article/pii/S0022399997002985

15. Kang S-H, Yoon I-Y, Lee SD, Kim J-W. The impact of sleep apnoea syndrome on nocturia according to age in men. BJU Int [Internet]. 2012 Dec [cited 2015 Nov 25];110(11 Pt C):E851-6. Available from: http://www.ncbi.nlm.nih.gov/pubmed/22958406

16. Lou P, Qin Y, Zhang P, Chen P, Zhang L, Chang G, et al. Association of sleep quality and quality of life in type 2 diabetes mellitus: a cross-sectional study in China. Diabetes Res Clin Pract [Internet]. 2015 Jan [cited 2015 Nov 25];107(1):69–76. Available from: http://www.sciencedirect.com/science/article/pii/S0168822714004604

17. Mellor A, Waters F, Olaithe M, McGowan H, Bucks RS. Sleep and aging: examining the effect of psychological symptoms and risk of sleep-disordered breathing. Behav Sleep Med [Internet]. Routledge; 2014 Jan 28 [cited 2015 Nov 25];12(3):222–34. Available from: http://www.tandfonline.com/doi/abs/10.1080/15402002.2013.801343#.VlVu9HYrKHs

18. Kushida CA, Littner MR, Morgenthaler T, Alessi CA, Bailey D, Coleman J, et al. Practice Parameters for the Indications for PSG—AASM Practice Parameters Practice Parameters for the Indications for Polysomnography and Related Procedures: An Update for 2005. Sleep. 2005;28(4).

19. Landry GJ, Best JR, Liu-Ambrose T. Measuring sleep quality in older adults: a comparison using subjective and objective methods. Front Aging Neurosci [Internet]. Frontiers; 2015 Sep 7 [cited 2015 Sep 7];7. Available from: http://journal.frontiersin.org/article/10.3389/fnagi.2015.00166/abstract

20. Altena E, Vrenken H, Van der Werf YD, Heuvel OA van den H, Someren EJW van, van den Heuvel OA, et al. Reduced Orbitofrontal and Parietal Gray Matter in Chronic Insomnia: A Voxel-Based Morphometric Study [Internet]. Vol. 67, BIOL PSYCHIATRY. 2010 [cited 2014 Jul 3]. p. 182–185. Available from: http://www.sciencedirect.com/science/article/pii/S0006322309009548

21. Spiegelhalder K, Regen W, Prem M, Baglioni C, Nissen C, Feige B, et al. Reduced anterior internal capsule white matter integrity in primary insomnia. Hum Brain Mapp [Internet]. 2014 Jul 13 [cited 2015 Aug 4];35(7):3431–8. Available from: http://doi.wiley.com/10.1002/hbm.22412

22. Taylor JR, Williams N, Cusack R, Auer T, Shafto MA, Dixon M, et al. The Cambridge Centre for Ageing and Neuroscience (Cam-CAN) data repository: Structural and functional MRI, MEG, and cognitive data from a cross-sectional adult lifespan sample. Neuroimage [Internet]. 2015 Sep 12 [cited 2015 Sep 21]; Available from: http://www.sciencedirect.com/science/article/pii/S1053811915008150

23. Folstein MF, Folstein SE, McHugh PR. "Mini-mental state" a practical method for grading the cognitive state of patients for the clinician. J Psychiatr Res. 1975;12:189–98.

24. Mioshi E, Dawson K, Mitchell J, Arnold R, Hodges JR. The Addenbrooke's Cognitive Examination Revised (ACE-R): a brief cognitive test battery for dementia screening. Int J Geriatr Psychiatry [Internet]. 2006 Nov [cited 2015 Sep 29];21(11):1078–85. Available from: http://www.ncbi.nlm.nih.gov/pubmed/16977673

25. Regestein QR, Friebely J, Shifren JL, Scharf MB, Wiita B, Carver J, et al. Self-reported sleep in postmenopausal women. Menopause [Internet]. 2004 [cited 2015 Feb 17];11(2):198–207.

For peer review only - http://bmjopen.bmj.com/site/about/guidelines.xhtml

Available from: http://journals.lww.com/menopausejournal/Abstract/2004/11020/Self_reported_sleep_in_postmenopausal_women.12.aspx

26. Curcio G, Ferrara M, De Gennaro L. Sleep loss, learning capacity and academic performance. Sleep Med Rev [Internet]. 2006 Oct [cited 2015 Sep 15];10(5):323–37. Available from: http://www.sciencedirect.com/science/article/pii/S1087079205001231

27. Ferracioli-Oda E, Qawasmi A, Bloch MH, Hossain J, Shapiro C, Dikeos D, et al. Meta-Analysis: Melatonin for the Treatment of Primary Sleep Disorders. Romanovsky AA, editor. PLoS One [Internet]. Public Library of Science; 2013 May 17 [cited 2017 Jan 31];8(5):e63773. Available from: http://dx.plos.org/10.1371/journal.pone.0063773

28. Jean-Louis G, Gizycki H, Zizi F. Melatonin effects on sleep, mood, and cognition in elderly with mild cognitive impairment. J Pineal Res [Internet]. 1998 Nov [cited 2015 Jun 16];25(3):177–83. Available from: http://doi.wiley.com/10.1111/j.1600-079X.1998.tb00557.x

29. Scullin MK, Bliwise DL. Sleep, Cognition, and Normal Aging: Integrating a Half Century of Multidisciplinary Research. Perspect Psychol Sci. 2015 Jan;10(1):97–137.

30. Nebes RD, Buysse DJ, Halligan EM, Houck PR, Monk TH. Self-reported sleep quality predicts poor cognitive performance in healthy older adults. J Gerontol B Psychol Sci Soc Sci [Internet]. 2009 Mar [cited 2014 Sep 8];64(2):180–7. Available from: http://www.pubmedcentral.nih.gov/articlerender.fcgi?artid=2655169&tool=pmcentrez&rendertype=abstract

31. Kamba M, Inoue Y, Higami S, Suto Y, Ogawa T, Chen W. Cerebral metabolic impairment in patients with obstructive sleep apnoea: an independent association of obstructive sleep apnoea with white matter change. J Neurol Neurosurg Psychiatry [Internet]. 2001 Sep;71(3):334–9. Available from: http://www.pubmedcentral.nih.gov/articlerender.fcgi?artid=1737534&tool=pmcentrez&rendertype=abstract

32. Harbison J, Gibson GJ, Birchall D, Zammit-Maempel I, Ford GA. White matter disease and sleep-disordered breathing after acute stroke. Neurology [Internet]. 2003 Oct 13 [cited 2016 Jan 7];61(7):959–63. Available from: http://www.neurology.org/content/61/7/959.short

33. Macey PM, Kumar R, Woo M a, Valladares EM, Yan-Go FL, Harper RM. Brain structural changes in obstructive sleep apnea. Sleep [Internet]. 2008 Jul;31(7):967–77. Available from: http://www.ncbi.nlm.nih.gov/pubmed/21300501

34. Ramos AR, Dong C, Rundek T, Elkind MS V, Boden-Albala B, Sacco RL, et al. Sleep duration is associated with white matter hyperintensity volume in older adults: the Northern Manhattan Study. J Sleep Res [Internet]. 2014 Jul 7 [cited 2014 Sep 8];i. Available from: http://www.ncbi.nlm.nih.gov/pubmed/25040435

35. Unger MM, Belke M, Menzler K, Heverhagen JT, Keil B, Stiasny-Kolster K, et al. Diffusion tensor imaging in idiopathic REM sleep behavior disorder reveals microstructural changes in the brainstem, substantia nigra, olfactory region, and other brain regions. Sleep [Internet]. American Academy of Sleep Medicine; 2010 Jun 1 [cited 2015 Dec 28];33(6):767–73. Available from: /pmc/articles/PMC2881532/?report=abstract

36. Macey PM, Henderson LA, Macey KE, Alger JR, Frysinger RC, Woo MA, et al. Brain morphology associated with obstructive sleep apnea. Am J Respir Crit Care Med [Internet]. American Thoracic Society; 2002 Nov 15 [cited 2014 Nov 16];166(10):1382–7. Available from: http://www.atsjournals.org/doi/abs/10.1164/rccm.200201-050OC#.VGkRkfmsXIk

For peer review only - http://bmjopen.bmj.com/site/about/guidelines.xhtml

37. Sexton CE, Storsve AB, Walhovd KB, Johansen-Berg H, Fjell AM. Poor sleep quality is associated with increased cortical atrophy in community-dwelling adults. Neurology [Internet]. 2014 Sep 9 [cited 2016 Jun 21];83(11):967–73. Available from: http://www.ncbi.nlm.nih.gov/pubmed/25186857

38. Debette S, Markus HS. The clinical importance of white matter hyperintensities on brain magnetic resonance imaging: systematic review and meta-analysis. BMJ [Internet]. 2010 Jul 26 [cited 2016 Jan 12];341(jul26 1):c3666–c3666. Available from: http://www.bmj.com/content/341/bmj.c3666

39. Mädler B, Drabycz SA, Kolind SH, Whittall KP, MacKay AL. Is diffusion anisotropy an accurate monitor of myelination? Correlation of multicomponent T2 relaxation and diffusion tensor anisotropy in human brain. Magn Reson Imaging [Internet]. 2008 Sep [cited 2016 Jan 6];26(7):874–88. Available from: http://www.ncbi.nlm.nih.gov/pubmed/18524521

40. Jones DK, Knösche TR, Turner R. White matter integrity, fiber count, and other fallacies: The do's and don'ts of diffusion MRI. Vol. 73, NeuroImage. 2013. p. 239–54.

41. Maillard P, Fletcher E, Harvey D, Carmichael O, Reed B, Mungas D, et al. White matter hyperintensity penumbra. Stroke [Internet]. 2011 Jul 1 [cited 2016 Jan 6];42(7):1917–22. Available from: http://stroke.ahajournals.org/content/42/7/1917.short

42. Kievit RA, Davis SW, Griffiths J, Correia MM, Cam-CAN, Henson RN. A watershed model of individual differences in fluid intelligence. Neuropsychologia. 2016;91:186–98.

43. Spiegel K, Tasali E, Leproult R, Van Cauter E. Effects of poor and short sleep on glucose metabolism and obesity risk. Nat Rev Endocrinol [Internet]. Nature Publishing Group; 2009 May [cited 2015 Aug 4];5(5):253–61. Available from: http://dx.doi.org/10.1038/nrendo.2009.23

44. Grandner MA, Jackson NJ, Izci-Balserak B, Gallagher RA, Murray-Bachmann R, Williams NJ, et al. Social and Behavioral Determinants of Perceived Insufficient Sleep. Front Neurol [Internet]. Frontiers; 2015 Jan 5 [cited 2015 Aug 3];6:112. Available from: http://journal.frontiersin.org/article/10.3389/fneur.2015.00112/abstract

45. Leng Y, Wainwright NWJ, Cappuccio FP, Surtees PG, Hayat S, Luben R, et al. Daytime napping and the risk of all-cause and cause-specific mortality: a 13-year follow-up of a British population. Am J Epidemiol [Internet]. 2014 May 1 [cited 2014 Aug 27];179(9):1115–24. Available from: http://www.pubmedcentral.nih.gov/articlerender.fcgi?artid=3992821&tool=pmcentrez&rendertype=abstract

46. Leng Y, Cappuccio FP, Wainwright NWJ, Surtees PG, Luben R, Brayne C, et al. Sleep duration and risk of fatal and nonfatal stroke: a prospective study and meta-analysis. Neurology [Internet]. 2015 Mar 17 [cited 2016 Jan 28];84(11):1072–9. Available from: http://www.neurology.org/content/early/2015/02/25/WNL.0000000000001371.abstract

47. Hoevenaar-Blom MP, Spijkerman AMW, Kromhout D, van den Berg JF, Verschuren WMM. Sleep duration and sleep quality in relation to 12-year cardiovascular disease incidence: the MORGEN study. Sleep [Internet]. 2011 Nov [cited 2016 Jan 6];34(11):1487–92. Available from: http://www.pubmedcentral.nih.gov/articlerender.fcgi?artid=3198203&tool=pmcentrez&rendertype=abstract

48. Strine TW, Chapman DP. Associations of frequent sleep insufficiency with health-related quality of life and health behaviors. Sleep Med [Internet]. 2005 Jan [cited 2015 Oct 19];6(1):23–7. Available from:

For peer review only - http://bmjopen.bmj.com/site/about/guidelines.xhtml

http://www.sciencedirect.com/science/article/pii/S1389945704001078

49. Taheri S, Lin L, Austin D, Young T, Mignot E. Short sleep duration is associated with reduced leptin, elevated ghrelin, and increased body mass index. PLoS Med [Internet]. Public Library of Science; 2004 Dec 7 [cited 2015 Nov 1];1(3):e62. Available from: http://journals.plos.org/plosmedicine/article?id=10.1371/journal.pmed.0010062

50. Roberts RE, Shema SJ, Kaplan GA, Strawbridge WJ. Sleep Complaints and Depression in an Aging Cohort: A Prospective Perspective. Am J Psychiatry [Internet]. American Psychiatric Publishing; 2000 Jan 1 [cited 2015 Jun 16];157(1):81–8. Available from: http://ajp.psychiatryonline.org/doi/10.1176/ajp.157.1.81

51. Breslau N, Roth T, Rosenthal L, Andreski P. Sleep disturbance and psychiatric disorders: A longitudinal epidemiological study of young Adults. Biol Psychiatry [Internet]. 1996 Mar [cited 2015 Apr 8];39(6):411–8. Available from: http://www.sciencedirect.com/science/article/pii/0006322395001883

52. Kaneita Y, Ohida T, Uchiyama M, Takemura S, Kawahara K, Yokoyama E, et al. The Relationship Between Depression and Sleep Disturbances: A Japanese Nationwide General Population Survey. J Clin Psychiatry [Internet]. 2006 Feb [cited 2015 Jun 16];67(2):196–203. Available from: http://www.ncbi.nlm.nih.gov/pubmed/16566613

53. Fried EI, Nesse RM. Depression sum-scores don't add up: why analyzing specific depression symptoms is essential. BMC Med [Internet]. 2015 Apr 6 [cited 2015 Apr 9];13(1):72. Available from: http://www.biomedcentral.com/1741-7015/13/72

54. Novati A, Hulshof HJ, Koolhaas JM, Lucassen PJ, Meerlo P. Chronic sleep restriction causes a decrease in hippocampal volume in adolescent rats, which is not explained by changes in glucocorticoid levels or neurogenesis. Neuroscience [Internet]. 2011 Sep 8 [cited 2015 Jan 20];190:145–55. Available from: http://www.sciencedirect.com/science/article/pii/S0306452211007111

55. Ramsawh HJ, Stein MB, Belik S-L, Jacobi F, Sareen J. Relationship of anxiety disorders, sleep quality, and functional impairment in a community sample. J Psychiatr Res [Internet]. 2009 Jul [cited 2015 Dec 7];43(10):926–33. Available from: http://www.sciencedirect.com/science/article/pii/S0022395609000211

56. Zigmond AS, Snaith RP. The hospital anxiety and depression scale. Acta Psychiatr Scand [Internet]. 1983 Jun [cited 2014 Jul 11];67(6):361–70. Available from: http://www.ncbi.nlm.nih.gov/pubmed/6880820

57. Wechsler CJ. Wechsler Memory Scale. 3d UK. London: Harcourt; 1999.

58. Cattell RB. Abilities: their structure, growth, and action. Boston: Houghton-Mifflin; 1971.

59. Hua K, Zhang J, Wakana S, Jiang H, Li X, Reich DS, et al. Tract probability maps in stereotaxic spaces: analyses of white matter anatomy and tract-specific quantification. Neuroimage. 2008 Jan;39(1):336–47.

60. McGee DL, Liao Y, Cao G, Cooper RS. Self-reported Health Status and Mortality in a Multiethnic US Cohort. Am J Epidemiol [Internet]. 1999 Jan 1 [cited 2015 Nov 25];149(1):41–6. Available from: http://aje.oxfordjournals.org/content/149/1/41.short

61. Deurenberg P, Weststrate JA, Seidell JC. Body mass index as a measure of body fatness: age- and sex-specific prediction formulas. Br J Nutr [Internet]. Cambridge University Press; 2007 Mar 9 [cited 2015 Oct 7];65(2):105. Available from: http://journals.cambridge.org/abstract_S0007114591000193

For peer review only - http://bmjopen.bmj.com/site/about/guidelines.xhtml

62. Liang F, Paulo R, Molina G, Clyde MA, Berger JO. Mixtures of g Priors for Bayesian Variable Selection. J Am Stat Assoc [Internet]. Taylor & Francis; 2008 Mar 1 [cited 2015 Oct 7];103(481):410–23. Available from: http://amstat.tandfonline.com/doi/abs/10.1198/016214507000001337#.VhTxTPl3lpg

63. Rouder JN, Morey RD. Default Bayes Factors for Model Selection in Regression. Multivariate Behav Res [Internet]. Taylor & Francis Group; 2012 Nov 17 [cited 2015 Jun 16];47(6):877–903. Available from: http://www.tandfonline.com/doi/abs/10.1080/00273171.2012.734737

64. Wagenmakers E-J. A practical solution to the pervasive problems ofp values. Psychon Bull Rev [Internet]. 2007 Oct [cited 2015 Jun 16];14(5):779–804. Available from: http://www.springerlink.com/index/10.3758/BF03194105

65. Wetzels R, Matzke D, Lee MD, Rouder JN, Iverson GJ, Wagenmakers E-J. Statistical Evidence in Experimental Psychology: An Empirical Comparison Using 855 t Tests. Perspect Psychol Sci [Internet]. 2011 May 18 [cited 2015 May 12];6(3):291–8. Available from: http://pps.sagepub.com/content/6/3/291.short

66. Gelman A, Hill J, Yajima M. Why We (Usually) Don't Have to Worry About Multiple Comparisons. J Res Educ Eff [Internet]. Taylor & Francis Group; 2012 Apr 3 [cited 2014 Jul 15];5(2):189–211. Available from: http://www.tandfonline.com/doi/abs/10.1080/19345747.2011.618213

67. Jeffreys H. A theory of probability. Oxford: Oxford University Press; 1961.

68. Morey RD, Rouder JN. BayesFactor. CRAN; 2015.

69. Team. R: a language and environment for statistical computing. Vienna; 2013.

70. Jeffreys H. Theory of Probability. Oxford: Oxford University Press; 1961.

71. Linzer DA, Lewis JB. poLCA: An R Package for Polytomous Variable Latent Class Analysis [Internet]. Journal of Statistical Software. 2011 [cited 2014 Sep 8]. p. 42: 10. Available from: http://www.jstatsoft.org/v42/i10/paper

72. Rhemtulla M, Brosseau-Liard PÉ, Savalei V. When can categorical variables be treated as continuous? A comparison of robust continuous and categorical SEM estimation methods under suboptimal conditions. Psychol Methods [Internet]. American Psychological Association; 2012 [cited 2017 Feb 10];17(3):354–73. Available from: http://doi.apa.org/getdoi.cfm?doi=10.1037/a0029315

73. Schwarz G. Estimating the Dimension of a Model. Ann Stat [Internet]. Institute of Mathematical Statistics; 1978 Mar 1 [cited 2015 Jun 16];6(2):461–4. Available from: http://projecteuclid.org/euclid.aos/1176344136

74. Dayton CM, Macready GB. Concomitant-Variable Latent-Class Models. J Am Stat Assoc [Internet]. Taylor & Francis; 1988 Mar [cited 2014 Sep 8];83(401):173–8. Available from: http://www.tandfonline.com/doi/abs/10.1080/01621459.1988.10478584

75. Wickham H. ggplot2: Elegant Graphics for Data Analysis [Internet]. Springer Science & Business Media; 2009 [cited 2015 Aug 4]. 221 p. Available from: https://books.google.com/books?hl=en&lr=&id=bes-AAAAQBAJ&pgis=1

76. Miller E. Verbal fluency as a function of a measure of verbal intelligence and in relation to different types of cerebral pathology. Br J Clin Psychol [Internet]. 1984 Feb 12 [cited 2016 Jan 7];23(1):53–7. Available from: http://doi.wiley.com/10.1111/j.2044-8260.1984.tb00626.x

77. Biesbroek JM, van Zandvoort MJE, Kappelle LJ, Velthuis BK, Biessels GJ, Postma A. Shared and distinct anatomical correlates of semantic and phonemic fluency revealed by lesion-symptom

For peer review only - http://bmjopen.bmj.com/site/about/guidelines.xhtml

mapping in patients with ischemic stroke. Brain Struct Funct [Internet]. Springer Berlin Heidelberg; 2016 May 5 [cited 2017 Jan 31];221(4):2123–34. Available from: http://link.springer.com/10.1007/s00429-015-1033-8

78. Troyer AK, Moscovitch M, Winocur G. Clustering and switching as two components of verbal fluency: Evidence from younger and older healthy adults. Neuropsychology [Internet]. 1997 [cited 2016 Jan 7];11(1). Available from: http://psycnet.apa.orgjournals/neu/11/1/138

79. Kievit RA, Davis SW, Griffiths JD, Correia MM, Henson RNA. A watershed model of individual differences in fluid intelligence. bioRxiv [Internet]. Cold Spring Harbor Labs Journals; 2016 Feb 26 [cited 2016 Mar 29];41368. Available from: http://www.biorxiv.org/content/early/2016/02/26/041368.abstract

80. Briones B, Adams N, Strauss M, Rosenberg C, Whalen C, Carskadon M, et al. Relationship between sleepiness and general health status. Sleep [Internet]. 1996 Sep [cited 2015 Aug 4];19(7):583–8. Available from: http://www.ncbi.nlm.nih.gov/pubmed/8899938

81. Cizza G, Skarulis M, Mignot E. A link between short sleep and obesity : Building the evidence for causation. Sleep [Internet]. American Academy of Sleep Medicine; 2005 [cited 2016 Jan 12];28(10):1217–20. Available from: http://cat.inist.fr/?aModele=afficheN&cpsidt=17179376

82. Gangwisch JE, Malaspina D, Boden-Albala B, Heymsfield SB. Inadequate sleep as a risk factor for obesity: analyses of the NHANES I. Sleep [Internet]. 2005 Oct [cited 2015 Sep 3];28(10):1289–96. Available from: http://europepmc.org/abstract/med/16295214

83. Grandner MA, Drummond SPA. Who are the long sleepers? Towards an understanding of the mortality relationship. Sleep Med Rev. 2007;11(5):341–60.

84. KANEITA Y, UCHIYAMA M, YOSHIIKE N, OHIDA T. Associations of Usual Sleep Duration with Serum Lipid and Lipoprotein Levels. Sleep. American Academy of Sleep Medicine; 31(5):645–52.

85. Grandner MA, Hale L, Moore M, Patel NP. Mortality associated with short sleep duration: The evidence, the possible mechanisms, and the future. Sleep Med Rev. 2010;14(3):191–203.

86. Kievit RA, Davis SW, Mitchell D, Taylor JR, Duncan J, Cam-CAN, et al. Distinct aspects of frontal lobe structure mediate age-related differences in fluid intelligence and multitasking. Nat Commun. 2014;

87. Henson RN, Campbell KL, Davis SW, Taylor JR, Emery T, Erzinclioglu S, et al. Multiple determinants of lifespan memory differences. Sci Rep [Internet]. Nature Publishing Group; 2016 Sep 7 [cited 2017 Feb 10];6:32527. Available from: http://www.nature.com/articles/srep32527

88. Fox RJ, Sakaie K, Lee J-C, Debbins JP, Liu Y, Arnold DL, et al. A validation study of multicenter diffusion tensor imaging: reliability of fractional anisotropy and diffusivity values. AJNR Am J Neuroradiol [Internet]. American Society of Neuroradiology; 2012 Apr [cited 2017 Feb 10];33(4):695–700. Available from: http://www.ncbi.nlm.nih.gov/pubmed/22173748

89. Wandell BA. Clarifying Human White Matter. Annu Rev Neurosci [Internet]. Annual Reviews ; 2016 Jul 8 [cited 2017 Feb 10];39(1):103–28. Available from: http://www.annualreviews.org/doi/10.1146/annurev-neuro-070815-013815

90. Tournier J-D, Mori S, Leemans A. Diffusion tensor imaging and beyond. Magn Reson Med [Internet]. Wiley Subscription Services, Inc., A Wiley Company; 2011 Jun [cited 2017 Feb 10];65(6):1532–56. Available from: http://doi.wiley.com/10.1002/mrm.22924

91. Lustberg L, Reynolds CF. Depression and insomnia: questions of cause and effect. Sleep Med

For peer review only

For peer review only - http://bmjopen.bmj.com/site/about/guidelines.xhtml

Rev [Internet]. 2000 Jun [cited 2015 Dec 28];4(3):253–62. Available from: http://www.sciencedirect.com/science/article/pii/S1087079299900758

92. Sbarra DA, Allen JJB. Decomposing depression: On the prospective and reciprocal dynamics of mood and sleep disturbances.

93. Lim ASP, Ellison BA, Wang JL, Yu L, Schneider JA, Buchman AS, et al. Sleep is related to neuron numbers in the ventrolateral preoptic/intermediate nucleus in older adults with and without Alzheimer's disease. Brain [Internet]. 2014 Oct 20 [cited 2015 Dec 16];137(Pt 10):2847–61. Available from: http://brain.oxfordjournals.org/content/early/2014/08/11/brain.awu222

94. Raz N, Lindenberger U. Only time will tell: Cross-sectional studies offer no solution to the age–brain–cognition triangle: Comment on Salthouse (2011).

95. Schaie KW. The course of adult intellectual development.

96. Wiberg M, Sundstrom A. A Comparison of Two Approaches to Correction of Restriction of Range in Correlation Analysis. Pract Assessment, Res Eval [Internet]. Dr. Lawrence M. Rudner. e-mail: editor@pareonline.net; Web site: http://pareonline.net; 2009 Feb 28 [cited 2016 Feb 19];14(5). Available from: http://eric.ed.gov/?id=EJ933658

For peer review only

Legends

Figure 1. Descriptive interpretation of Bayes Factors

Figure 2. Latent Class Analysis. Panel A shows the sleep quality profiles for each of the four classes. Panel B shows the conditional probability of belonging to each class across the lifespan.

Figure 3. Simple regressions between sleep components and Cognitive Health.
The strength of the effect is colour-coded by Bayes Factor, and the effect size is shown as r-squared (as a percentage out of 100). Sample varies across components and measures due to varying missingness. Cattell and Reaction Time were measured only in the imaging cohort: mean N = 648, N=11.11. Sample sizes for 5 other domains are similar: mean N= 2300.25, SD= 65.57)

Figure 4. Multiple regressions between sleep components and Neural Health. Each cell represents the relationship between a sleep component and the mean neural health in a given tract as index by Fractional Anisotropy. Numbers represent R-squared, the sample size is show in the last column. Strong associations are observed between measures of Sleep Efficiency and multiple tracts, along with sporadic associations between other components and tracts. White matter tracts abbreviations: Uncinate fasciculus (UNC), superior longitudinal fasciculus (SLF), inferior longitudinal fasciculus (ILF), inferior Fronto-occipital fasciculus (IFOF), forceps minor (FMin), forceps major (FMaj), cerebrospinal tract (CST), the ventral cingulate gyrus (CINGHipp), the dorsal cingulate gyrus (CING), and the anterior thalamic radiations (ATR). N varies slightly across components due to varying missingness (N mean = 631.325, SD = 10.32).

Figure 5 Physical health and sleep quality. Numbers represent Rsquared, the sample size is show in the last column. Strong associations between general indices of health and sleep quality are found, and several more modest relationships with BMI and sleep quality. Self-reported health (12 month and General) were measured in the full cohort (Mean = 2315.37, SD=66.29), the other indicators were measured in the imaging cohort only (Mean = 569.87, SD= 11.16).

Figure 6. Interaction between sleep quality and anxiety. (N=724, age 18.48 to 46.2) compared to the oldest third of participants (N=725, age 71.79 to 98.88).

Figure 7. Curvilinear associations between sleep duration in hours and A) HADS depression and B) general health (self-reported). For visual clarity a small amount of random jitter was added to the data points.

For peer review only - http://bmjopen.bmj.com/site/about/guidelines.xhtml

Bayes Factor BF_{10}	Log BF_{10}	Tileplot colour	Description (Jeffreys, 1961)
>100	>4.6		Extreme evidence for H1
30 – 100	3.4 – 4-6		Very strong evidence for H1
10 – 30	2.3 – 3.4		Strong evidence for H1
3 – 10	1.098 – 2.3		Substantial evidence for H1
1 – 3	1 – 1.098		Anecdotal evidence for H1
1	0		No evidence either way
1/3 – 1	-1.098 – -1		Anecdotal evidence for H0
1/3 - 1/10	-2.3 – -1.098		Substantial evidence for H0
1/10 - 1/30	-3.4 – -2.3		Strong evidence for H0
1/30 - 1/100	-4.6 – - 3.4		Very strong evidence for H0
<1/100	< -4.6		Extreme evidence for H0

Figure 1. Descriptive interpretation of Bayes Factors

Insert Figure 1 here
338x190mm (96 x 96 DPI)

eview only

For peer review only - http://bmjopen.bmj.com/site/about/guidelines.xhtml

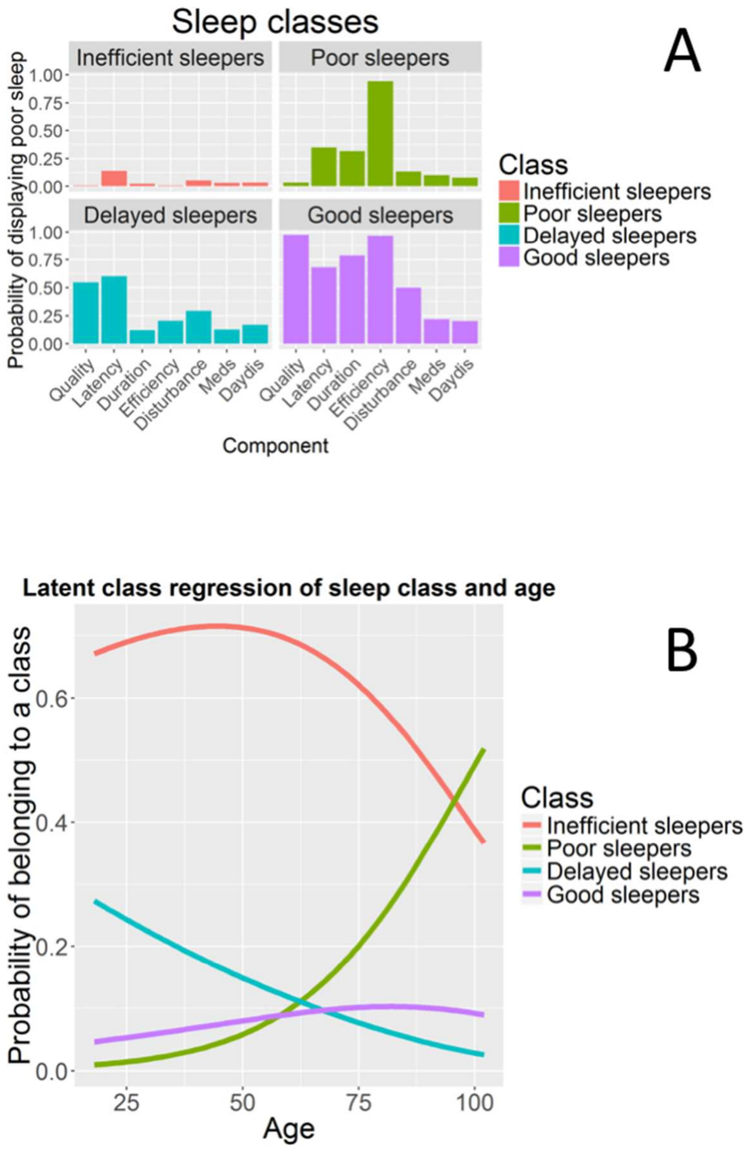

Figure 2. Latent Class Analysis. Panel A shows the sleep quality profiles for each of the four classes. Panel B shows the conditional probability of belonging to each class across the lifespan.

Insert Figure 2 here
60x89mm (300 x 300 DPI)

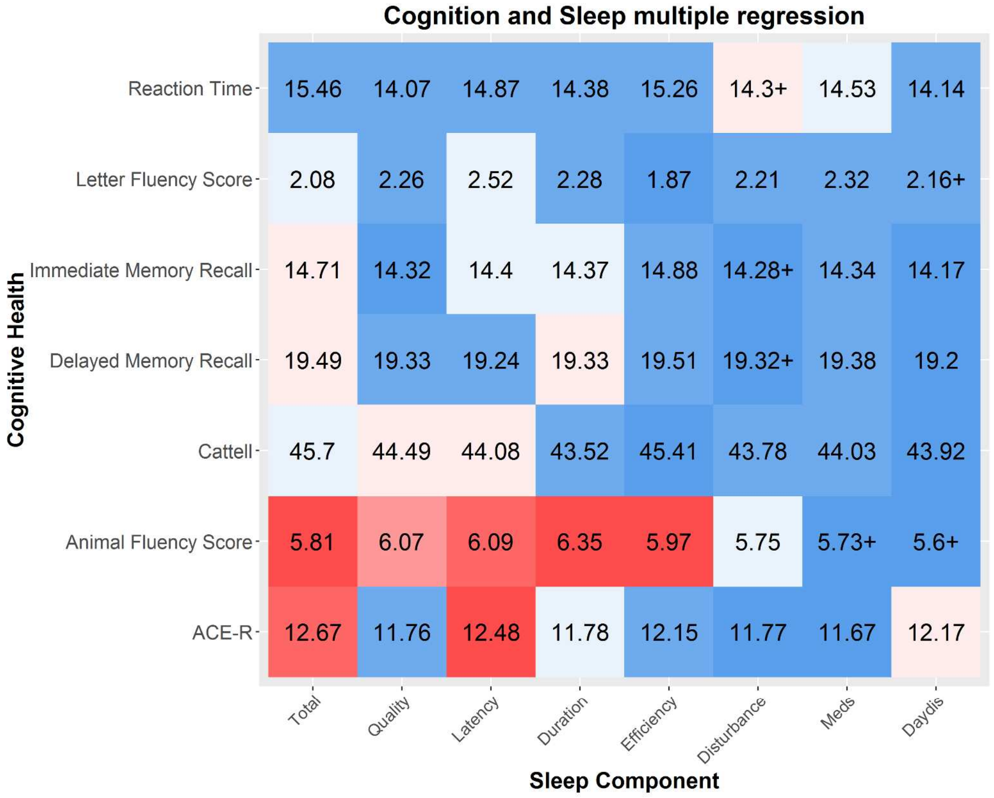

Figure 3. Simple regressions between sleep components and Cognitive Health.
The strength of the effect is colour-coded by Bayes Factor, and the effect size is shown as r-squared (as a percentage out of 100). Sample varies across components and measures due to varying missingness. Cattell and Reaction Time were measured only in the imaging cohort: mean N = 648, N=11.11. Sample sizes for 5 other domains are similar: mean N= 2300.25, SD= 65.57)

Insert Figure 3 here
254x203mm (300 x 300 DPI)

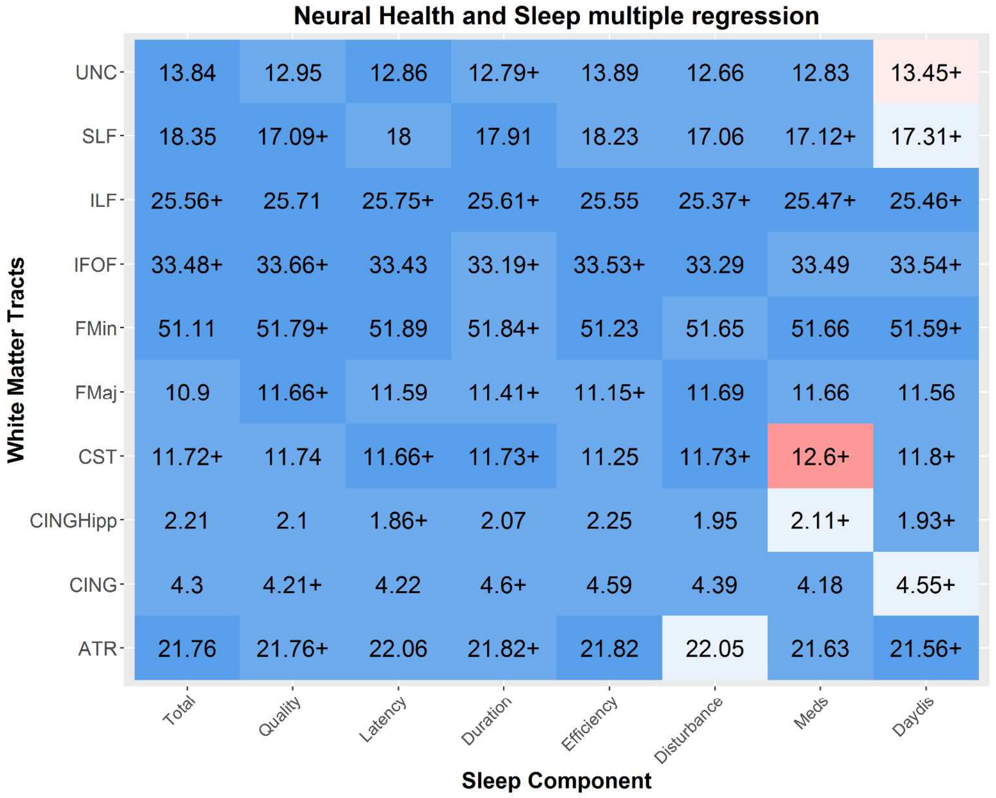

Figure 4. Multiple regressions between sleep components and Neural Health. Each cell represents the relationship between a sleep component and the mean neural health in a given tract as index by Fractional Anisotropy. Numbers represent R-squared, the sample size is show in the last column. Strong associations are observed between measures of Sleep Efficiency and multiple tracts, along with sporadic associations between other components and tracts. White matter tracts abbreviations: Uncinate fasciculus (UNC), superior longitudinal fasciculus (SLF), inferior longitudinal fasciculus (ILF), inferior Fronto-occipital fasciculus (IFOF), forceps minor (FMin), forceps major (FMaj), cerebrospinal tract (CST), the ventral cingulate gyrus (CINGHipp), the dorsal cingulate gyrus (CING), and the anterior thalamic radiations (ATR). N varies slightly across components due to varying missingness (N mean = 631.325, SD = 10.32).

Insert Figure 4 here
254x203mm (300 x 300 DPI)

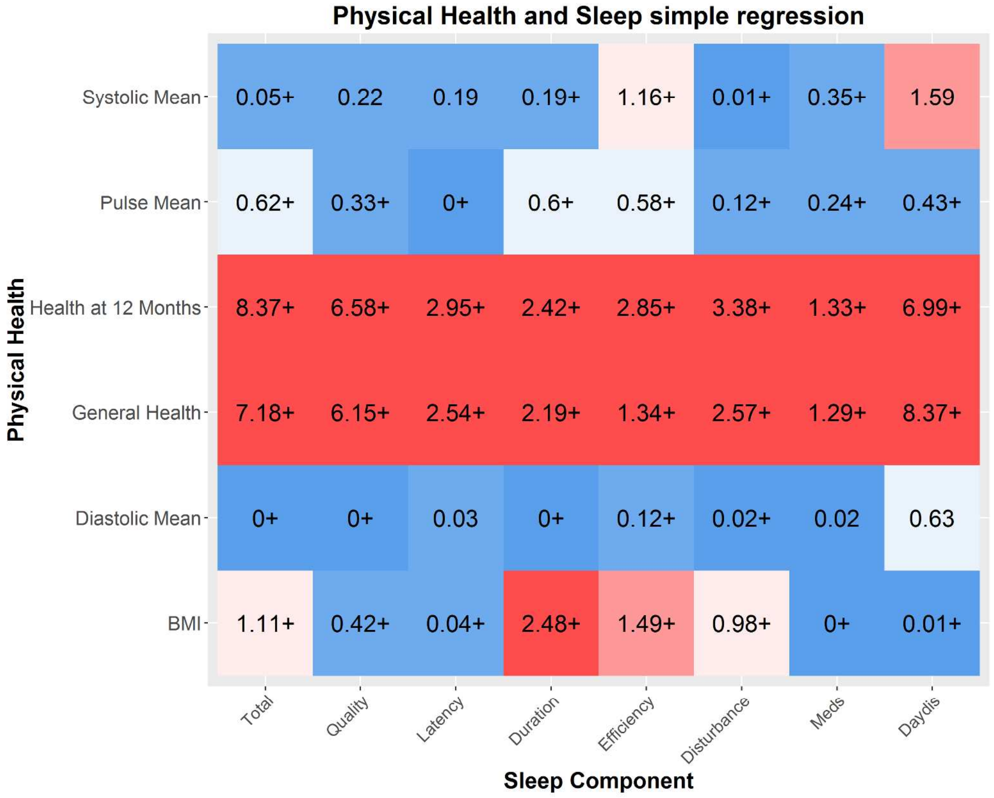

Figure 5 Physical health and sleep quality. Numbers represent Rsquared, the sample size is show in the last column. Strong associations between general indices of health and sleep quality are found, and several more modest relationships with BMI and sleep quality. Self-reported health (12 month and General) were measured in the full cohort (Mean = 2315.37, SD=66.29), the other indicators were measured in the imaging cohort only (Mean = 569.87, SD= 11.16).

Insert Figure 5 here
254x203mm (300 x 300 DPI)

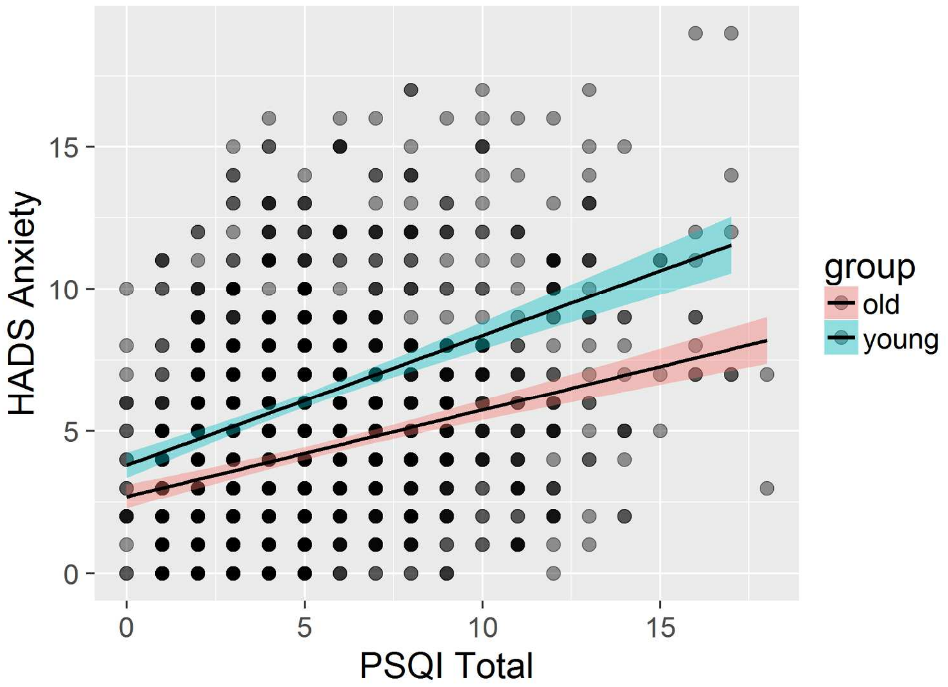

Figure 6. Interaction between sleep quality and anxiety. (N=724, age 18.48 to 46.2) compared to the oldest third of participants (N=725, age 71.79 to 98.88).

Insert Figure 6 here
177x127mm (300 x 300 DPI)

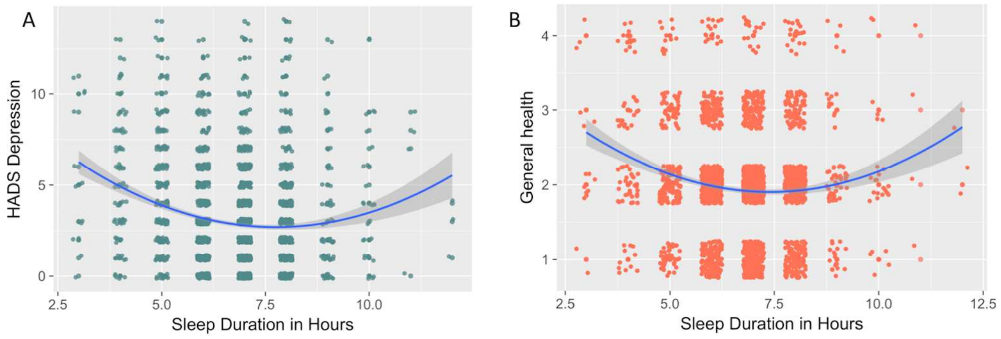

Figure 7. Curvilinear associations between sleep duration in hours and A) HADS depression and B) general health (self-reported). For visual clarity a small amount of random jitter was added to the data points.

Insert Figure 7 here
527x179mm (300 x 300 DPI)

For peer review only

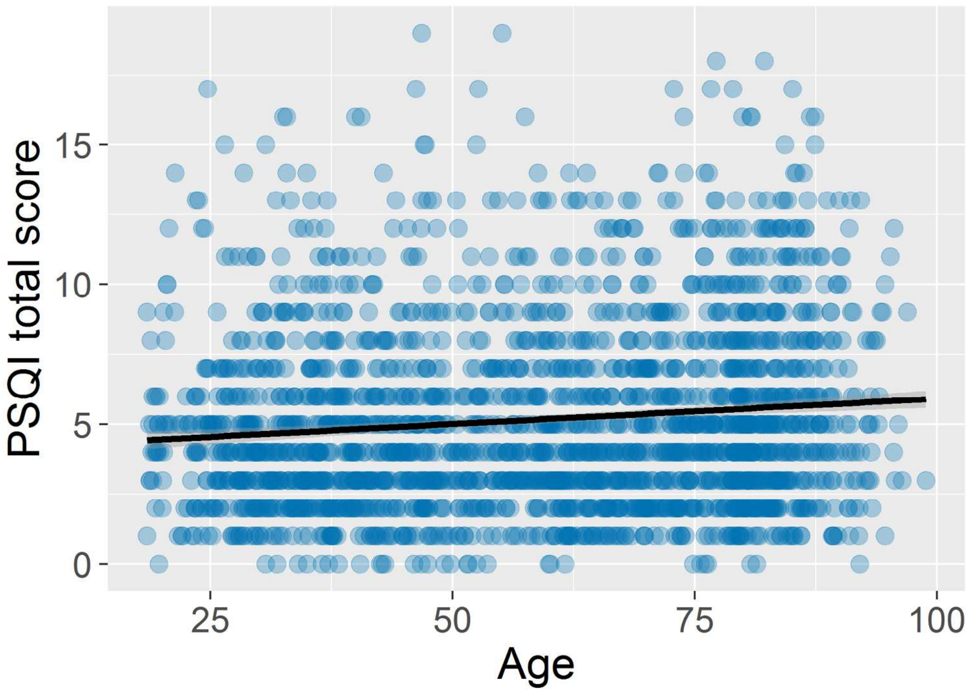

177x127mm (300 x 300 DPI)

iew only

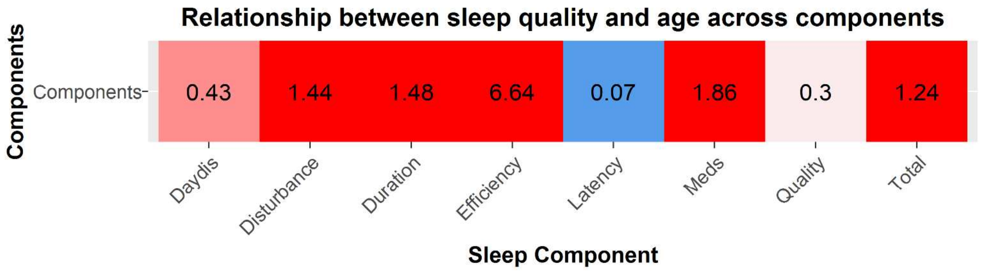

254x127mm (300 x 300 DPI)

For peer review only

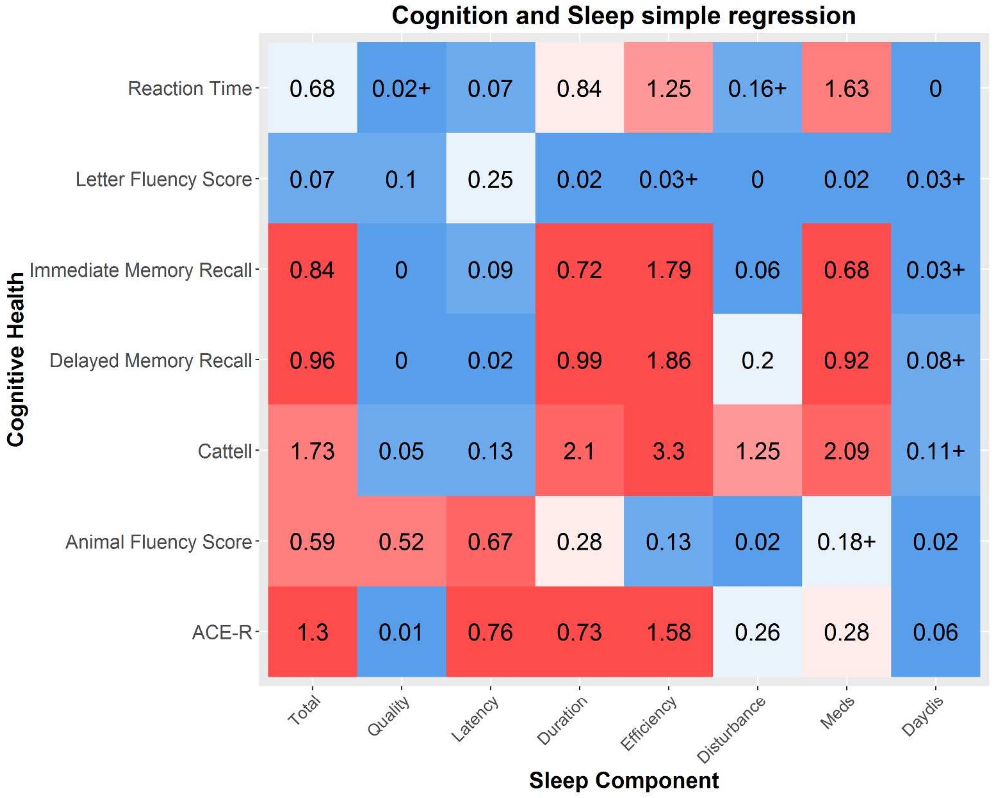

254x203mm (300 x 300 DPI)

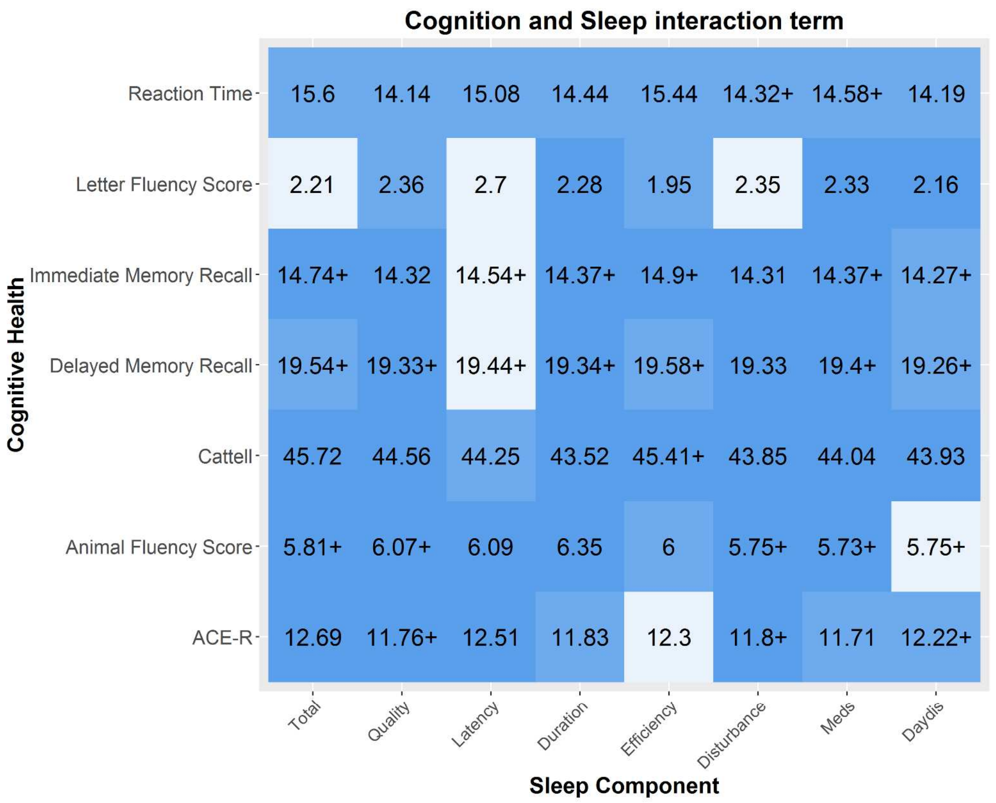

254x203mm (300 x 300 DPI)

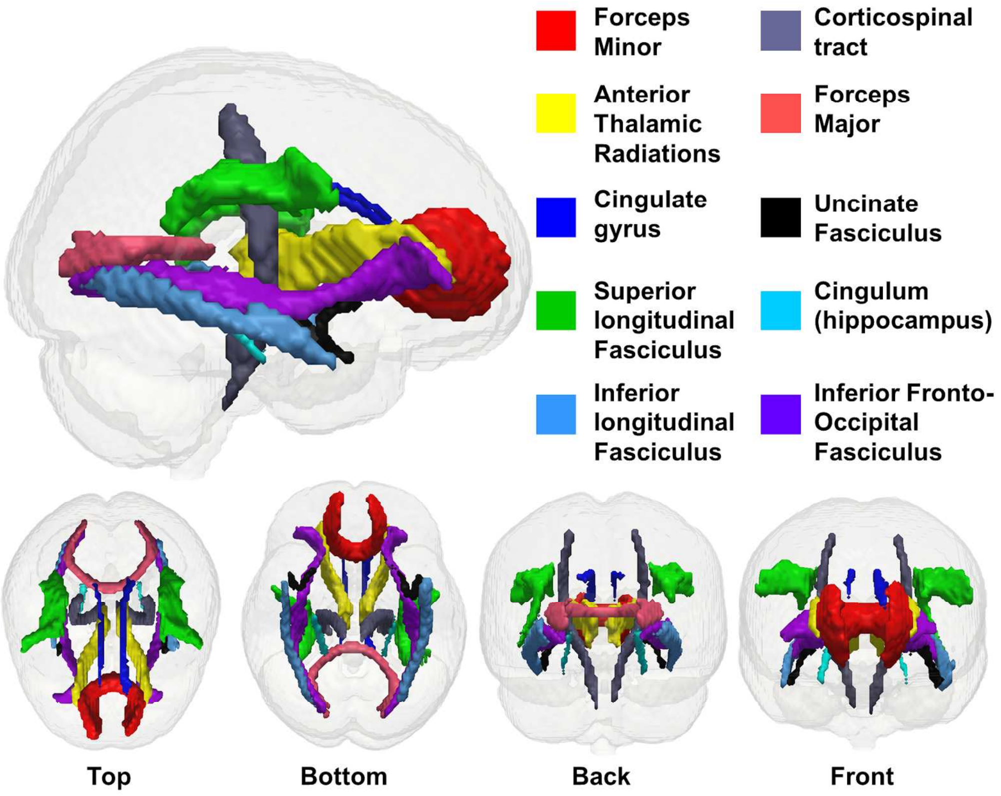

500x400mm (300 x 300 DPI)

For peer review only - http://bmjopen.bmj.com/site/about/guidelines.xhtml

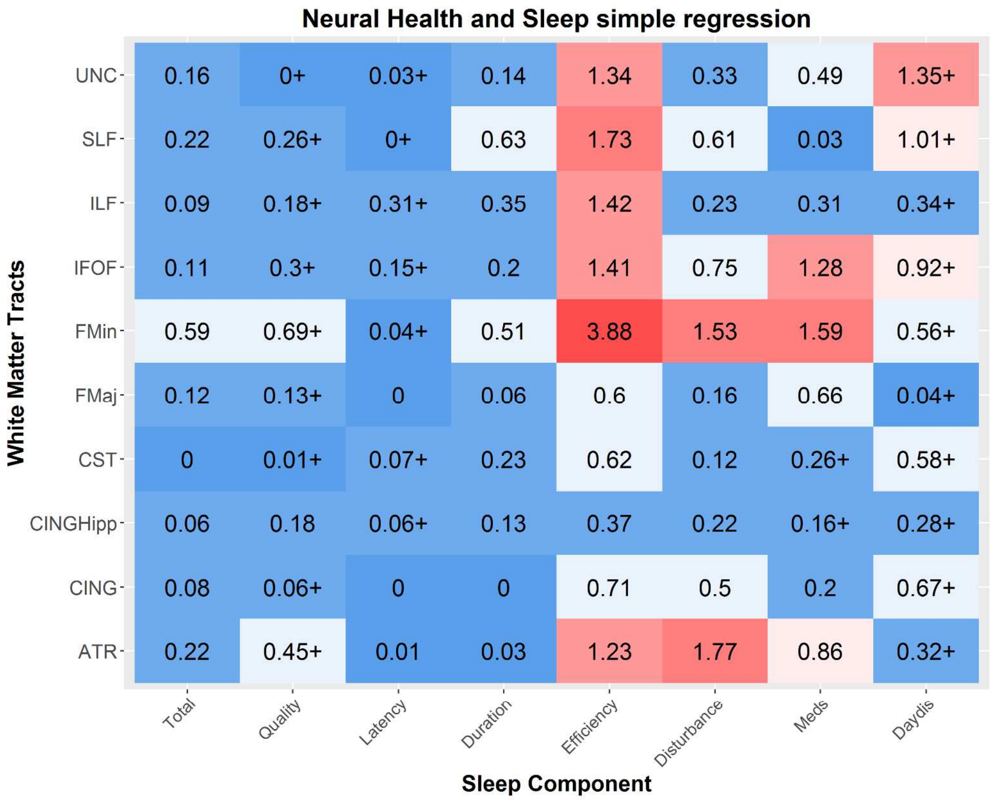

254x203mm (300 x 300 DPI)

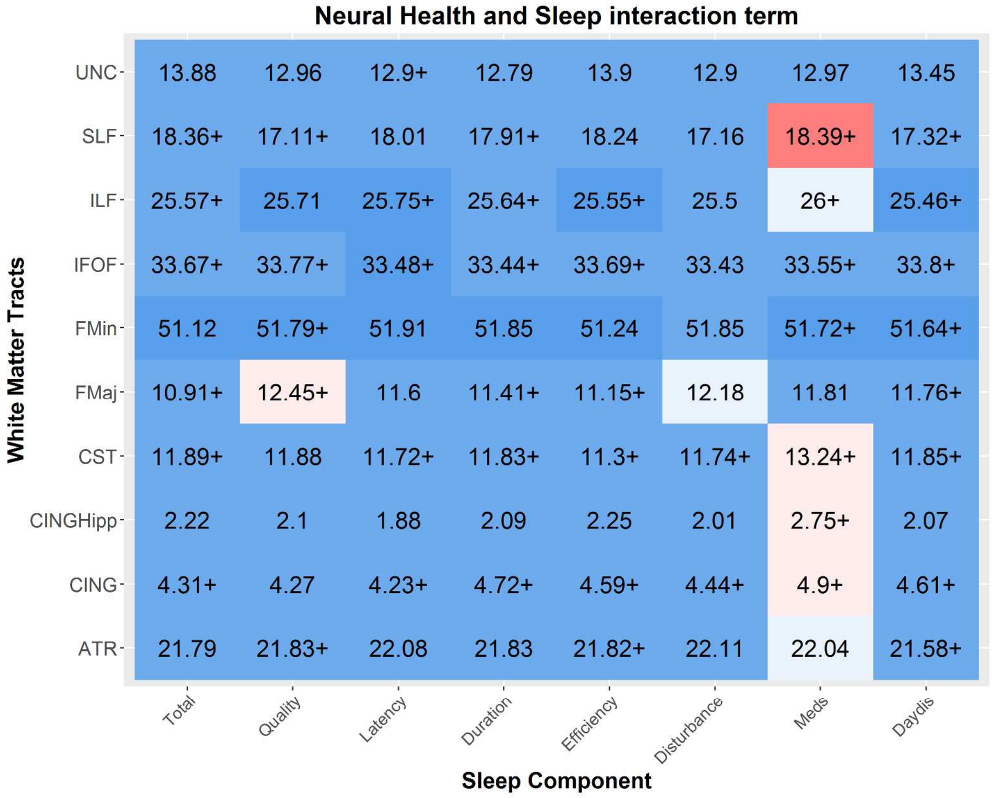

254x203mm (300 x 300 DPI)

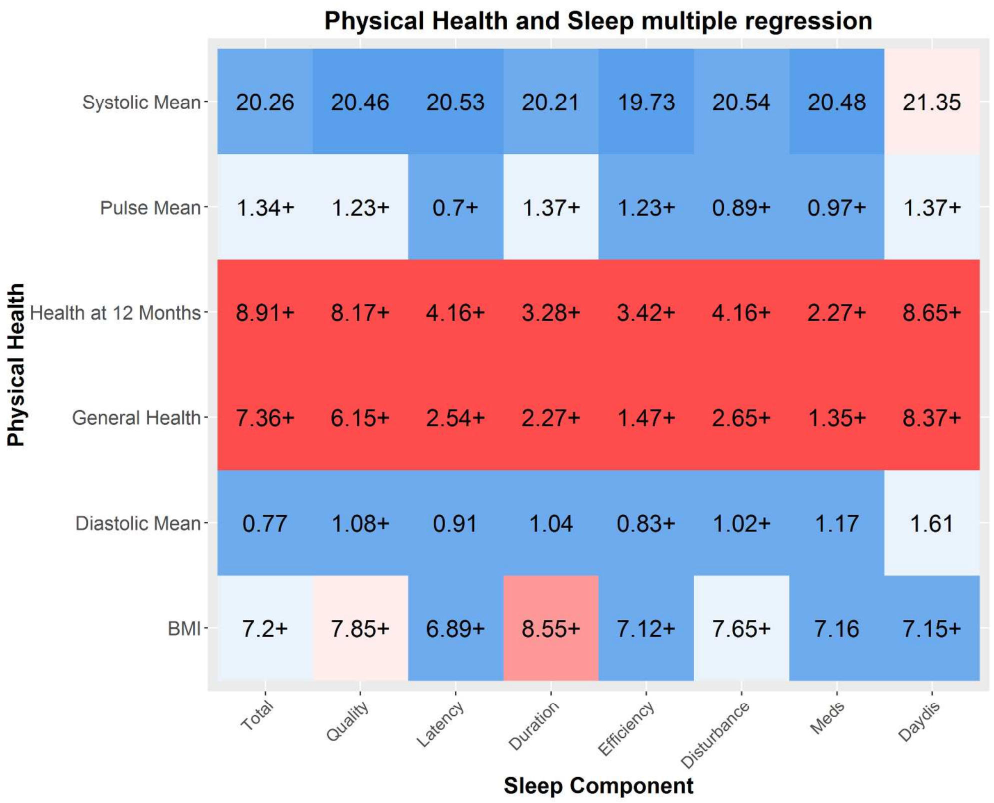

254x203mm (300 x 300 DPI)

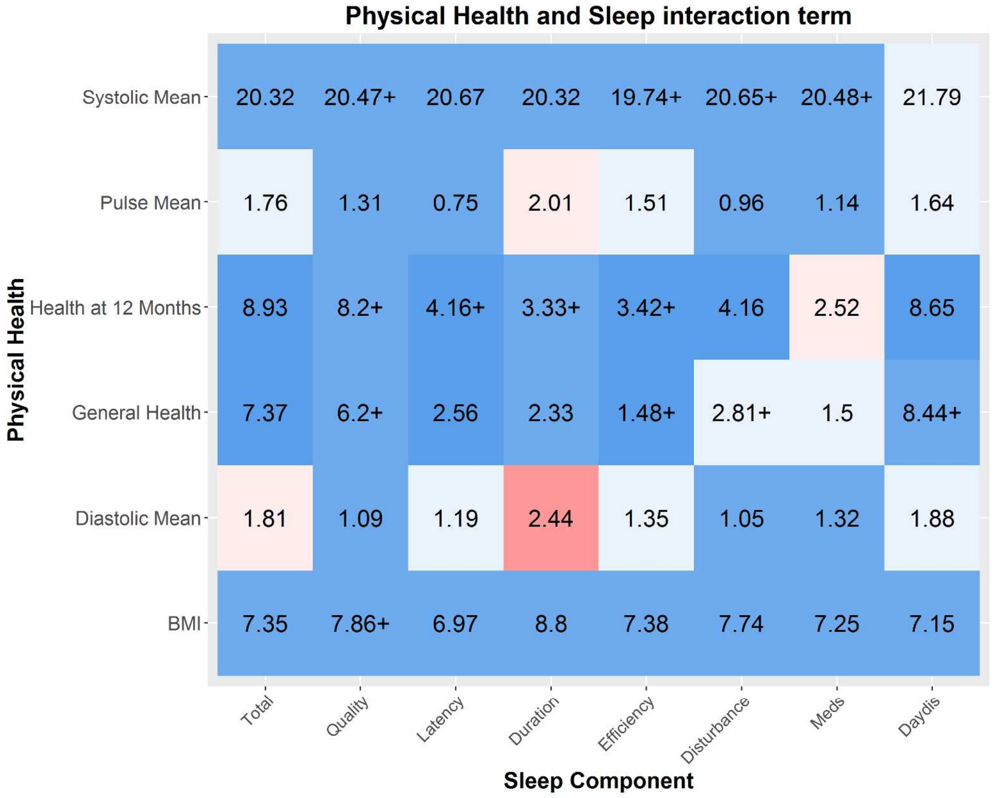

254x203mm (300 x 300 DPI)

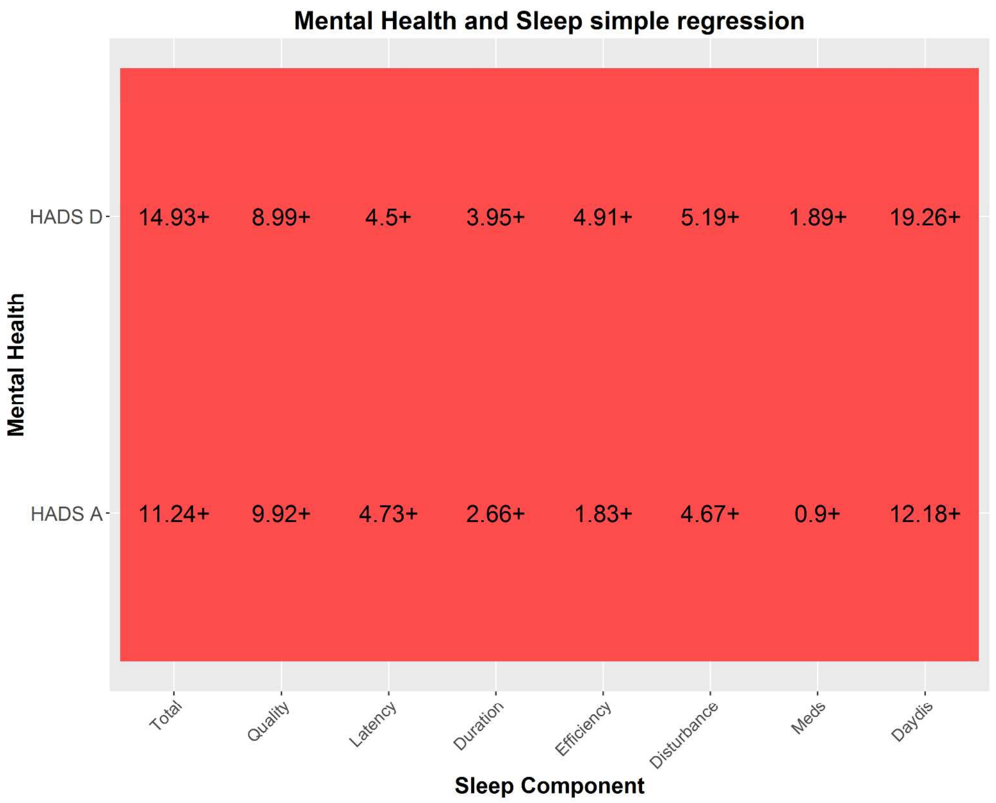

254x203mm (300 x 300 DPI)

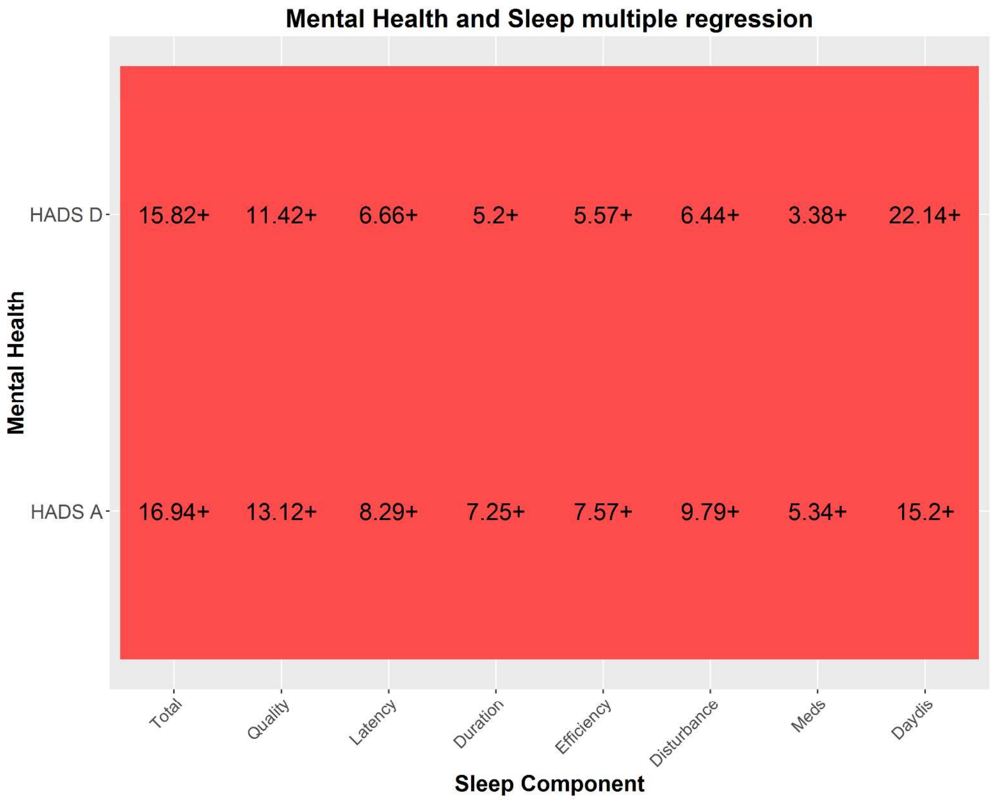

254x203mm (300 x 300 DPI)

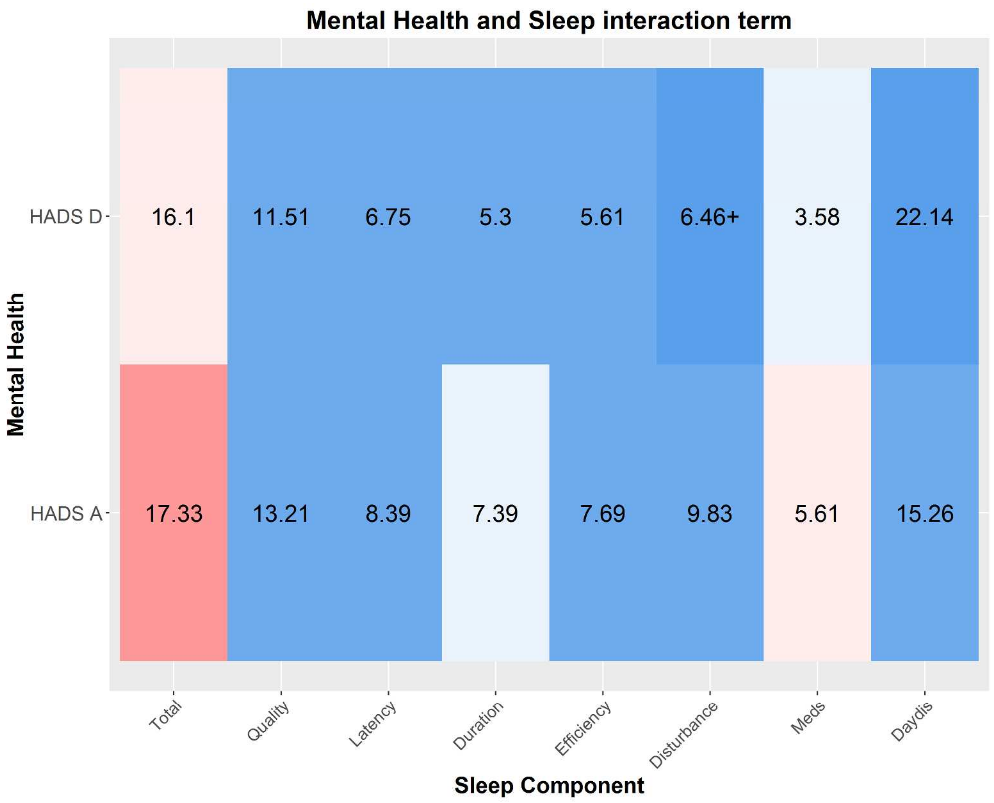

254x203mm (300 x 300 DPI)

For peer review only

STROBE 2007 (v4) Statement—Checklist of items that should be included in reports of *cohort studies*

Section/Topic	Item #	Recommendation	Reported on page #
**Title and abstract**	1	(*a*) Indicate the study's design with a commonly used term in the title or the abstract	1
		(*b*) Provide in the abstract an informative and balanced summary of what was done and what was found	2
**Introduction**			
Background/rationale	2	Explain the scientific background and rationale for the investigation being reported	4
Objectives	3	State specific objectives, including any prespecified hypotheses	5
**Methods**			
Study design	4	Present key elements of study design early in the paper	5
Setting	5	Describe the setting, locations, and relevant dates, including periods of recruitment, exposure, follow-up, and data collection	6
Participants	6	(*a*) Give the eligibility criteria, and the sources and methods of selection of participants. Describe methods of follow-up	6
		(*b*) For matched studies, give matching criteria and number of exposed and unexposed	NA
Variables	7	Clearly define all outcomes, exposures, predictors, potential confounders, and effect modifiers. Give diagnostic criteria, if applicable	5-9
Data sources/ measurement	8*	For each variable of interest, give sources of data and details of methods of assessment (measurement). Describe comparability of assessment methods if there is more than one group	6-11
Bias	9	Describe any efforts to address potential sources of bias	N/A
Study size	10	Explain how the study size was arrived at	6
Quantitative variables	11	Explain how quantitative variables were handled in the analyses. If applicable, describe which groupings were chosen and why	10
Statistical methods	12	(*a*) Describe all statistical methods, including those used to control for confounding	10-13
		(*b*) Describe any methods used to examine subgroups and interactions	10
		(*c*) Explain how missing data were addressed	11
		(*d*) If applicable, explain how loss to follow-up was addressed	N/A
		(*e*) Describe any sensitivity analyses	N/A

For peer review only

**Results**			
Participants	13*	(a) Report numbers of individuals at each stage of study—eg numbers potentially eligible, examined for eligibility, confirmed eligible, included in the study, completing follow-up, and analysed	14-22
		(b) Give reasons for non-participation at each stage	6
		(c) Consider use of a flow diagram	NA
Descriptive data	14*	(a) Give characteristics of study participants (eg demographic, clinical, social) and information on exposures and potential confounders	6
		(b) Indicate number of participants with missing data for each variable of interest	9,10
		(c) Summarise follow-up time (eg, average and total amount)	6
Outcome data	15*	Report numbers of outcome events or summary measures over time	NA
Main results	16	(*a*) Give unadjusted estimates and, if applicable, confounder-adjusted estimates and their precision (eg, 95% confidence interval). Make clear which confounders were adjusted for and why they were included	14-22
		(*b*) Report category boundaries when continuous variables were categorized	NA
		(*c*) If relevant, consider translating estimates of relative risk into absolute risk for a meaningful time period	NA
Other analyses	17	Report other analyses done—eg analyses of subgroups and interactions, and sensitivity analyses	14-22
**Discussion**			
Key results	18	Summarise key results with reference to study objectives	22-26
**Limitations**			
Interpretation	20	Give a cautious overall interpretation of results considering objectives, limitations, multiplicity of analyses, results from similar studies, and other relevant evidence	25-26
Generalisability	21	Discuss the generalisability (external validity) of the study results	25-26
**Other information**			
Funding	22	Give the source of funding and the role of the funders for the present study and, if applicable, for the original study on which the present article is based	3

*Give information separately for cases and controls in case-control studies and, if applicable, for exposed and unexposed groups in cohort and cross-sectional studies.

Note: An Explanation and Elaboration article discusses each checklist item and gives methodological background and published examples of transparent reporting. The STROBE checklist is best used in conjunction with this article (freely available on the Web sites of PLoS Medicine at http://www.plosmedicine.org/, Annals of Internal Medicine at http://www.annals.org/, and Epidemiology at http://www.epidem.com/). Information on the STROBE Initiative is available at www.strobe-statement.org.

For peer review only
